# Genome-wide association analyses of physical activity and sedentary behavior provide insights into underlying mechanisms and roles in disease prevention

**DOI:** 10.1038/s41588-022-01165-1

**Published:** 2022-09-07

**Authors:** Zhe Wang, Andrew Emmerich, Nicolas J. Pillon, Tim Moore, Daiane Hemerich, Marilyn C. Cornelis, Eugenia Mazzaferro, Siacia Broos, Tarunveer S. Ahluwalia, Traci M. Bartz, Amy R. Bentley, Lawrence F. Bielak, Mike Chong, Audrey Y. Chu, Diane Berry, Rajkumar Dorajoo, Nicole D. Dueker, Elisa Kasbohm, Bjarke Feenstra, Mary F. Feitosa, Christian Gieger, Mariaelisa Graff, Leanne M. Hall, Toomas Haller, Fernando P. Hartwig, David A. Hillis, Ville Huikari, Nancy Heard-Costa, Christina Holzapfel, Anne U. Jackson, Åsa Johansson, Anja Moltke Jørgensen, Marika A. Kaakinen, Robert Karlsson, Kathleen F. Kerr, Boram Kim, Chantal M. Koolhaas, Zoltan Kutalik, Vasiliki Lagou, Penelope A. Lind, Mattias Lorentzon, Leo-Pekka Lyytikäinen, Massimo Mangino, Christoph Metzendorf, Kristine R. Monroe, Alexander Pacolet, Louis Pérusse, Rene Pool, Rebecca C. Richmond, Natalia V. Rivera, Sebastien Robiou-du-Pont, Katharina E. Schraut, Christina-Alexandra Schulz, Heather M. Stringham, Toshiko Tanaka, Alexander Teumer, Constance Turman, Peter J. van der Most, Mathias Vanmunster, Frank J. A. van Rooij, Jana V. van Vliet-Ostaptchouk, Xiaoshuai Zhang, Jing-Hua Zhao, Wei Zhao, Zhanna Balkhiyarova, Marie N. Balslev-Harder, Sebastian E. Baumeister, John Beilby, John Blangero, Dorret I. Boomsma, Soren Brage, Peter S. Braund, Jennifer A. Brody, Marcel Bruinenberg, Ulf Ekelund, Ching-Ti Liu, John W. Cole, Francis S. Collins, L. Adrienne Cupples, Tõnu Esko, Stefan Enroth, Jessica D. Faul, Lindsay Fernandez-Rhodes, Alison E. Fohner, Oscar H. Franco, Tessel E. Galesloot, Scott D. Gordon, Niels Grarup, Catharina A. Hartman, Gerardo Heiss, Jennie Hui, Thomas Illig, Russell Jago, Alan James, Peter K. Joshi, Taeyeong Jung, Mika Kähönen, Tuomas O. Kilpeläinen, Woon-Puay Koh, Ivana Kolcic, Peter P. Kraft, Johanna Kuusisto, Lenore J. Launer, Aihua Li, Allan Linneberg, Jian’an Luan, Pedro Marques Vidal, Sarah E. Medland, Yuri Milaneschi, Arden Moscati, Bill Musk, Christopher P. Nelson, Ilja M. Nolte, Nancy L. Pedersen, Annette Peters, Patricia A. Peyser, Christine Power, Olli T. Raitakari, Mägi Reedik, Alex P. Reiner, Paul M. Ridker, Igor Rudan, Kathy Ryan, Mark A. Sarzynski, Laura J. Scott, Robert A. Scott, Stephen Sidney, Kristin Siggeirsdottir, Albert V. Smith, Jennifer A. Smith, Emily Sonestedt, Marin Strøm, E. Shyong Tai, Koon K. Teo, Barbara Thorand, Anke Tönjes, Angelo Tremblay, Andre G. Uitterlinden, Jagadish Vangipurapu, Natasja van Schoor, Uwe Völker, Gonneke Willemsen, Kayleen Williams, Quenna Wong, Huichun Xu, Kristin L. Young, Jian Min Yuan, M. Carola Zillikens, Alan B. Zonderman, Adam Ameur, Stefania Bandinelli, Joshua C. Bis, Michael Boehnke, Claude Bouchard, Daniel I. Chasman, George Davey Smith, Eco J. C. de Geus, Louise Deldicque, Marcus Dörr, Michele K. Evans, Luigi Ferrucci, Myriam Fornage, Caroline Fox, Theodore Garland, Vilmundur Gudnason, Ulf Gyllensten, Torben Hansen, Caroline Hayward, Bernardo L. Horta, Elina Hyppönen, Marjo-Riitta Jarvelin, W. Craig Johnson, Sharon L. R. Kardia, Lambertus A. Kiemeney, Markku Laakso, Claudia Langenberg, Terho Lehtimäki, Loic Le Marchand, Lifelines Cohort Study, Behrooz Z. Alizadeh, H. Marike Boezen, Lude Franke, Morris Swertz, Cisca Wijmenga, Pim van der Harst, Gerjan Navis, Marianne Rots, Bruce H. R. Wolffenbuttel, Patrik K. E. Magnusson, Nicholas G. Martin, Mads Melbye, Andres Metspalu, David Meyre, Kari E. North, Claes Ohlsson, Albertine J. Oldehinkel, Marju Orho-Melander, Guillaume Pare, Taesung Park, Oluf Pedersen, Brenda W. J. H. Penninx, Tune H. Pers, Ozren Polasek, Inga Prokopenko, Charles N. Rotimi, Nilesh J. Samani, Xueling Sim, Harold Snieder, Thorkild I. A. Sørensen, Tim D. Spector, Nicholas J. Timpson, Rob M. van Dam, Nathalie van der Velde, Cornelia M. van Duijn, Peter Vollenweider, Henry Völzke, Trudy Voortman, Gérard Waeber, Nicholas J. Wareham, David R. Weir, Heinz-Erich Wichmann, James F. Wilson, Andrea L. Hevener, Anna Krook, Juleen R. Zierath, Martine A. I. Thomis, Ruth J. F. Loos, Marcel den Hoed

**Affiliations:** 1 The Charles Bronfman Institute for Personalized Medicine, Icahn School of Medicine at Mount Sinai, New York, NY USA; 2 Department of Cell and Molecular Biology, Uppsala University, Uppsala, Sweden; 3 Department of Physiology and Pharmacology, Karolinska Institutet, Stockholm, Sweden; 4 Division of Cardiology, Department of Medicine, University of California, Los Angeles, CA USA; 5 Department of Preventive Medicine, Northwestern University Feinberg School of Medicine, Chicago, IL USA; 6 The Beijer Laboratory and Department of Immunology, Genetics and Pathology, Uppsala University and SciLifeLab, Uppsala, Sweden; 7 Faculty of Movement and Rehabilitation Sciences, Department of Movement Sciences - Exercise Physiology Research Group, KU Leuven, Leuven, Belgium; 8 Faculty of Movement and Rehabilitation Sciences, Department of Movement Sciences - Physical Activity, Sports & Health Research Group, KU Leuven, Leuven, Belgium; 9 Steno Diabetes Center Copenhagen, Herlev, Denmark; 10 Novo Nordisk Foundation Center for Basic Metabolic Research, Faculty of Health and Medical Sciences, University of Copenhagen, Copenhagen, Denmark; 11 The Bioinformatics Center, Department of Biology, University of Copenhagen, Copenhagen, Denmark; 12 Cardiovascular Health Research Unit, Department of Medicine, University of Washington, Seattle, WA USA; 13 Department of Biostatistics, University of Washington, Seattle, WA USA; 14 Center for Research on Genomics and Global Health, National Human Genome Research Institute, National Institutes of Health, Bethesda, MD USA; 15 Department of Epidemiology, School of Public Health, University of Michigan, Ann Arbor, MI USA; 16 Department of Pathology and Molecular Medicine, McMaster University, Hamilton, Ontario Canada; 17 Division of Preventive Medicine, Brigham and Women’s Hospital, Boston, MA USA; 18 GlaxoSmithKline, Cambridge, MA USA; 19 Division of Population, Policy and Practice, Great Ormond Street Hospital Institute for Child Health, University College London, London, UK; 20 Genome Institute of Singapore, Agency for Science, Technology and Research, Singapore, Singapore; 21 Health Services and Systems Research, Duke-NUS Medical School, Singapore, Singapore; 22 John P. Hussman Institute for Human Genomics, University of Miami, Miami, FL USA; 23 Department of Epidemiology & Public Health, University of Maryland School of Medicine, Baltimore, MD USA; 24 Institute for Community Medicine, University Medicine Greifswald, Greifswald, Germany; 25 Institute of Mathematics and Computer Science, University of Greifswald, Greifswald, Germany; 26 Department of Epidemiology Research, Statens Serum Institut, Copenhagen, Denmark; 27 Division of Statistical Genomics, Department of Genetics, Washington University School of Medicine, St. Louis, MO USA; 28 Research Unit of Molecular Epidemiology, Helmholtz Zentrum München –Deutsches Forschungszentrum für Gesundheit und Umwelt (GmbH), Munich, Germany; 29 Department of Epidemiology, University of North Carolina, Chapel Hill, NC USA; 30 Department of Cardiovascular Sciences, University of Leicester, Leicester, UK; 31 NIHR Leicester Biomedical Research Centre, Glenfield Hospital, Leicester, UK; 32 Estonian Genome Centre, Institute of Genomics, University of Tartu, Tartu, Estonia; 33 Postgraduate Program in Epidemiology, Federal University of Pelotas, Pelotas, Brazil; 34 MRC Integrative Epidemiology Unit, NIHR Bristol Biomedical Research Center, University of Bristol, Bristol, UK; 35 Genetics, Genomics, and Bioinformatics Graduate Program, University of California, Riverside, CA USA; 36 Institute of Health Sciences, University of Oulu, Oulu, Finland; 37 Framingham Heart Study, Framingham, MA USA; 38 Department of Neurology, Boston University School of Medicine, Boston, MA USA; 39 Institute for Nutritional Medicine, School of Medicine, Technical University of Munich, Munich, Germany; 40 Department of Biostatistics and Center for Statistical Genetics, University of Michigan, Ann Arbor, MI USA; 41 Department of Immunology, Genetics and Pathology, Science for Life Laboratory, Uppsala University, Uppsala, Sweden; 42 Section of Statistical Multi-omics, Department of Clinical and Experimental Medicine, University of Surrey, Guildford, UK; 43 Department of Metabolism, Digestion and Reproduction, Imperial College London, London, UK; 44 Department of Medical Epidemiology and Biostatistics, Karolinska Institutet, Stockholm, Sweden; 45 Interdisciplinary Program in Bioinformatics, Seoul National University, Seoul, South Korea; 46 Department of Epidemiology, Erasmus MC, University Medical Center Rotterdam, Rotterdam, the Netherlands; 47 University Center for Primary Care and Public Health, University of Lausanne, Lausanne, Switzerland; 48 Swiss Institute of Bioinformatics, Lausanne, Switzerland; 49 Department of Computational Biology, University of Lausanne, Lausanne, Switzerland; 50 Wellcome Sanger Institute, Cambridge, UK; 51 Mental Health and Neuroscience Research Program, QIMR Berghofer Medical Research Institute, Brisbane, Queensland Australia; 52 School of Biomedical Science, Faculty of Medicine, University of Queensland, Brisbane, Queensland Australia; 53 Geriatric Medicine, Institute of Medicine, University of Gothenburg and Sahlgrenska University Hospital Mölndal, Gothenburg, Sweden; 54 Mary MacKillop Institute for Health Research, Australian Catholic University, Melbourne, Victoria Australia; 55 Department of Clinical Chemistry, Fimlab Laboratories, Tampere, Finland; 56 Finnish Cardiovascular Research Center – Tampere, Department of Clinical Chemistry, Faculty of Medicine and Health Technology, Tampere University, Tampere, Finland; 57 Department of Twin Research and Genetic Epidemiology, Kings College London, London, UK; 58 NIHR Biomedical Research Centre at Guy’s and St Thomas’ Foundation Trust, London, UK; 59 Department of Preventive Medicine, Keck School of Medicine, University of Southern California, Los Angeles, CA USA; 60 Department of Kinesiology, Université Laval, Quebec, Quebec Canada; 61 Centre Nutrition Santé et Société (NUTRISS), Institute of Nutrition and Functional Foods (INAF), Université Laval, Quebec, Quebec Canada; 62 Department of Biological Psychology, Vrije Universiteit, Amsterdam, the Netherlands; 63 Amsterdam Public Health Research Institute, Amsterdam UMC, Amsterdam, the Netherlands; 64 MRC Integrative Epidemiology Unit and Avon Longitudinal Study of Parents and Children, University of Bristol Medical School, Population Health Sciences and Avon Longitudinal Study of Parents and Children, University of Bristol, Bristol, UK; 65 Respiratory Division, Department of Medicine, Karolinska Institutet, Karolinska University Hospital, Stockholm, Sweden; 66 Rheumatology Division, Department of Medicine, Karolinska Institutet, Karolinska University Hospital, Stockholm, Sweden; 67 Center of Molecular Medicine (CMM), Karolinska Institutet, Stockholm, Sweden; 68 Department of Health Research Methods, Evidence, and Impact, McMaster University, Hamilton, Ontario Canada; 69 Centre for Global Health Research, Usher Institute, University of Edinburgh, Edinburgh, UK; 70 Department of Clinical Sciences Malmö, Lund University, Malmö, Sweden; 71 Department of Nutrition and Food Sciences, Nutritional Epidemiology, University of Bonn, Bonn, Germany; 72 Translational Gerontology Branch, National Institute on Aging, Baltimore, MD USA; 73 German Centre for Cardiovascular Research (DZHK), partner site Greifswald, Greifswald, Germany; 74 Department of Epidemiology, Harvard T.H. Chan School of Public Health, Boston, MA USA; 75 Department of Epidemiology, University of Groningen, University Medical Center Groningen, Groningen, the Netherlands; 76 Department of Endocrinology, University of Groningen, University Medical Center Groningen, Groningen, the Netherlands; 77 Department of Genetics, University of Groningen, University Medical Center Groningen, Groningen, the Netherlands; 78 MRC Epidemiology Unit, University of Cambridge, Cambridge, UK; 79 School of Public Health, Department of Biostatistics, Shandong University, Jinan, China; 80 Department of Public Health and Primary Care, University of Cambridge, Cambridge, UK; 81 Department of Clinical and Experimental Medicine, University of Surrey, Guilford, UK; 82 People-Centred Artificial Intelligence Institute, University of Surrey, Guilford, UK; 83 University of Münster, Münster, Germany; 84 Diagnostic Genomics, PathWest Laboratory Medicine WA, Perth, Western Australia Australia; 85 South Texas Diabetes and Obesity Institute, University of Texas Rio Grande Valley, Brownsville, TX USA; 86 Lifelines Cohort Study, Groningen, the Netherlands; 87 Department of Sports Medicine, Norwegian School of Sport Sciences, Oslo, Norway; 88 Department of Chronic Diseases, Norwegian Institute of Public Health, Oslo, Norway; 89 Department of Biostatistics, Boston University School of Public Health, Boston, MA USA; 90 Vascular Neurology, Department of Neurology, University of Maryland School of Medicine and the Baltimore VAMC, Baltimore, MD USA; 91 Center for Precision Health Research, National Human Genome Research Institute, NIH, Bethesda, MD USA; 92 Survey Research Center, Institute for Social Research, University of Michigan, Ann Arbor, MI USA; 93 Department of Biobehavioral Health, College of Health and Human Development, Pennsylvania State University, University Park, PA USA; 94 Department of Epidemiology, Institute of Public Health Genetics, Cardiovascular Health Research Unit, University of Washington, Seattle, WA USA; 95 Institute of Social and Preventive Medicine (ISPM), University of Bern, Bern, Switzerland; 96 Radboud Institute for Health Sciences, Department for Health Evidence, Radboud University Medical Center, Nijmegen, the Netherlands; 97 Interdisciplinary Center Psychopathology and Emotion Regulation, University of Groningen, University Medical Center Groningen, Groningen, the Netherlands; 98 School of Population and Global Health, The University of Western Australia, Perth, Western Australia Australia; 99 Busselton Population Medical Research Institute, Busselton, Western Australia Australia; 100 Hannover Unified Biobank, Hannover Medical School, Hannover, Germany; 101 Department of Human Genetics, Hannover Medical School, Hannover, Germany; 102 Centre for Exercise Nutrition & Health Sciences, School for Policy Studies, University of Bristol, Bristol, UK; 103 Department of Pulmonary Physiology and Sleep Medicine, Sir Charles Gairdner Hospital, Western Australia Perth, Australia; 104 Humanity Inc, Boston, MA USA; 105 Department of Clinical Physiology, Tampere University Hospital, Tampere, Finland; 106 Healthy Longevity Translational Research Programme, Yong Loo Lin School of Medicine, National University of Singapore, Singapore, Singapore; 107 Singapore Institute for Clinical Sciences, Agency for Science, Technology, and Research, Singapore, Singapore; 108 Department of Public Health, University of Split School of Medicine, Split, Croatia; 109 Institute of Clinical Medicine, Internal Medicine, University of Eastern Finland and Kuopio University Hospital, Kuopio, Finland; 110 Laboratory of Epidemiology and Population Sciences, National Institutes of Health, Baltimore, MD USA; 111 Center for Clinical Research and Prevention, Bispebjerg and Frederiksberg Hospital, Copenhagen, Denmark; 112 Department of Clinical Medicine, Faculty of Health and Medical Sciences, University of Copenhagen, Copenhagen, Denmark; 113 Division of Internal Medicine, Department of Medicine, Lausanne University Hospital and University of Lausanne, Lausanne, Switzerland; 114 School of Psychology and Faculty of Medicine, University of Queensland, St Lucia, Queensland Australia; 115 Department of Psychiatry, Amsterdam UMC, Vrije Universiteit, Amsterdam, the Netherlands; 116 Institute of Epidemiology, Helmholtz Zentrum München –Deutsches Forschungszentrum für Gesundheit und Umwelt (GmbH), Munich, Germany; 117 Centre for Population Health Research, University of Turku and Turku University Hospital, Turku, Finland; 118 Research Centre of Applied and Preventive Cardiovascular Medicine, University of Turku, Turku, Finland; 119 Department of Clinical Physiology and Nuclear Medicine, Turku University Hospital, Turku, Finland; 120 Department of Epidemiology, University of Washington, Seattle, WA USA; 121 Harvard Medical School, Boston, MA USA; 122 Division of Endocrinology, Diabetes and Nutrition, Department of Medicine, University of Maryland School of Medicine, Baltimore, MD USA; 123 Department of Exercise Science, University of South Carolina, Columbia, SC USA; 124 Division of Research, Kaiser Permanente Northern California, Oakland, CA USA; 125 Icelandic Heart Association, Kópavogur, Iceland; 126 Faculty of Health Sciences, University of the Faroe Islands, Tórshavn, Faroe Islands; 127 Saw Swee Hock School of Public Health, National University of Singapore, Singapore, Singapore; 128 Duke-NUS Medical School, Singapore, Singapore; 129 Department of Medicine, Yong Loo Lin School of Medicine, National University of Singapore, Singapore, Singapore; 130 Department of Medicine, McMaster University, Hamilton, Ontario Canada; 131 Department of Medicine, University of Leipzig, Leipzig, Germany; 132 Department of Internal Medicine, Erasmus University Medical Center, Rotterdam, the Netherlands; 133 Department of Epidemiology and Biostatistics, Amsterdam Public Health Research Institute, VU University Medical Center, Amsterdam, the Netherlands; 134 Interfaculty Institute for Genetics and Functional Genomics, University Medicine Greifswald, Greifswald, Germany; 135 Division of Cancer Control and Population Sciences, UPMC Hillman Cancer Center, University of Pittsburgh, Pittsburgh, PA USA; 136 Department of Epidemiology, Graduate School of Public Health, University of Pittsburgh, Pittsburgh, PA USA; 137 Laboratory of Epidemiology and Population Science, National Instiute on Aging, National Institutes of Health, Bethesda, MD USA; 138 Geriatric Unit, Azienda USL Toscana Centro, Florence, Italy; 139 Human Genomics Laboratory, Pennington Biomedical Research Center, Baton Rouge, LA USA; 140 Population Health Science, Bristol Medical School, NIHR Bristol Biomedical Research Center, University of Bristol, Bristol, UK; 141 Faculty of Movement and Rehabilitation Sciences, Institute of Neuroscience, UC Louvain, Louvain-la-Neuve, Belgium; 142 Department of Internal Medicine B, University Medicine Greifswald, Greifswald, Germany; 143 Brown Foundation Institute of Molecular Medicine, The University of Texas Health Science Center at Houston, Houston, TX USA; 144 Genetics and Pharmacogenomics (GpGx), Merck Research Labs, Boston, MA USA; 145 Department of Evolution, Ecology, and Organismal Biology, University of California, Riverside, Riverside, CA USA; 146 Faculty of Medicine, University of Iceland, Reykjavik, Iceland; 147 MRC Human Genetics Unit, Institute of Genetics and Cancer, University of Edinburgh, Edinburgh, UK; 148 Australian Centre for Precision Health, Unit of Clinical and Health Sciences, University of South Australia, Adelaide, South Australia Australia; 149 South Australian Health and Medical Research Institute, Adelaide, South Australia Australia; 150 Population, Policy and Practice, Great Ormond Street Hospital Institute for Child Health, University College London, London, UK; 151 Department of Epidemiology and Biostatistics and HPA-MRC Center, School of Public Health, Imperial College London, London, UK; 152 Computational Medicine, Berlin Institute of Health at Charité – Universitätsmedizin Berlin, Berlin, Germany; 153 Epidemiology Program, University of Hawaii Cancer Center, University of Hawaii at Manoa, Honolulu, HI USA; 154 K.G.Jebsen Center for Genetic Epidemiology, Norwegian University of Science and Technology, Trondheim, Norway; 155 Department of Genetics, Stanford University School of Medicine, Stanford, CA USA; 156 Center for Fertility and Health, Norwegian Institute of Public Health, Oslo, Norway; 157 Centre for Bone and Arthritis Research, Department of Internal Medicine and Clinical Nutrition, Institute of Medicine, Sahlgrenska Academy, University of Gothenburg, Gothenburg, Sweden; 158 Department of Drug Treatment, Sahlgrenska University Hospital, Gothenburg, Sweden; 159 Department of Statistics, Seoul National University, Seoul, South Korea; 160 University of Split School of Medicine, Split, Croatia; 161 UMR 8199 – EGID, Institut Pasteur de Lille, CNRS, University of Lille, Lille, France; 162 Department of Public Health, Section of Epidemiology, Faculty of Health and Medical Sciences, University of Copenhagen, Copenhagen, Denmark; 163 MRC Integrative Epidemiology Unit, University of Bristol Medical School, University of Bristol, Bristol, UK; 164 Department of Exercise and Nutrition Sciences, Milken Institute School of Public Health, George Washington University, Washington, DC USA; 165 Section of Geriatrics, Department of Internal Medicine, Amsterdam UMC, University of Amsterdam, Amsterdam, the Netherlands; 166 Amsterdam Public Health, Aging and Later Life, Amsterdam, the Netherlands; 167 Nuffield Department of Population Health, University of Oxford, Oxford, UK; 168 Division of Endocrinology, Department of Medicine, University of California, Los Angeles, CA USA; 169 Department of Molecular Medicine and Surgery, Karolinska Institutet, Stockholm, Sweden; 170 The Mindich Child Health and Development Institute, Icahn School of Medicine at Mount Sinai, New York, NY USA; 171 Department of Cardiology, University of Groningen, University Medical Center Groningen, Groningen, the Netherlands; 172 Department of Internal Medicine, Division of Nephrology, University of Groningen, University Medical Center Groningen, Groningen, the Netherlands; 173 Department of Medical Biology, University of Groningen, University Medical Center Groningen, Groningen, the Netherlands

**Keywords:** Genome-wide association studies, Genetics research, Translational research

## Abstract

Although physical activity and sedentary behavior are moderately heritable, little is known about the mechanisms that influence these traits. Combining data for up to 703,901 individuals from 51 studies in a multi-ancestry meta-analysis of genome-wide association studies yields 99 loci that associate with self-reported moderate-to-vigorous intensity physical activity during leisure time (MVPA), leisure screen time (LST) and/or sedentary behavior at work. Loci associated with LST are enriched for genes whose expression in skeletal muscle is altered by resistance training. A missense variant in *ACTN3* makes the alpha-actinin-3 filaments more flexible, resulting in lower maximal force in isolated type II_A_ muscle fibers, and possibly protection from exercise-induced muscle damage. Finally, Mendelian randomization analyses show that beneficial effects of lower LST and higher MVPA on several risk factors and diseases are mediated or confounded by body mass index (BMI). Our results provide insights into physical activity mechanisms and its role in disease prevention.

## Abstract (web summary)

Multi-ancestry meta-analyses of genome-wide association studies for self-reported physical activity during leisure time, leisure screen time, sedentary commuting and sedentary behavior at work identify 99 loci associated with at least one of these traits.

## Main

Low levels of physical activity have a major effect on disease burden and it is estimated that more than 5 million deaths per year might be prevented by ensuring adequate levels^1^. Despite efforts to increase physical activity levels^2^, an estimated 28% of the world's population is insufficiently active, and the prevalence of physical inactivity in high-income countries rose from 31.6% in 2001 to 36.8% in 2016 (ref. ^3^). Trends of decreasing physical activity levels over time coincide with increases in the time spent sedentary^4^, which may pose an independent risk for public health^5,6^.

Physical activity and sedentary behavior are affected by public policy and social support, as well as by cultural, environmental and individual factors^7^. Factors like socioeconomic status, built environment and media all influence physical activity at a population level^7^. In parallel, innate biological factors (for example, age, sex hormones, pre-existing medical conditions, epigenetics and genetics) also explain a moderate proportion of the interindividual variability in physical activity and sedentary behavior. Heritability estimates (*h*^2^) range from 31% to 71% in large twin studies^8,9^. Identifying the genetic factors that influence daily physical activity will improve our understanding of this complex behavior, and may (1) facilitate unbiased causal inference; (2) help identify vulnerable subpopulations; and (3) fuel the design of tailored interventions to effectively promote physical activity. A mechanistic understanding of physical activity at a molecular level may even allow its beneficial effects to be attained through pharmacological intervention^10^.

Genome-wide association studies (GWAS) have identified thousands of loci associated with cardiometabolic risk factors and diseases^11^. However, similar efforts for physical activity have been sparse and initially had limited success. This likely reflects the comparatively small sample size of these efforts^12^, along with heterogeneous assessments of physical activity across studies. More recently, GWAS using data from UK Biobank identified nine loci associated with self-reported moderate and/or vigorous intensity physical activity or sports and exercise participation (*n* ≈ 377,000 individuals) and eight associated with accelerometry-assessed physical activity and sedentary behavior (*n* ≈ 91,000)^13,14^. Hence, on the assumption that physical activity is a highly polygenic trait, many common variants influencing physical activity undoubtedly remain to be identified.

Here, we combine data from up to 703,901 individuals (94.0% European, 2.1% African, 0.8% East Asian, 1.3% South Asian ancestries, and 1.9% Hispanic) from 51 studies in a multi-ancestry meta-analysis of GWAS for MVPA, LST, sedentary commuting and sedentary behavior at work. This yields 104 independent association signals in 99 loci, implicating brain and muscle, among others organs. Follow-up analyses improve our understanding of the molecular basis of leisure time physical activity and sedentary behavior, and their role in disease prevention.

## Results

### Genome-wide analyses yield 99 associated loci

**Fig. 1:**
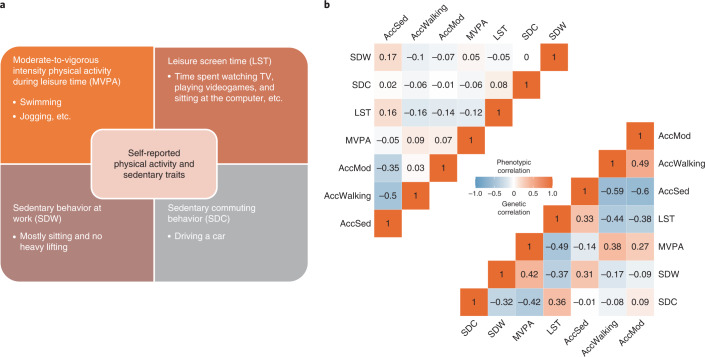
Overview of the four self-reported physical activity and sedentary traits and correlations with objectively assessed traits. **a**, An overview of the four self-reported physical activity and sedentary traits. **b**, Phenotypic (upper left) and genetic (lower right) correlation coefficients between the four self-reported physical activity and sedentary traits studied here and three accelerometer-assessed traits quantified in UK Biobank participants. AccMod, accelerometer-assessed proportion of time spent in moderate intensity physical activity; AccSed, accelerometer-assessed proportion of time spent sedentary; AccWalking, accelerometer-assessed proportion of time spent walking; SDC, sedentary commuting behavior; SDW, sedentary behavior at work.

**Fig. 2:**
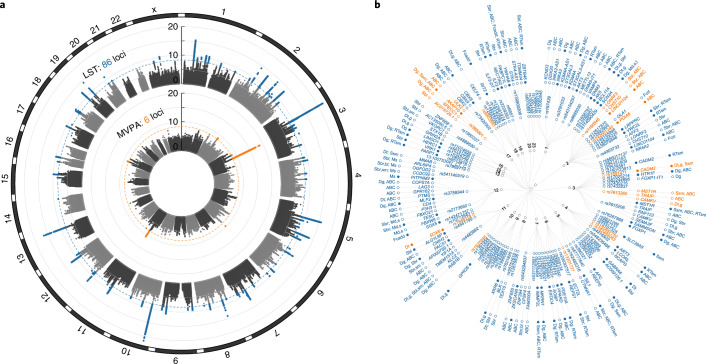
Main results of GWAS and downstream gene prioritization for LST and MVPA. **a**, Circular Manhattan plot summarizing the results from European ancestry meta-analyses for LST and MVPA. Outer track, LST; inner track, MVPA. Genome-wide significant variants (*P* < 5 × 10^−9^) are highlighted in orange for loci associated with MVPA and in blue for loci associated with LST. **b**, Dendrogram showing the 101 independent association signals in LST- and MVPA-associated loci from European ancestry or multi-ancestry meta-analyses. Moving outwards from the center are: (1) chromosome; (2) lead SNP identifiers, in orange for loci associated with MVPA, in blue for loci associated with LST; (3) the most promising gene(s) prioritized in the locus (closest genes are highlighted by filled circles); and (4) the approach(es) by which the gene was prioritized, that is, DEPICT gene prioritization (Dg) or tissue enrichment (Dt); SMR of eQTL signals in blood (Sbl), brain (Sbr) or skeletal muscle (Ssm); credible variants identified by FINEMAP that (i) are coding and likely to have a detrimental effect on protein function (Fcadd) or (ii) show evidence of three-dimensional interactions with the candidate gene in central nervous system cell types (Fcrt); activity-by-contact (ABC) in 26 relevant tissues and cell types; a contribution to enrichment for altered expression in skeletal muscle following a resistance training intervention (RTsm); and/or proximity to an association signal for spontaneous running speed (Ms), time run (Mt) or distance run (Md) in a GWAS of 100 inbred mouse strains.

In our primary meta-analysis of European ancestry men and women combined (Supplementary Tables 1, 2), we identify 91 loci that are associated (*P* < 5 × 10^−9^) with at least one of four self-reported traits: MVPA (*n* up to 606,820), LST (*n* up to 526,725), sedentary commuting (*n* up to 159,606) and sedentary behavior at work (*n* up to 372,605) (Supplementary Table 3, Figs. 1 and 2, and Supplementary Fig. 1). The non-European ancestry meta-analyses do not provide new associations themselves and are only used in multi-ancestry meta-analyses. Multi-ancestry and sex-specific meta-analyses yield eight additional loci, resulting in a total of 104 independent association signals in 99 loci (Supplementary Tables 3 and 4). The vast majority of these—89 independent single nucleotide polymorphisms (SNPs) in 88 loci (35 not previously reported^13,15^)—are associated with LST, explaining 2.75% of its variance. We also identify 11 loci for MVPA (six not previously reported^13,15,16^, four that overlap with LST) and four loci for sedentary behavior at work (all previously reported^13,15^; Supplementary Table 3). No loci are identified for sedentary commuting. To increase statistical power for the discovery of new loci, we perform a multi-trait analysis of GWAS (MTAG) using summary statistics of MVPA and LST. This yields 13 additional loci: eight loci for MVPA and eight for LST, with three loci overlapping (Supplementary Table 5)^17^.

SNP-heritability estimates range from 8% for MVPA to 16% for LST (Supplementary Table 6 and Methods). Genetic correlations between the four traits range from −0.32 for sedentary behavior at work and sedentary commuting, to −0.49 for LST and MVPA (Fig. 1b). To ensure adequate statistical power in instrumental variable and enrichment analyses, we focus on LST and MVPA from here onwards.

Genetic correlations of self-reported LST and MVPA with objective, accelerometry-assessed daily physical activity traits in UK Biobank range from 0.14 to 0.44 (Fig. 1b). Importantly, five of the eight loci previously identified for objectively assessed daily physical activity in UK Biobank data^13,14^ show directionally consistent associations (*P* < 0.05) with self-reported LST and/or MVPA in our study (Supplementary Table 7). By contrast, 39 LST- and 4 MVPA-associated loci observed here show directionally consistent associations (*P* < 0.05) with at least one objectively assessed physical activity and/or sedentary trait (using accelerometry) in UK Biobank (Supplementary Table 8). In line with this, each additional LST-decreasing and MVPA-increasing allele in unweighted genetic predisposition scores of the 88 LST- and 11 MVPA-associated loci, respectively, are associated with higher objectively assessed daily physical activity levels in UK Biobank (*P* = 5 × 10^−23^ for LST; *P* = 2 × 10^−3^ for MVPA, Supplementary Table 8).

**Fig. 3:**
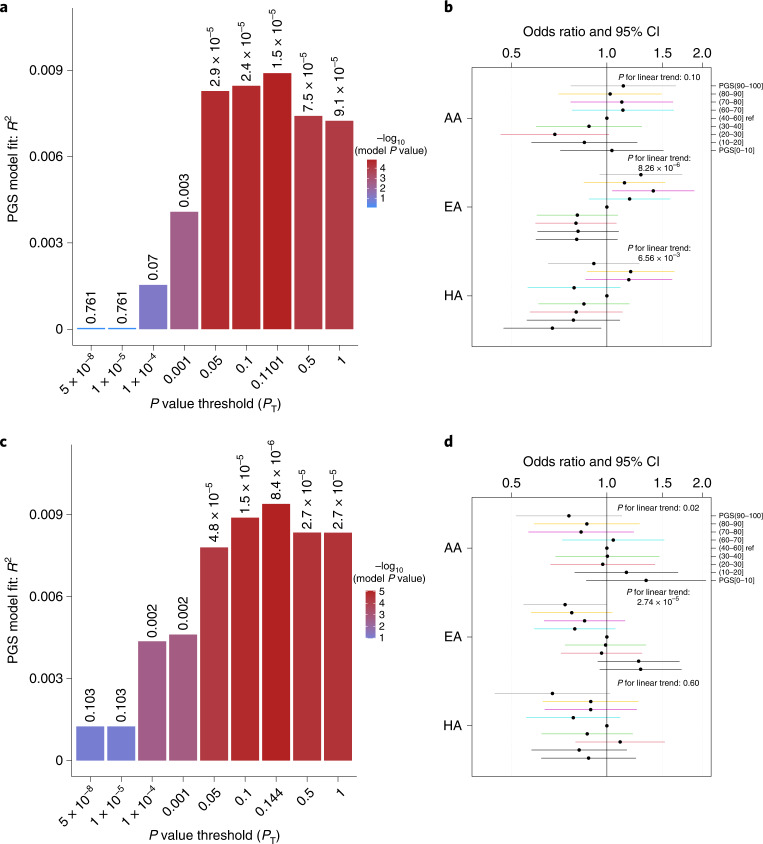
Validation of associations with MVPA and LST using PGSs in Bio*Me* participants of three ancestries. **a**,**c**, The best performing PGSs for MVPA (**a**) and LST (**c**) were derived using logistic/linear regression analyses; that is, those with the highest incremental *R*^2^ above and beyond models with only sex, age and the top ten principal components. This was accomplished using inclusion thresholds of *P* < 0.1101 for MVPA and *P* < 0.14 for LST. **b**,**d**, The association—examined using a logistic regression analysis—of MVPA with the PGSs for MVPA (**b**) and LST (**d**) in individuals of African (AA, *n* = 2,224), European (EA, *n* = 2,765) and Hispanic (HA, *n* = 3,206) ancestry in data from the Bio*Me* BioBank. Dots and error bars show OR and 95% CI.

As external validation, we use the European ancestry summary statistics of LST and MVPA to construct polygenic scores (PGSs), and examine their associations with MVPA in 8,195 Bio*Me* BioBank participants of European (*n* = 2,765), African (*n* = 2,224) and Hispanic (*n* = 3,206) ancestry. In general, a higher PGS for MVPA is associated with higher odds of engaging in more than 30 min per week of MVPA, and a higher PGS for LST with lower odds of engaging in MVPA. Individuals at the highest decile of the PGS for LST are 26% less likely to spend more than 30 min per week on MVPA compared with individuals at deciles 4 to 6 (odds ratio (OR) [95% confidence intervals (CI)] = 0.74 [0.55–0.99]) (Fig. 3 and Supplementary Table 9).

### Shared genetic architecture

**Fig. 4:**
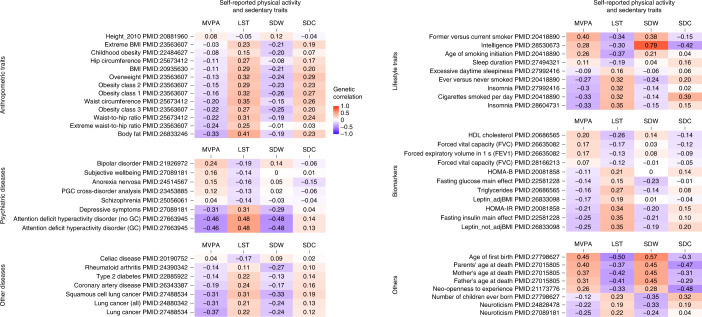
Genetic correlations of four self-reported physical activity traits with complex traits and diseases. Results are based on published GWAS with *P* < 4.6 × 10^−4^ for at least one physical activity or sedentary trait. Darker colors reflect higher negative (purple) or positive (red) correlation coefficients. GC, genomic control; HDL, high-density lipoprotein; HOMA-B, homeostasis model assessment of beta-cell function; HOMA-IR, homeostatasis model assessment of insulin resistance; PGC, psychiatric genomics consortium.

Using linkage disequilibrium (LD) score regression implemented in the LD-Hub^18^, we observe significant (*P* < 4.6 × 10^−4^) genetic correlations of LST and MVPA with adiposity-related traits (*r* = −0.41 to −0.20), especially with body fat percentage (*r*_g_ = 0.4 and −0.3, respectively; Fig. 4, Supplementary Fig. 2 and Supplementary Table 10). In line with moderate genetic correlations, 11 of the 99 self-reported loci for physical activity and sedentary behavior have previously been associated with obesity-related traits^19–25^. In addition, PGSs for lower LST and higher MVPA are associated with lower BMI in up to 23,723 participants from the Bio*Me* BioBank (Supplementary Table 9), and a phenome-wide association study (PheWAS) in 8,959 Bio*Me* European ancestry samples shows a negative association between the PGS for MVPA and morbid obesity (*P* = 1.1 × 10^−5^, Supplementary Fig. 3). Strikingly, genetic correlations with body fat percentage are similar for self-reported LST, MVPA (Fig. 4) and accelerometer-assessed physical activity traits^13,14^ (Supplementary Fig. 2).

Besides adiposity, less sedentary behavior and higher physical activity levels are also genetically correlated with a more favorable cardiometabolic status, including lower triglyceride, total cholesterol, fasting glucose and fasting insulin levels, and lower odds of type 2 diabetes and coronary artery disease; as well as with better mental health outcomes, a lower risk of lung cancer and with longevity (Fig. 4 and Supplementary Fig. 2).

### Causal inference

**Fig. 5:**
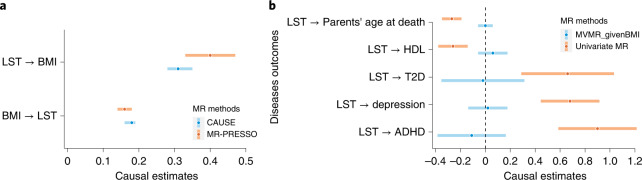
MR analyses between LST, MVPA, BMI and complex diseases. **a**, Median causal estimates for MR analyses using the CAUSE method and causal estimates from the MR-PRESSO method after outlier removal and accounting for horizontal pleiotropy. **b**, The causal effects of LST on complex risk factors and diseases without (in orange) and with (in blue) adjusting for BMI. Dots and error bars show the estimated causal effect sizes and 95% CI. ADHD, attention deficit hyperactivity disorder; T2D, type 2 diabetes.

To assess directions of causality between sedentary behavior/physical activity and BMI, we next perform two-sample Mendelian randomization (MR) analyses using multiple MR methods that utilize genome-wide full summary results or genome-wide significant loci (Supplementary Table 11 and Methods)^26–30^. Causal Analysis Using Summary Effect Estimates (CAUSE)^26^ as well as traditional MR methods consistently show that LST and BMI causally affect each other, with the causal effect (the per 1 s.d. unit increase in each trait) of higher LST on higher BMI being two- to threefold larger than the effect of BMI on LST (Fig. 5a, Table 1 and Supplementary Table 11). Results are similar for bidirectional causal inference tests using body fat percentage instead of BMI (Table 2). However, CAUSE cannot distinguish a model of causality from horizontal pleiotropy for body fat percentage and LST (Table 2). CAUSE also illustrates a causal effect of higher LST on higher recalled adiposity and height in childhood (Table 2), supporting our hypothesis that a genetic predisposition for higher LST later in life represents a lifelong predisposition that already influences adiposity through sedentary behavior early in life. We observe similar evidence for causal effects between MVPA and adiposity, with smaller effects when compared with LST.

**Table 1:** Bidirectional MR results for LST and MVPA with BMI or body fat percentage using significant loci only

Exposure	Outcome	Beta	s.e.	*P* value	Exposure	Outcome	Beta	s.e.	*P* value
LST	Body fat %	0.16	0.07	0.016	LST	BMI	0.40	0.04	8.4 × 10^−14^
Body fat %	LST	0.12	0.03	0.005	BMI	LST	0.16	0.01	1.4 × 10^−74^
MVPA	Body fat %	−0.21	0.17	0.22	MVPA	BMI	−0.25	0.04	0.002
Body fat %	MVPA	−0.001	0.036	0.97	BMI	MVPA	−0.10	0.01	5.8 × 10^−12^

We use MR-PRESSO with outliers removed for all pairs of traits except for the causal effect estimation between body fat percentage (body fat %) and MVPA because no outliers were detected by MR-PRESSO. For body fat percentage → MVPA, we reported the causal estimates using an inverse variance-weighted test; for MVPA → body fat percentage, we reported the weighted median method because these two methods were selected by the machine learning framework (Methods) to be the most appropriate approaches for each analysis, respectively. P < 0.0125 indicates significant effects.

**Table 2:** Bidirectional MR results for LST and MVPA during leisure time with BMI or body fat percentage using genome-wide summary results (CAUSE method)

Exposure	Outcome	Gamma^a^	95% CI	*P* value^b^	Exposure	Outcome	Gamma^a^	95% CI	*P* value^b^
LST	Body fat %	0.18	0.13 to 0.24	1.8 × 10^−3^	LST	BMI	0.31	0.28 to 0.35	6.7 × 10^−28^
Body fat %	LST	0.12	0.04 to 0.18	0.14	BMI	LST	0.18	0.16 to 0.19	1.1 × 10^−14^
MVPA	Body fat %	−0.12	−0.20 to −0.04	0.07	MVPA	BMI	−0.14	−0.20 to −0.07	6.0 x 10^−3^
Body fat %	MVPA	−0.03	−0.09 to 0.02	0.53	BMI	MVPA	−0.09	−0.11 to −0.06	7.4 x 10^−3^
LST	Comparative height at age 10	0.03	0.01 to 0.04	0.04	LST	Comparative body size at age 10	0.02	0.01 to 0.03	0.04

^a^Posterior median of gamma, which can be taken as a point estimate of the causal effect. This estimate tends to be shrunk slightly toward zero compared with other methods. ^b^The *P* value for comparing the causal model with the sharing model. *P* < 0.05 indicates that posteriors estimated under the causal model predict the data significantly better than posteriors estimated under the sharing model.

We next investigate the causal effects of LST and MVPA on common diseases and risk factors, with and without adjusting for BMI (Supplementary Tables 12 and 13). In univariate analyses, we observe effects of lower LST on higher high-density lipoprotein cholesterol levels, higher parental age at death, and on lower odds of type 2 diabetes, attention deficit hyperactivity disorder and depression. The CAUSE model only supports evidence for a causal effect of LST on attention deficit hyperactivity disorder and parental age at death. Importantly, multivariable MR analyses show that all protective causal effects of lower LST are either mediated or confounded by BMI.

Directions of causal effects are consistent across LST and MVPA, but only reach significance for MVPA on parental age at death when using the CAUSE model. As for LST, multivariable MR results suggest that the protective causal effects of higher MVPA are either mediated or confounded by BMI, but results should be interpreted with caution for MVPA because of weak instrument bias (conditional *F* statistics <10)^31^ (Fig. 5b and Supplementary Table 13).

### Gene expression in skeletal muscle following training

**Extended Data Fig. 1:**
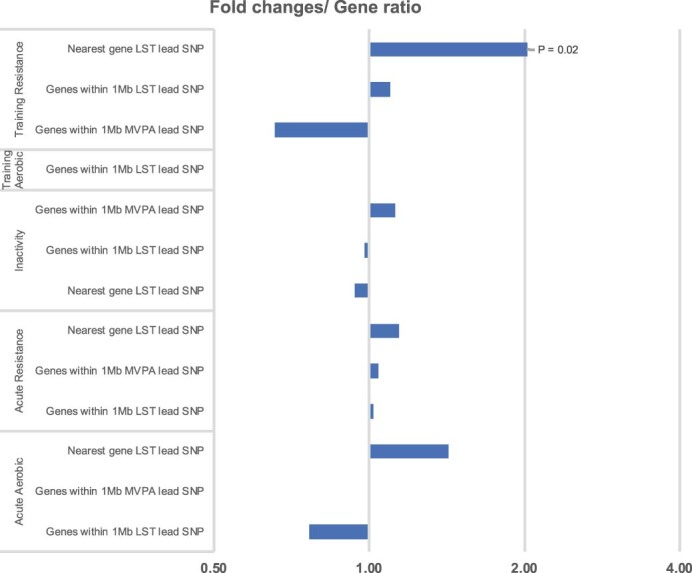
LST-associated loci are enriched for genes with altered expression in skeletal muscle following resistance training. Fold-change plot in log scale for the ratio between: (1) the proportion of genes in physical activity-associated loci that showed an altered expression in skeletal muscle (FDR < 0.01) across five categories: inactivity, acute bout of resistance exercise, acute bout of aerobic exercise, resistance training, or aerobic training; and (2) the proportion of all genes that showed an altered expression following such (in)activity in the MetaMex database (PMID: 31980607). Tested loci were MVPA or LST-associated loci. In a given set of loci, we either considered only the genes nearest to the lead SNP, or all genes within 1 Mb of the lead SNP. Only loci harboring at least five genes with altered gene expression levels after intervention were included in this figure. A one-sided Fisher exact test was used to calculate the *P*-value for enrichment.

**Extended Data Fig. 2:**
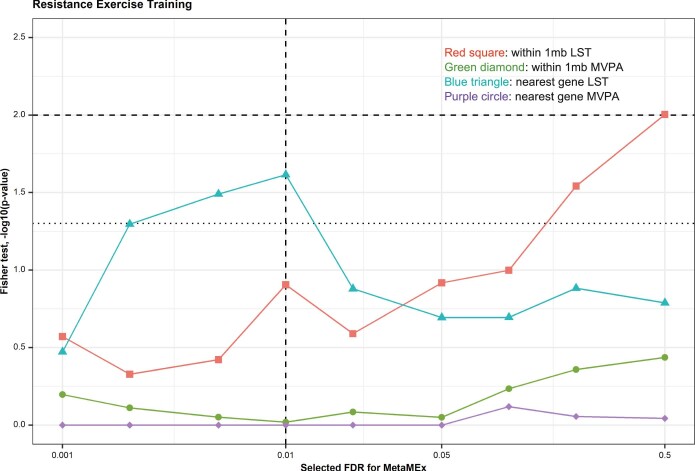
A sensitivity analysis shows the analysis of altered gene expression following resistance training is robust to FDR threshold. We examined the effect of different FDR thresholds on Fisher’s exact test results for the enrichment analysis of alteration in gene expression in skeletal muscle following resistance training. Red square, genes within 1 Mb of the LST lead SNP; green circle, genes within 1 Mb of the MVPA lead SNP; blue triangle, nearest gene LST lead SNP; purple diamond, nearest gene MVPA lead SNP. The horizonal dotted line indicates nominal significance level (*P* < 0.05), and the vertical dashed line indicates the FDR threshold that was used. FDR thresholds explored range from 0.001 to 0.5.

Although behavior is mainly influenced by signals from the brain, in the case of physical activity, characteristics of skeletal muscle can play a facilitating or restricting role^32^. Therefore, we next examine whether genes in LST- and MVPA-associated loci are enriched for altered messenger RNA expression in skeletal muscle following an acute bout of exercise or a period of training or inactivity^33^ (Methods). A mild enrichment for transcripts with an altered expression in skeletal muscle after resistance training is observed for genes nearest to lead SNPs in LST-associated loci (*P* = 0.02) (Extended Data Figs. 1 and 2, and Supplementary Table 14). Of the ten genes driving the enrichment, *PDE10A* may play a critical role in regulating cyclic AMP and cyclic GMP levels in the striatum, a brain region that harbors the central reward system and is important for physical activity regulation^34^, and in regulating striatum output^35^; *ILF3* and *NECTIN2*—near *APOE*—influence the host response to viral infections^36,37^; *EXOC4* plays a role in insulin-stimulated glucose uptake in skeletal muscle^38^; and *IMMP2L* influences the transport of proteins across the inner mitochondrial membrane^39^ (Supplementary Note).

### Visual information processing and the reward system

**Extended Data Fig. 3:**
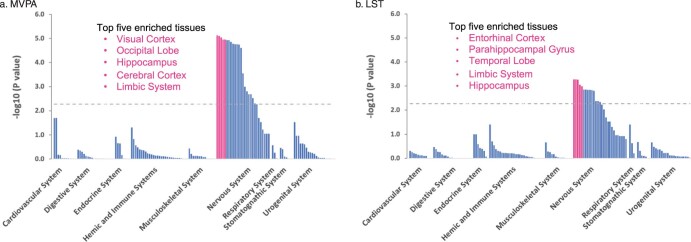
DEPICT-derived tissue enrichment of MVPA and LST. **a**, MVPA. **b**, LST. SNPs with *P* < 1 x 10^−5^ for association in the European ancestry GWAS of men and women combined were used as input. The dashed line indicates the FDR corrected significance threshold (FDR < 0.05).

To further improve the understanding of the biological factors that influence sedentary behavior and physical activity, we perform a tissue enrichment analysis using DEPICT^40^. LST- and MVPA-associated loci (*P* < 1 × 10^−5^) are most significantly enriched for genes expressed in the retina, visual cortex, occipital lobe and cerebral cortex. This suggests that: (1) possibly subtle differences in the ability to receive, integrate and process visual information influence the likelihood to engage in MVPA; (2) MVPA alters the expression of genes that play a role in visual processes in these tissues; and/or (3) MVPA can slow age-related perceptual and cognitive decline^41^. The LST-associated loci yield similar tissue enrichment results, with retina having the lowest *P* value for enrichment. Interestingly, enrichment for genes expressed in retina was also observed in the High Runner mouse model^42^. Areas related to the reward system (for example, the hippocampus and limbic system) and to memory and navigation (for example, the entorhinal cortex, parahippocampal gyrus, temporal lobe and limbic system) are also enriched in both LST- and MVPA-associated loci (Extended Data Fig. 3 and Supplementary Table 15).

**Extended Data Fig. 4:**
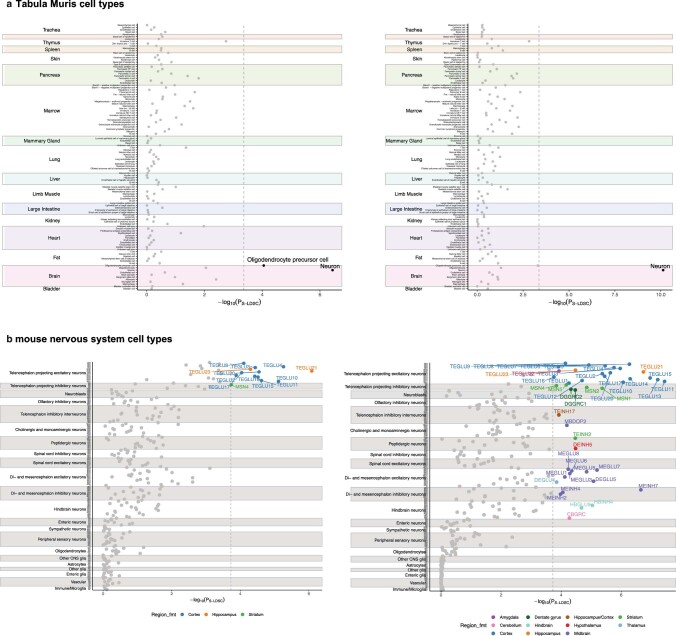
Cell type prioritization using CELLECT for MVPA and LST. **a**, Prioritization of 115 Tabula Muris cell types across 19 tissues identified two cell types from the brain as significantly associated (stratified linkage disequilibrium score regression) with MVPA (left) and LST (right), namely oligodendrocyte precursor cells and neurons (shown in black; Bonferroni-corrected significance threshold, *P* < 0.05/115). **b**, Prioritization of 265 mouse nervous system cell types identified 13 and 45 cell types from 12 distinct brain regions as significantly associated (stratified linkage disequilibrium score regression) with MVPA and LST, respectively (highlighted; Bonferroni-corrected significance threshold, *P* < 0.05/265.

We next use CELLECT^43^ to identify enriched cell types using single-cell RNA sequencing data from the Tabula Muris and mouse brain projects^44^. In Tabula Muris data, we observe enrichment in nonmyeloid neurons for MVPA and LST, and of nonmyeloid oligodendrocyte precursor cells for MVPA, possibly highlighting a role for signal transduction (Extended Data Fig. 4 and Supplementary Table 16). In mouse brain data, we identify enrichment for 13 and 45 cell types from 3 and 12 distinct brain regions for MVPA and LST, respectively, including enrichment in dopaminergic neurons (Extended Data Fig. 4 and Supplementary Table 16); a key feature of physical activity regulation in mice^45^.

### Candidate gene prioritization

**Extended Data Fig. 5:**
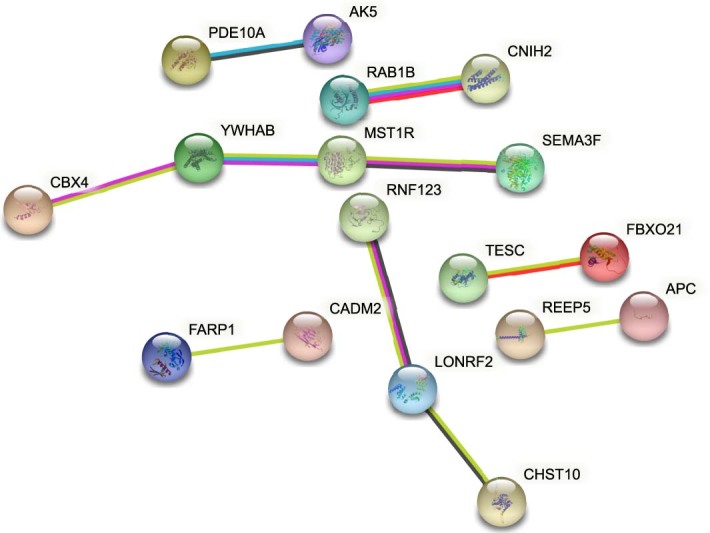
Protein-protein interactions involving 17 of the 46 candidate genes in GWAS-identified loci prioritized by at least two approaches. Protein-protein interactions were visualized using String. *LONRF2* and *CHST10* were prioritized in loci associated with MVPA; the remaining genes were prioritized in loci associated with LST.

To explore mechanisms by which the identified loci may influence LST and MVPA, we next pinpoint genes in GWAS-identified loci: (1) contributing to tissue enrichment or identified by DEPICT’s gene prioritization algorithm (Supplementary Tables 15 and 17); (2) whose expression in brain, blood and/or skeletal muscle is anticipated to mediate the association between locus and outcome based on Summary-based MR^46^ (SMR; Supplementary Table 18); (3) harboring credible variants with a high posterior probability of being causal (>0.80)^47^ and a predicted deleterious effect on protein function (Supplementary Table 19)^48^; (4) showing chromatin–chromatin interactions with credible variants in central nervous system cell types (such genes may be further from lead SNPs, Supplementary Table 19); (5) that—across 26 tissues and cell types—are activated by contact with enhancers presumably affected by causal variants flagged by GWAS hits^49^ (Supplementary Tables 20–22); (6) associated with physical activity in GWAS in humans and mice and located <100 kb from the lead variant in humans or mice (Supplementary Note, Supplementary Fig. 4 and Supplementary Tables 23 and 24); and (7) driving enrichment of altered expression in skeletal muscle following resistance exercise training (Supplementary Table 14). Twelve (14%) of the LST-associated loci harbor a variant with a high (>80%) posterior probability of being causal, whereas such variants were not identified among the 11 MVPA-associated loci (Supplementary Table 19). Integrating results across approaches yields 268 candidate genes in 70 LST-associated loci and 39 candidate genes in 8 MVPA-associated loci. Forty-six candidate genes are prioritized by multiple approaches (42 for LST and 6 for MVPA; 2 overlap) and point to endocytosis (*CNIH2*, *RAB1B*, *KLC2*, *PACS1*, *REPS1*, *DNM3, EXOC4*), locomotion (*CADM2*, *KLC2*) and myopathy (*MLF2*, *HERC1*, *KLC2*, *SIL1*) as relevant pathways (Supplementary Tables 25 and 26, and Supplementary Note). Seven clusters of protein–protein interactions are predicted, involving 17 of the 46 genes (Extended Data Fig. 5). In vivo perturbation in model systems is required to confirm or refute a role in sedentary behavior and physical activity.

### Enrichment of previously reported candidate genes

Candidate gene studies in humans have aimed to identify and characterize the role of genes in exercise (physical activity behavior) and fitness (physical activity ability) for decades. We next examine whether variants in genes that have been linked to or associated with exercise and fitness show evidence of associations with self-reported LST and MVPA^12,50–54^. Of the 58 previously described candidate genes (13 for exercise; 45 for fitness), 56 (13 for exercise and 43 for fitness) harbor variants with *P* < 0.05 for associations with LST and/or MVPA (*P*_binomial_ = 2.1 × 10^−70^; Supplementary Fig. 5 and Supplementary Table 27). Associations reach traditional genome-wide significance (*P* < 5 × 10^−8^) for variants in three genes: *APOE*^55^, *PPARD*^56^ and *ACTN3* (ref. ^57^) (Methods).

The SNP in *APOE* with the lowest *P* value for association with LST is rs429358, for which the C allele associated with lower LST was previously associated with higher self-reported MVPA^13^ and forms part of the Ɛ4 risk allele for Alzheimer’s disease (Discussion). The SNP with the lowest *P* value for association with LST in the locus is rs6857 (*D*′ = 0.90; *r*^2^ = 0.78 with rs429358), in the 3′ untranslated region of *NECTIN2*. Neither rs429358 (*P* = 0.16) nor rs6857 (*P* = 0.18) is associated with MVPA in this study.

The C allele in rs1625595, ~300 kb upstream of *ACTN3*, is associated with higher MVPA (*P* = 1.9 × 10^−11^) as well as with higher *ACTN3* expression in skeletal muscle (GTEx, *P* = 6.6 × 10^−5^). Alpha-actinin-3 (*ACTN3*) forms a structural component of the muscle’s Z-disc that is exclusively expressed in type II_A_ and II_X_ muscle fibers^58^. rs1815739, a common *ACTN3* variant that introduces a premature stop codon, p.Arg577Ter, also known as p.Arg620Ter, has been extensively studied in the context of exercise performance^57^. Although we observe little evidence for a role of rs1815739 in leisure time sedentary behavior or physical activity (*P*_LST_ = 0.017, *P*_MVPA_ = 0.17), the intronic *ACTN3* variants rs679228 (*P*_LST_ = 4.3 × 10^−8^) and rs2275998 (*P*_MVPA_ = 1.8 × 10^−7^) do show evidence of such associations. Of these, rs2275998—located 646 bp downstream of p.Arg577Ter—is in full LD (*r*^2^ = 1.0) with the missense variant rs2229456 (p.Glu635Ala), which likely affects protein function (Combined Annotation Dependent Deletion (CADD) score for the derived, minor, p.635Ala variant =28.6). Each C allele in rs2229456 is associated with less LST (*P* = 1.4 × 10^−4^) and higher odds of engaging in MVPA (*P* = 8.3 × 10^−7^). Of note, given its downstream location from p.Arg577Ter, a potentially causal effect of rs2229456 on physical activity requires absence of the protein-truncating p.Arg577Ter variant in rs1815739. Haplotype analyses support this (Supplementary Table 28).

### Greater ACTN3 flexibility with p.635Ala

**Fig. 6:**
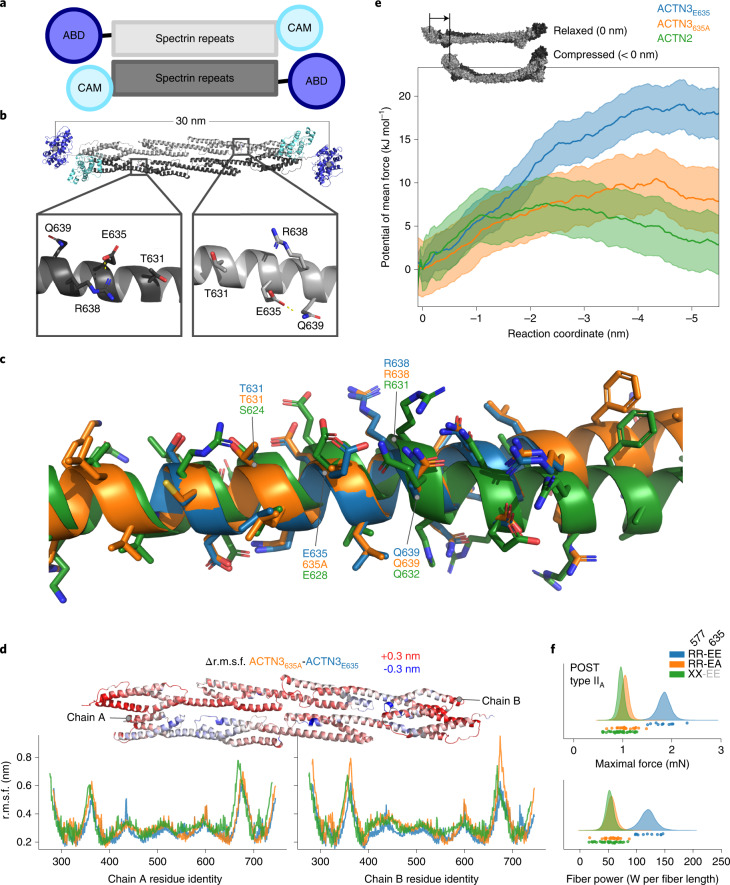
Allele p.635Ala in *ACTN3* results in a more flexible ACTN3 homodimer. **a**, ACTN3 is a homodimer of two antiparallel filaments, with each filament consisting of an N-terminal actin binding domain (ABD, blue), followed by a structural region comprised of four spectrin repeats (gray) with a C-terminal calmodulin (CAM) homology domain (cyan). **b**, The glutamate residue side chain in position 635 of ACTN3 (p.Glu635) interacts primarily with the arginine in position 638 and the glutamine in position 639. **c**, The α-helix comprised of residues adjacent to ACTN3 residue 635 (ACTN2 628) exhibits a pronounced kink in ACTN2 (green) at this α-helical turn compared with ACTN3 p.Glu635 (blue) and p.635Ala (orange), decreasing the likelihood of interactions under load with R631, whereas the alanine substitution of ACTN3 p.635Ala precludes any side chain interactions with neighboring residues p.Arg638 or p.Glu639. **d**, The r.m.s.f. of the spectrin repeat structural region of the ACTN3 dimer for a 150 ns MD simulation for variants p.Glu635 (blue) and p.635Ala (orange, higher MVPA) and ACTN2 (green) (bottom), with the difference in r.m.s.f. between ACTN3 variants shown mapped to the spectrin repeat region (top) with ±0.3 nm difference (red, positive and blue, negative). **e**, Umbrella sampling of ACTN3 variants p.Glu635 and p.635Ala and ACTN2 with orange, blue and green traces representing the potential of mean force for ACTN3 variants p.635Ala (orange) and p.Glu635 (blue) and ACTN2 (green) ±1 s.d. The reaction coordinate is the distance between the two ABD centers of mass of each dimer, a negative value indicating a shorter distance between the two ABDs. Inset shows the relaxed dimer at reaction coordinate of 0 nm (top) and the direction and effect on the compressive force. **f**, Single fiber experiments show a higher maximal force and fiber power during isotonic contractions after an eccentric exercise bout in type II_A_ fibers from an individual homozygous for p.Arg577 and p.Glu635 (blue) compared with type II_A_ fibers from three p.Arg577 homozygous, p.Glu635Ala heterozygous individuals (orange); and from four p.577Ter homozygous individuals (green).

Given the striking finding that MVPA and LST are associated with the ACTN3 missense variant rs2229456, but not with the ACTN3-truncating variant rs1815739, we next examine whether rs2229456 (p.Glu635Ala variant) has functional consequences for ACTN3’s mechanistic properties at the molecular level. We add ACTN2 to this comparison because it likely compensates for the loss of ACTN3 in the presence of the truncating p.Arg577Ter variant^59^. The results of computer-based (steered) molecular dynamics (MD) simulations and umbrella sampling (see Methods and Supplementary Note for more details) show that the ancestral p.Glu635 variant facilitates salt-bridge and hydrogen-bonding interactions at residue 635 with surrounding residues (for example, R638 and Q639; Fig. 6a,b and Supplementary Fig. 6) via its glutamate side chain. Such interactions are not formed in the presence of the ACTN3 p.635Ala product. They are also less likely to be formed in ACTN2, because of a kink that is present at exactly this location in ACTN2 (Fig. 6c and Supplementary Fig. 6). Moreover, p.635Ala and ACTN2 show distinctly different behavior from p.Glu635, with a greater magnitude of root mean squared fluctuations (r.m.s.f.) in the middle section of the spectrin repeats under no-load conditions (Fig. 6d), suggesting a more flexible structural region. When placed under simulated compressive loads that are likely experienced in vivo, p.635Ala shows a more linear force versus distance relationship, with greater variance in the potential of mean force (Fig. 6e and Supplementary Fig. 6). Taken together, these results indicate that the ACTN3 p.635Ala dimer—associated with higher MVPA—exhibits similar flexibility to ACTN2 and greater flexibility than the p.Glu635 dimer.

### Maximal force and fiber power lower with ACTN3 p.635Ala

We next examine whether a higher predicted ACTN3 dimer flexibility in the presence of p.635Ala has functional consequences in isolated human skeletal muscle fibers. To this end, we compare functional readouts in 298 isolated type I and II_A_ fibers from vastus lateralis biopsies obtained from eight healthy, young, untrained male participants before and after an eccentric exercise bout^60,61^. Results from a 15,000 iteration Markov chain Monte Carlo model show that stable maximal force—with fibers submerged in activating solution—and fiber power during isotonic load clamps are similar in 32 ± 7 fibers (mean ± s.d.) from three p.Arg577 homozygous, p.Glu635Ala heterozygous individuals compared with 39 ± 6 fibers from four individuals homozygous for the p.577Ter variant; and lower in both groups when compared with 46 fibers from an individual that is homozygous for both the p.Arg577 and p.Glu635 variants (Fig. 6f and Methods). Associations are most striking after an eccentric exercise intervention and are, as expected, more pronounced in type II_A_ than in type I fibers (Supplementary Fig. 7). Taken together, these results suggest that a more flexible ACTN dimer with lower peak performance (ACTN3 p.635Ala or ACTN2) may be less susceptible to exercise-induced muscle damage than the ancestral ACTN3 p.Glu635, thereby facilitating a more active lifestyle.

## Discussion

By doubling the sample size compared with earlier GWAS, we identify 104 independent association signals in 99 loci, including 42 newly identified loci, for self-reported traits reflecting MVPA and sedentary behavior during leisure time. Around half of these also show evidence of directionally consistent associations with objectively assessed physical activity traits. Genetic correlations and two-sample MR analyses show that lower LST results in lower adiposity. Protective causal effects of higher MVPA and lower LST—acting through or confounded by BMI—are observed for longevity. Tissue and cell-type enrichment analyses suggest a role for visual information processing and the reward system in MVPA and LST, including enrichment for dopaminergic neurons. Loci associated with LST are enriched for genes whose expression in skeletal muscle is altered by resistance training. Forty-six candidate genes are prioritized by more than one approach and point to pathways related to endocytosis, locomotion and myopathy. Finally, results from MD simulations, umbrella sampling and single fiber experiments suggest that a missense variant (rs2229456 encoding ACTN3 p.Glu635Ala) likely increases MVPA, at least in part by reducing susceptibility to exercise-induced muscle damage.

Recent MR studies reported causal protective effects of self-reported and objectively assessed physical activity on breast and colorectal cancer^62,63^. One study concluded that a 1 s.d. increase in self-reported MVPA was associated with lower odds of colorectal cancer (OR = 0.56), with BMI only mediating 2% of the protective effect^63^. Our results—on lung cancer rather than colorectal cancer—show that instrumental variables of MVPA in multivariable MR are weak, and results should be interpreted with caution. Furthermore, a causal effect of objectively assessed, but not self-reported physical activity (MVPA) on depression has been reported^64^. Our MR results for LST on depression show that although the physical activity trait matters, the self-reported nature of it seems inconsequential. According to an earlier study, TV viewing has an attenuated effect but still causes coronary artery disease when adjusting for BMI^15^. The discrepancy with our results—suggesting mediation or confounding by BMI—highlights the importance of including physical activity, as well as BMI-associated variants in multivariable MR analysis, to prevent loss of precision and potentially even biased estimates^31^.

It is of interest that a proxy of rs429358, part of the established *APOE* Ɛ4 risk allele for Alzheimer’s disease, is associated with lower LST. Klimentidis et al. previously showed that the association of rs429358 with MVPA was stronger in those reporting a family history of Alzheimer’s disease, and among older individuals^13^. Based on the direction of the association, it was hypothesized that individuals at higher risk of developing Alzheimer’s disease may adopt a healthy lifestyle to mitigate their risk, especially later in life^13^. However, our MR analyses show no evidence of a causal role of MVPA or LST in Alzheimer’s disease, and lower average physical activity levels in individuals with a first-degree family history of Alzheimer’s disease or dementia^13^ suggest other explanations are more likely, although a role for survival bias cannot be ruled out^13^. For example, *APOE* Ɛ4 carriers have a greater increase in aerobic capacity following exercise training^65^, which may reinforce a physically active lifestyle independently of Alzheimer’s risk. Furthermore, several studies have investigated the moderating role of the *APOE* Ɛ4 allele in the relationship between physical activity and Alzheimer prevention^66^. Although more studies are needed to resolve inconsistencies in the literature, Ɛ4 carriers seem to benefit more from physical activity in terms of reducing the risk of dementia and brain pathology^66^.

To investigate the molecular basis for the association of *ACTN3* with MVPA, we compare the ACTN3 p.Glu635 and p.635Ala variants (rs2229456) with each other and with ACTN2—as a functional proxy for ACTN3 p.577Ter—using MD simulations and single fiber experiments. Previous studies using normal mode analysis of alpha-actinin show that several of the natural frequencies have bending flexibility near residue 635. This is interesting because ACTN3’s residue 635—the 356^th^ residue of the spectrin repeat region (Fig. 6)—lies outside the linkers between the α-helices of the spectrin repeats, where most flexibility is expected and observed^67^. The absence of salt-bridge and hydrogen-bonding interactions between position 635 (628 in ACTN2) and surrounding residues—due to either the presence of the alanine substitution at ACTN3’s residue 635, or a kink in the α-helix at ACTN2’s residue 628—increases the flexibility of the dimer under a compressive load, with far less work required to deform the homodimer beyond a compressive distance of 1.2 nm. The p.635Ala substitution may reduce the stiffness of the muscle fiber while undergoing elastic deformation during exercise to a level that is comparable with ACTN2. Although at the expense of the maximal force that single fibers can generate, this may reduce exercise-induced microtrauma caused by Z-disc rupture or streaming^1^, alleviating delayed onset muscle soreness^2^ and risk of injuries^3^, enabling a more active lifestyle. Our results suggest it would be interesting to revisit the plethora of data on p.Arg577Ter, and differentiate between effects of the p.Arg577Ter and p.Glu635Ala variants.

In conclusion, our results shed light on genetic variants and molecular mechanisms that influence physical activity and sedentary behavior in daily life. As would be expected for complex behaviors that involve both motivation and physical ability, these mechanisms occur in multiple organs and organ systems. In addition, our causal inference supports the important public health message that a physically active lifestyle mitigates the risk of multiple diseases, in major part through or confounded by an effect on BMI.

## Methods

Each study (Supplementary Table 2) obtained informed consent from participants and approval from the appropriate institutional review boards or committees.

### Samples and study design

We conducted a large meta-analysis for physical activity traits, including results from up to 703,901 individuals (including nearly half-a-million from the UK Biobank) to identify genetic loci associated with physical activity and sedentary behavior across different ancestries. We first examined genome-wide, ancestry- and sex-stratified associations in 51 studies with questionnaire-based data on: (1) MVPA; (2) LST; (3) sedentary commuting behavior; and/or (4) sedentary behavior at work, using study-specific, tailored analysis plans (Supplementary Table 2, see Supplementary Note for rationale). Next, we performed ancestry-specific, inverse variance-weighted fixed-effects meta-analyses of summary statistics for each of the four self-reported traits (Fig. 1a), including data from up to 703,901 individuals consisting of European (94.0%), African (2.1%), East Asian (0.8%) and South Asian (1.3%) ancestries; as well as Hispanics (1.9%) (Supplementary Table 1). Our primary meta-analyses were restricted to 661,399 European ancestry participants. Secondary meta-analyses were also conducted for: (1) all ancestries (European + other ancestries), (2) European ancestry men, (3) European ancestry women, and (4) each non-European ancestry separately. Details of participating studies are described in Supplementary Tables 1 and 2. Although modest genomic inflation^68^ was observed (lambda 1.2–1.4) (Supplementary Fig. 1), LD score regression analyses indicated this reflects true polygenic architecture rather than cryptic population structure^69^.

### Self-reported physical activity and sedentary behavior traits

The self-reported outcomes in this study are domain- and intensity-specific physical activity and sedentary traits that, unlike accelerometry-based outcomes, are subject to misclassification and bias by recall and awareness of the beneficial effects of physical activity, among others. Furthermore, different studies used different questionnaires to capture physical activity, and so we defined cohort-specific traits that make optimal use of the available data, while striving for consistency across studies (Supplementary Table 2). As a result, and based on the zero-inflated negative binomial nature of the distribution of MVPA in most studies, we had to analyze MVPA as a dichotomous outcome, which had a negative impact on statistical power. Descriptive information of these four outcomes is reported by study in Supplementary Table 1.

### Genotyping, imputation and quality control

Detailed information about the genotyping platform used, and quality control measures applied within each study are presented in Supplementary Table 2. Quality control following study level analyses was conducted using standard procedures^70^.

### GWAS and meta-analyses

GWAS were performed within each study in a sex- and ancestry-specific manner. Additive genetic models accounting for family relatedness (where appropriate) were adjusted for age, age-squared, principal components reflecting population structure and additional study-specific covariates as presented in Supplementary Table 2. Analyses were limited to genotyped and imputed variants with minor allele frequency >0.1% in UK Biobank, and minor allele count >3 in other studies. Study-, sex- and ancestry-specific GWAS results were meta-analyzed using the fixed-effects, inverse variance-weighted method implemented in METAL^71^, for 19.1 to 22.5 million SNPs per trait. Because we did not include a replication stage and given the high SNP density, we applied a stricter than usual Bonferroni correction and considered associations with *P* < 5 × 10^−9^ statistically significant^72^.

To identify genome-wide significant loci, we defined a distance criterion of ±1 Mb surrounding each genome-wide significant peak (*P* < 5 × 10^−9^). We extracted previously reported genome-wide significant associations within 1 Mb of any index variants we identified from the NHGRI-EBI GWAS Catalog^11^ and PhenoScanner V2 (ref. ^73^). A locus is considered previously reported if any variant we extracted at that locus was in LD (*r*^2^ > 0.1) with a lead variant that has been associated with objectively assessed or self-reported physical activity and sedentary traits previously. To identify physical activity- and sedentary behavior-associated loci that were previously associated with obesity-related traits, we performed a look up for each lead variant (and their proxies with LD *r*^2^ > 0.2) in the GWAS catalog and PhenoScanner V2.

### SNP-based heritability estimation

To estimate the heritability explained by genotyped SNPs for each physical activity and sedentary trait, we used BOLT-REML variance components analysis^74^, a Monte Carlo average information restricted maximum likelihood algorithm implemented in the BOLT-LMM v.2.3.3 software. As in most GWAS for complex traits, the SNP heritability (up to 16%) was lower than the heritability estimates from twin studies (31%–71%)^8,9^, likely at least in part due to the absence of rare variants in GWAS^75^.

Although we performed a multi-ancestry meta-analysis, data from relatively few individuals of non-European ancestries were available to us, and our functional follow-up analyses were conducted based on the European ancestry results. Studies with data from more individuals of non-European ancestry will no doubt further increase the understanding of physical activity etiology.

### Joint and conditional analyses

To identify additional independent signals in associated loci, we performed approximate joint and conditional SNP association analyses in each locus, using GCTA^76^. Any lead SNPs identified in known long-range high-LD regions^77^ were treated as a single large locus in the GCTA analysis. We used unrelated European ancestry participants from the UK Biobank as the reference sample to acquire conditional *P* values for association.

### MTAG

MTAG results were calculated using the European ancestry meta-analysis results of LST and MVPA, using standard settings^17^. Because MTAG's estimates are biased away from zero when SNPs are null for one trait but non-null for other traits, we applied it to only the two outcomes that were most strongly genetically correlated: MVPA and LST (absolute value of genetic correlation 0.49).

### PheWAS with physical activity PGSs

To assess the out-of-sample predictive power of the variants associated with self-reported sedentary behavior and physical activity, we constructed two PGSs—for LST and for MVPA—in up to 23,723 Mount Sinai Bio*Me* BioBank participants, using summary statistics of the primary European ancestry meta-analyses and PRSice software^78^. We subsequently assessed the association of MVPA and BMI with the PGSs in individuals of European and African ancestry, as well as in Hispanic participants, within the Bio*Me* BioBank. Among the 2,765 European ancestry individuals with physical activity measurements and genotypes, the PGSs were calculated on common variants (minor allele frequency >1%) using *P* value thresholds from 5 × 10^−8^ to 1 (all variants) in the LST and MVPA GWAS, and clumping parameters of *r*^2^ < 0.5 over a 250-kb window. Logistic regression models were used to examine the associations between MVPA (defined as at least 30 min per week of MVPA yes/no in Bio*Me*) and the PGSs in European ancestry participants of Bio*Me*. In each analysis, we estimated the variance in MVPA explained by the PGS, adjusting for age, sex and the top ten principal components for population structure. For both LST and MVPA, the *P* value threshold resulting in the best performing PGS was defined based on the highest *R*^2^ increase upon adding the PGS to the regression model. To examine the generalizability of the two PGSs, we next examined their associations with MVPA in 3,206 Hispanic individuals and 2,224 African ancestry participants of Bio*Me*. We then tested each PGS for classification performance and examined whether the generated PGS was associated with any other trait by performing a PheWAS. Briefly, International Classification of Diseases 9 and 10 codes from electronic health records were mapped to phecodes using the PheWAS package^79^. Among 8,959 Bio*Me* European ancestry participants, the 1,039 disease outcomes with at least ten cases were analyzed. We used logistic regression to separately model each phecode as a function of the two PGSs, adjusting for age, age-squared, sex and the top ten principal components. Interpretation of results was restricted to outcomes with more than ten cases. Multiple testing thresholds for statistical significance were set to *P* < 4.8 × 10^−5^ (0.05/1,039).

### Genetic correlations

To explore a possibly shared genetic architecture, we next estimated genetic correlations of the four self-reported traits examined in this study and five accelerometry-assessed physical activity traits assessed in UK Biobank^14^ with relevant complex traits and diseases based on established associations at the trait level using LD score regression implemented in the LD-Hub web resource^18^. To define significance, we applied a Bonferroni correction for the 108 selected phenotypes available on LD-Hub (*P* < 4.6 × 10^−4^). Supplementary Table 10 shows the complete set of pairwise genetic correlations of the four self-reported physical activity traits with relevant complex traits and diseases. Next, we prioritized traits and diseases showing evidence of genetic overlap (associated with at least one of the physical activity traits). These can be divided into six categories: lifestyle traits, anthropometric traits, psychiatric diseases, other diseases (cardiometabolic diseases and cancer), biomarkers and others (Fig. 4). Using objectively assessed physical activity traits (accelerometry) instead of self-reported traits yielded similar results (Supplementary Fig. 2).

### Two-sample MR

We performed MR analyses to disentangle the causality between LST and MVPA, on the one hand, and BMI, on the other hand. We further investigated the causal effects of LST and MVPA on common diseases and risk factors, while considering BMI through multivariable MR. For multivariable MR, we used BMI (exposure 2) summary statistics based on UK Biobank data, and summary statistics for disease outcomes and other relevant traits based on data from the largest publicly available GWAS without data from UK Biobank participants on the MR-Base platform and OpenGWAS database^80,81^. This way, we aimed to minimize bias due to sample overlap in the two-sample MR analysis^82^. The source of each of the instruments is presented in Supplementary Table 12. Genetic instrumental variables for each of the traits and diseases consisted of genome-wide significant (*P* < 5 × 10^−8^) index SNPs. Index SNPs were LD clumped (*r*^2^ > 0.001 within a 10-Mb window) to remove any correlated variants. In the multivariable MR that evaluates the independent effects of each risk factor, the genetic instrumental variables from two risk factors were combined. For both LST and MVPA, independent loci associated with physical activity or BMI were used as instrumental variables.

We followed several steps to evaluate potential causality. Because MR results can be severely biased if instrumental SNPs show horizontal pleiotropy and violate the instrumental variable assumptions^28^, we prioritized methods that are robust to horizontal pleiotropy when calculating causal estimates. We did not use the MR-Egger intercept test to identify the presence of potential pleiotropy, because the MR-Egger intercept parameter estimate is positively biased when the NO Measurement Error assumption is violated, as indicated by lower values of *I*^2^_*GX*_ in our two-sample MR setting^83^. Instead, we applied MR-PRESSO (pleiotropy residual sum and outlier)^27^, which removes pleiotropy by identifying and discarding influential outlier predictors from the standard inverse variance-weighted test^28^. For analyses with evidence of no distortion due to pleiotropy (MR-PRESSO Global test *P* > 0.05), we considered other robust methods, for instance fixed- and random-effect inverse variance-weighted, weighted- or simple- median and mode methods. We also conducted Steiger filtering to remove variants likely influenced by reverse causation and used Cook’s distance filtering to remove outlying heterogeneous variants as deemed necessary. To select the most appropriate approach, we implemented a machine learning framework^30^. Finally, we performed a leave-one-out analysis to identify potential outliers among the variants included in the instrumental variables tested. We set the multiple testing significance threshold for MR analyses with disease outcomes at 1.9 × 10^−3^, that is, Bonferroni correction for 13 disease outcomes and 2 types of risk factors: physical activity or sedentary behavior and adiposity (0.05/(13 × 2)).

We also applied the recently published Bayesian-based MR method CAUSE, which accounts for both correlated and uncorrelated pleiotropy^26^, in evaluating bidirectional causal effects between physical activity and adiposity. Compared with the other two-sample MR methods, CAUSE calculates the posterior probabilities of the causal effect and the shared effect, and tests whether the causal model fits the data better than the sharing model. That is, it examines whether the association between the traits is more likely to be explained by causality than horizontal pleiotropy. In addition, CAUSE improves the power of MR analysis by using full genome-wide summary results (LD pruned at *r*^2^ < 0.1 with *P* < 1 × 10^−3^, as recommended by the CAUSE authors). In addition, we took advantage of the robustness of the CAUSE method—which allows overlapping GWAS samples—to test the assumption that a genetic predisposition for LST assessed later in life reflects a lifetime liability. Using the summary statistics of SNPs for childhood adiposity (comparative body size at age 10) and height (comparative height at age 10) in UK Biobank^84^, we examined bidirectional causal effects between LST and these two recalled childhood traits.

### Enrichment for genes with altered expression in skeletal muscle after an intervention

A high degree of physical fitness and a strong adaptive response to exercise interventions facilitate a physically active lifestyle. To identify plausible candidate genes in GWAS-identified loci, we examined enrichment for transcripts whose expression in skeletal muscle was changed after an acute bout of aerobic exercise, aerobic training, an acute bout of resistance exercise, resistance training and inactivity^33^. We excluded individuals with pre-existing conditions such as chronic kidney disease, chronic obstructive pulmonary disease, frailty, metabolic syndromes and obesity. We also excluded athletes because in this subgroup, transcripts with differential expression in response to (in)activity interventions are likely not representative for the general population^85^. Enrichment was examined for genes nearest to, or within 1 Mb of lead variants for LST- and MVPA-associated loci. We used false discovery rate <0.01 as the threshold for altered expression after intervention. A sensitivity analysis with a series of different false discovery rate cut-offs (0.001 to 0.5) showed that results were robust.

### Gene, tissue and cell-type prioritization

We used DEPICT^40^ to identify enriched gene sets and tissues, as well as to prioritize candidate genes in the identified loci, using variants with *P* < 1 × 10^−5^ in the primary meta-analysis of European ancestry men and women combined as input. We also used CELLECT^43^ to identify enriched cell types for physical activity, by combining MVPA and LST GWAS summary statistics with single-cell RNA sequencing data. We sought to further refine the set of prioritized candidate genes using SMR and HEIDI tests^46^. Briefly, this approach integrates summary-level data from GWAS and expression quantitative trait loci (eQTL) studies to test whether a transcript and phenotype are likely associated because of a shared causal variant (pleiotropy). We considered genes candidates if they had a Bonferroni-corrected *P*_SMR_ < 1.02 × 10^−5^ and showed no evidence of heterogeneity (*P*_HEIDI_ > 0.05), as in earlier studies^46^. Based on tissue enrichment results from DEPICT, the SMR analyses were performed using brain eQTL information obtained from GTEx-brain (*n* = 72)^86,87^, CommonMind Consortium (*n* = 467)^88^, ROSMAP (*n* = 494)^89^, and Brain-eMeta (*n* = 1,194)^87^; blood eQTL summary information obtained from the eQTLGen Consortium^90^, which is based on peripheral blood samples from 31,684 individuals; and skeletal muscle eQTL information from the GTEx project (*n* = 803)^91^.

To identify variants in GWAS-identified loci with a high posterior probability of being causal, we used LST and MVPA summary statistics as input for FINEMAP^47^. We used default parameters and selected a maximum of ten putative causal variants per locus. The output variants identified as credible were mapped to genes using tissue-specific HiC chromatin conformation capture data^92^. We integrated all HiC data in the brain (dorsolateral prefrontal cortex, hippocampus, neural progenitor cell, and adult and fetal cortex) available on FUMA v.1.3.5, using the same approach. Genes in GWAS-identified loci containing FINEMAP-identified credible coding variants with a CADD score >12.33 were also prioritized. Finally, we used data from 26 of the 131 available tissues and cell types deemed relevant for sedentary behavior and physical activity (Supplementary Table 20) to identify genes that are contacted by enhancers affected by causal variants flagged by GWAS lead SNPs, using the recently described activity-by-contact model^49^.

### Enrichment for previously reported candidate genes

We next conducted a literature review of previously reported genes with evidence of a role in exercise (physical activity behavior) and fitness (physical activity ability) and identified 58 such candidate genes (13 for exercise; 45 for fitness)^12,50–53^. For each gene, we identified all variants within the gene, examined their associations with LST and MVPA in our meta-analysis of European ancestry individuals and, for each gene–trait combination, retained the summary statistics for the variant with the lowest *P* value for association. Variants in three genes reached the traditional threshold for genome-wide significance (*PPARD*, *APOE* and *ACTN3*). Based on LD and predicted effects on protein function, rs2229456 in *ACTN3* (encoding p.Glu635Ala) may have a causal effect.

### MD simulation for p.Glu635Ala

Because no structure for human ACTN3 has yet been experimentally determined, we constructed a homology model of the p.Glu635 variant monomeric filament using the fully annotated protein (UniProt ID Q08043) using Phyre2 (ref. ^93^), with the p.635Ala variant mutated in silico. Residue 635 of ACTN3 resides in the 356th residue of the spectrin repeat region and corresponds with residue 628 in ACTN2 (see the Supplementary Methods for more information). For each variant, the spectrin repeats of the ACTN3 monomer were aligned with the crystal structure of the rod domain of alpha-actinin (PDB ID 1HCI), to give the dimeric form of ACTN3. MD system preparation and simulation was conducted with GROMACS 2020.1 (ref. ^94^) and using mdanalysis v.2.0. The MD topology was created with GROMACS pdb2gmx using the ACTN2 and ACTN3 dimer models and parameterized with the CHARMM36 all-atom force field^95^. The ACTN2 and ACTN3 dimers were placed in a rectangular simulation box with a 1.0-nm buffer between the protein and the box extent, with periodic boundary conditions in all three spatial axes. The system was solvated with TIP3P water molecules and using GROMACS genion, random solvent molecules were replaced with K^+^ and Cl^−^ to a concentration of 150 mM with additional K^+^ ions added to provide an electrostatically neutral system. Energy minimization was accomplished using the steepest descent algorithm. To equilibrate the system, two 100-ps simulations were conducted using a constant temperature ensemble (NVT, that is, a constant number of particles [N], volume [V] and temperature [T]) at 310 K via a Berendsen thermostat, followed by a constant pressure ensemble (NPT, that is, a constant number of particles [N], pressure [P] and temperature [T]) at 1 bar with a Parinello–Rahman barostat. MD simulation parameters were set in accordance with the recommendations for the CHARMM36 force field in GROMACS. A short production run of 1 ns without position restraints was followed by a full simulation of 150 ns with weak position restraints on the ABD of chain B to prevent self-interaction across the periodic boundaries.

### Steered MD and umbrella sampling for p.Glu635Ala

We next compared the properties of ACTN2 and of ACTN3 p.635Ala and p.Glu635 when placed under the simulated compressive loads that are likely experienced in vivo. The final frame of the 1-ns MD production run was used as the starting topology for steered MD simulations using fully relaxed dimers. Steered MD simulations were run for 2 ns with a pulling rate of 0.005 nm ps^−1^ and a harmonic potential of 50 kJ mol^−1^ nm^−2^. Center-of-mass pull groups were defined as the ABD of each respective monomer, with a weak position restraint placed on the Cα atom of threonine 52 (ACTN3) or threonine 45 (ACTN2)—a centrally located residue in the core of the ABD—on one ABD, enabling full rotational freedom of each ABD during the course of the steered MD simulations. The pulling vector was oriented along the axis on which the spectrin repeats were initially aligned. Suitable frames from each steered MD simulation were selected that differed by no more than 0.2 nm from 0 to −5.5 nm (a contraction of the dimer by 5.5 nm or ~18%) and were used as the starting topology for a series of 10-ns umbrella sampling simulations. Analysis of the umbrella sampling simulations was conducted using g_wham, to yield the potential of mean force versus reaction coordinate for each variant.

### Single skeletal muscle fiber functional characteristics in relation to p.Glu635Ala

Single muscle fibers from eight nonathletic young men in which contractile and morphological properties were previously characterized in vastus lateralis biopsies obtained before and after an eccentric exercise bout^60,61^ were genotyped for rs2229456. A hierarchical linear mixed effects model was constructed for each fiber type and time point using rstanarm^96^ to test the genotype fixed effect, with muscle fibers nested within each of the eight individuals as random factors for each contractile and morphological variable. Genotypes at p.Arg577Ter and p.Glu635Ala were clustered into three groups: RR-AA (*n* = 1 individual, 46 fibers, reference group); RR-AC (*n* = 3 individuals, 32 ± 5 fibers); and XX-AA (*n* = 4 individuals, 39 ± 6 fibers). Using weakly informative priors, the posterior distribution was estimated with Markov chain Monte Carlo sampling (20,000 samples total with 5,000 sample burn-in). We calculated 90% credible intervals of the posterior density and distribution-free overlapping indices^97^ to compare single fiber properties between genotypes.

### Reporting summary

Further information on research design is available in the Nature Research Reporting Summary linked to this article.

## Online content

Any methods, additional references, Nature Research reporting summaries, source data, extended data, supplementary information, acknowledgements, peer review information; details of author contributions and competing interests; and statements of data and code availability are available at 10.1038/s41588-022-01165-1.

## Supplementary information

Supplementary Information Supplementary Results, Discussion, Methods, Figs. 1–7, Box 1, study-specific acknowledgments and references.

Reporting Summary

Supplementary Tables Supplementary Tables 1–29.

## Data Availability

European and multi-ancestry meta-analyses summary statistics for the genome-wide association study are available through the NHGRI-EBI GWAS Catalog (https://www.ebi.ac.uk/gwas/downloads/summary-statistics, GCP ID: GCP000358). UK Biobank individual-level data can be obtained through a data access application available at https://www.ukbiobank.ac.uk/. In this study we made use of data made available by: MetaMex https://www.metamex.eu/; Tabula Muris https://www.czbiohub.org/tabula-muris/; Open GWAS https://gwas.mrcieu.ac.uk/; MR Base https://www.mrbase.org/; GTEx Consortium https://gtexportal.org/home/; eQTLGen Consortium https://www.eqtlgen.org/; CommonMind Consortium https://www.synapse.org/#!Synapse:syn2759792/wiki/69613; Brain zQTLServe http://mostafavilab.stat.ubc.ca/xqtl/; MetaBrain https://www.metabrain.nl/.

## Code availability

We made use of publicly available software and tools such as METAL (https://genome.sph.umich.edu/wiki/METAL), GCTA (https://yanglab.westlake.edu.cn/software/gcta/), LD score regression (https://github.com/bulik/ldsc), SMR (https://cnsgenomics.com/software/smr/) and PLINK (www.cog-genomics.org/plink/).
