# Supplementary material for: Genome-wide association analyses of physical activity and sedentary behavior provide insights into underlying mechanisms and roles in disease prevention
Source: Nat Genet. 2022 Sep 7;54(9):1332–44. doi: 10.1038/s41588-022-01165-1 (PMC9470530; doi:10.1038/s41588-022-01165-1)
Supplement: Supplementary file 1 — Supplementary Results, Discussion, Methods, Figs. 1–7, Box 1, study-specific acknowledgments and references. [file 41588_2022_1165_MOESM1_ESM.pdf]

nature portfolio

https://doi.org/10.1038/s41588-022-01165-1

Supplementary information

Genome-wide association analyses of physical activity and sedentary behavior provide insights into underlying mechanisms and roles in disease prevention

In the format provided by the authors and unedited

Genome-wide association analyses of physical activity and sedentary behavior provide insights into underlying mechanisms and roles in disease prevention

Wang et al., (the author list is provided in the main manuscript)

SUPPLEMENTARY INFORMATION

Table of Contents

SUPPLEMENTARY RESULTS 2

Physical activity loci under selection 2

Overlap between mouse and human loci 2

SUPPLEMENTARY DISCUSSION 6

SUPPLEMENTARY METHODS 8

Self-reported physical activity and sedentary behavior – rationale for trait definitions .. 8

Mouse experiments 10

Molecular dynamics simulations for the ACTN3 p.Glu635Ala variant 11

SUPPLEMENTARY FIGURES 14

SUPPLEMENTARY BOX 1 23

A Brief description of candidate genes prioritized by at least two approaches using information from Genecards, NCBI and Uniprot. 23

STUDY SPECIFIC ACKNOWLEDGEMENTS AND FUNDING 32

SUPPLEMENTARY NOTE REFERENCES 45

SUPPLEMENTARY RESULTS

Physical activity loci under selection

As much higher physical activity levels were on average required to ascertain sufficient nutrition in times of hunting and gathering and pre-mechanical farming as compared with today's Westernized societies[1], a higher capacity to be physically active may have been selected for. To explore this, we examine if MVPA and LST association signals overlap with regions identified in three genome-wide selection screens[2-4]. Here, we show that 22 genes located <100kb of lead SNPs in three MVPA and/or LST-associated loci are located in three of 412 regions under selection in the past 50,000 years[2] (**Supp Table 29**). The protein-coding genes nearest the lead SNPs (<10kb) – *DNM3*, *MST1R* and *FOXP1* – are also prioritized by other approaches (**Supp Tables 25-26**) and play a role in cell signaling and wound healing, amongst others (**Supp Box 1**). We next identify genes located <10kb of 15 loci under selection in the past 10,000 years – based on results from an ancient DNA scan[3] – and <100kb of physical activity association signals. This yields one additional gene (*GRM5*) that harbors an intronic GWAS lead SNP for LST (rs1391954, **Supp Table 29**). *GRM5* encodes a metabotropic glutamate receptor that activates phospholipase C[5]; another key player in signal transduction[6], inflammation and wound healing[7], amongst other processes. No lead SNPs for LST or MVPA are located within 1Mb of five loci under very recent selection[4]. In summary, we show that a modest number of loci (n=4) selected for in the past 10,000-50,000 years are associated with leisure time physical activity and sedentary behavior today.

Overlap between mouse and human loci

Many of the biological factors influencing sedentary behavior and physical activity are likely shared across species[8]. Identifying such loci may help prioritize candidate genes, and shed light on relevant mechanisms. To this end, we compare our findings with loci identified in a GWAS for spontaneous physical activity in 100 inbred mouse strains, performed using the Hybrid

Mouse Diversity Panel (HMDP)[9] (**Supp Table 23**). Nine genes in two LST-associated loci are also located within ±1 Mbp of two lead SNPs for distance run and average running speed in mice ($P<4.1\times10^{-6}$) (**Supp Table 24**). Of the eight genes that overlap across humans and mice in one of these two loci, *TESC* – highly expressed in the striatum – harbors the intronic lead SNP rs2173650 in humans (**Supp Box 1**). In the mouse however, a gene without an established orthologue in humans – the lncRNA 4930413E15Rik expressed in olfactory and reproductive tissues – ranks 65th out of 16,378 genes (0.4th percentile), indicating it is likely causal for high voluntary wheel running behavior in mice selectively bred for 61 generations[10]. Using single cell RNA-sequencing data from GTEx[11], we show that in humans, a sequence 1.4 Mb from rs2173650 with high conservation to the mouse 4930413E15Rik is expressed in several reproductive tissues (**Supp Figure 4**). Sex hormones are firmly established to influence physical activity regulation in animal models[12], while vomeronasal chemosensory receptors were recently proposed to play a role in voluntary exercise behavior[13]. Unfortunately, no information on gene expression in the olfactory system is available in GTEx.

Molecular dynamics simulation for p.Glu635Ala

Computer-based molecular dynamics (MD) simulations for alpha actinin 3 show that the ancestral p.Glu635 variant (A allele) product facilitates salt bridge and hydrogen bonding interactions at residue 635 with surrounding residues (e.g., p.Arg638 and p.Gln639, **Figure 6b**) via its glutamate side chain. Such interactions are not formed with the p.635Ala substitution variant and have a different pattern of interaction in ACTN2. Root mean square fluctuations were performed on the residues of spectrin repeats of each monomer chain using gmx rmsf. Root mean square fluctuations of each amino acid residue, i.e., their average displacement over the simulation compared with the starting structure, were calculated with both variants and with ACTN2, with ACTN2 as well as ACTN3 variant p.635Ala – but not ACTN3 variant p.Glu635 – showing higher fluctuations in the monomer with the restrained actin-binding domain (**Figure**

6d, Chain B, orange and green traces). Root mean square fluctuations peaks in these interface regions are around 0.8 nm for ACTN2 and the ACTN3 p.635Ala variant, while the ACTN3 p.Glu635 variant fluctuations are approximately 0.6 nm (**Figure 6d**). In the presence of p.635Ala, moderately higher root mean square fluctuations values were observed in the middle section of the spectrin repeats – over the residue range of 410-540 – though p.Glu635 showed a more pronounced peak near residue 440 (**Figure 6d**). Overall, ACTN2 and ACTN3 p.635Ala showed a similar behavior that is distinctly different from p.Glu635, with a greater magnitude of root mean square fluctuations under no-load conditions, suggesting a more flexible structural region in the presence of ACTN2 or the ACTN3 p.635Ala variant product (C allele), associated with higher MVPA.

Steered molecular dynamics and Umbrella sampling for p.Glu635Ala

When a compressive force was applied between the center of mass of the two actin-binding domains, the force required to compress the two actin-binding domains by 0.6 nm was lower for both ACTN3 p.635Ala and ACTN2 compared with ACTN3 p.Glu635 (50, 45 and 74 kJ mol^{-1} nm^{-1}, respectively). Furthermore, the force-to-distance relationship to a compressive distance of -1.2 nm – where the two respective forces converge (67 kJ mol^{-1} nm^{-1}) – was notably more linear for both ACTN3 p.635Ala and ACTN2 than for ACTN3 p.Glu635 (**Supp Figure 6**). Greater variability is also seen for ACTN2 and ACTN3 p.635Ala in the force versus distance relationship among triplicate steered molecular dynamics simulations. To explore these features further, we used umbrella sampling to examine the change in potential of mean force (free energy surface) over the reaction coordinate corresponding to the compression of the ACTN3 dimer.

Umbrella sampling of ACTN2 and the ACTN3 dimer variants showed that the initial compression of the two ACTN3 variants and ACTN2 from a relaxed state to a compression of 1.2 nm was similar, requiring energy input of approximately 4.6 kJ mol^{-1}. Beyond this distance of 1.2 nm ACTN3 p.Glu635Ala diverged from ACTN3 p.635Ala and ACTN2 in its response to

compression (**Figure 6e**). ACTN3 p.635Ala required 2.8 kJ mol^{-1} to compress the dimer from -1.2 to -2.3 nm, while ACTN3 p.Glu635 required ~6.5 kJ mol^{-1} from -1.2 to -2.3 nm, ACTN2 having reached its peak of 7.5 kJ mol^{-1} at a compression of -2.3 nm (**Figure 6e**). Interestingly, bootstrap estimation of the error of the potential of mean force showed greater variance for p.635Ala, in line with and strengthening the root mean square fluctuations and steered molecular dynamics simulations results. Taken together, these results indicate that the ACTN3 p.635Ala dimer - associated with higher MVPA – exhibits greater flexibility than the p.Glu635 dimer.

SUPPLEMENTARY DISCUSSION

Results in post-GWAS analyses were most robust for LST, as a result of a markedly larger number of loci showing associations with LST as compared with the other three outcomes. There are at least five reasons for identifying most loci for LST. First, the SNP heritability was approximately twice as high for LST as for the other outcomes. Secondly, LST is the only outcome that was recorded in a homogenous manner across all studies. For the other three outcomes, the exact questions and response options used to capture the underlying latent variable differed across studies. For those outcomes, we summarized data from all available, relevant questions in each study into the most informative outcome that was still comparable across studies. The more heterogeneous nature in which the other three outcomes were captured negatively affects the statistical power to identify associated loci. Furthermore, while LST was normally distributed and could be analyzed as a continuous outcome, the other three traits were analyzed as dichotomous outcomes, resulting in a lower statistical power. In addition, due to the negative binomial distribution of MVPA – with an inflation of zeros – we used the median of 20 mins/week as a threshold to distinguish between "active" and inactive individuals. While this threshold is statistically sound, it essentially distinguishes between individuals that partake in some MVPA vs. those that do not. This likely negatively affected the likelihood of identifying loci that are truly relevant for participation in MVPA. Finally, the sample size for LST was much larger than for sedentary commuting behavior and sedentary behavior at work.

Aiming to improve the understanding of the molecular basis of physical activity, we perform a range of largely complementary approaches to identify candidate genes through which the association signals are anticipated to act. Strikingly, of the 268 and 39 genes prioritized across 70 LST- and eight MVPA-associated loci, only 22 genes are prioritized by >1 approach when using traditional cut-offs within each approach. This illustrates the complexity of *in silico* gene prioritization for complex behaviors, especially when proof-of-concept genes are sparse and a gold standard approach for prioritization is nonexistent. When combining results

from multiple approaches, applying more lenient criteria in individual approaches is justifiable. First, because the odds that a gene with an FDR of (e.g.) 0.20 in two methodologically independent approaches represents a false positive is low (i.e. 0.04); and secondly because sensitivity is more important than specificity when using *in silico* gene prioritization results to guide the selection of genes for downstream genetic perturbation studies in high throughput model systems. Applying more lenient criteria in the individual gene prioritization approaches increased the number of genes prioritized by >1 approach from 22 to 46 (Supp Tables 25-26).

SUPPLEMENTARY METHODS

Self-reported physical activity and sedentary behavior – rationale for trait definitions

When initiating this effort in 2011 (i.e. pre-UK Biobank), we first explored what physical activity-related questions were available in the first ~20 cohort studies that agreed to participate. This served several purposes: 1) to identify common ground; 2) to explore the distributions of the available outcomes; and 3) to assess how traits could (or should) be analyzed to maximize the statistical power to identify relevant loci. Since we were interested in identifying genetic factors that are relevant for daily physical activity levels, we aimed to capture all moderate-to-vigorous intensity physical activity (MVPA) during leisure time, rather than limiting ourselves to just sports and exercise participation. Since the median time spent on all MVPA during leisure time in the first ~20 cohort studies was merely ~20 mins per week (with a zero-inflated negative binomial distribution), we decided against further dissecting this trait into MPA and VPA separately. Leisure screen time was the only outcome that we could analyze in a continuous manner, thanks to the uniform and continuous manner in which it had been acquired in all studies, and thanks to its normal distribution. For commuting behavior and physical activity at work, the distributions were such that contrasting the most active individuals with the remainder of the population would have resulted in a much lower statistical power than instead contrasting the least active individuals with the remainder of the population. Since then, it has been shown that: 1) among regular commuters, those exclusively commuting by car had an 11% higher risk of cardiovascular events, and a 30% higher risk of fatal cardiovascular events compared with individuals with a more active mode of commuting, during a mean follow-up of seven years[14]; and 2) more sitting at work is associated with higher mortality during follow-up in primary industry, i.e. non-office professions[15]. Taken together, these results suggest that identifying genetic factors associated with the outcomes as defined in our study had the potential to yield clinically meaningful and possibly actionable insights.

Besides trait definitions varying across studies, the average age per cohort ranged from early adulthood to old-age (17-74 years old). The power to detect genetic factors that influence physical activity was thus likely compromised by misclassification of physically active and inactive individuals, and heterogeneity by the inclusion of older age groups in the meta-analysis, as the heritability of physical activity decreases with increasing age[16]. However, such factors could have resulted in type II – but not type I – errors in the meta-analysis. Despite these limitations, a large sample size helped us identify 42 previously unreported loci for self-reported physical activity and sedentary behavior. Genetic correlations with objectively assessed physical activity traits were modest and five of eight previously reported loci for objectively assessed physical activity show evidence of association with self-reported physical activity and sedentary behavior. Hence, despite the well-known limitations of self-reported physical activity, focusing on domain and intensity specific physical activity traits in a large study sample helped increase the understanding of the genetic etiology of this complex behavior in a manner that is not currently possible using objectively assessed physical activity.

GWAS and meta-analyses – additional analyses

Several analyses were performed at the discovery stage for which we decided not to report associations. First, for all outcomes, we examined genome-wide associations with and without adjusting for BMI. To avoid drawing conclusions that are driven by collider bias[17], we did not use the BMI-adjusted associations. Secondly, to further explore potential linear associations with MVPA, we used zero-inflated negative binomial regression and modeled MVPA as a continuous outcome (mins/week). Associations were examined using the same covariates as in the main genome-wide analyses amongst 371,244 unrelated UK Biobank participants of European ancestry, for variants with $P<1\times10^{-5}$ for association with the dichotomous MVPA outcome in the European ancestry meta-analysis. The approach yielded similar results and are therefore not shown. Finally, gene-based analyses aggregating rare (MAF<1%) functional variants as

annotated by Ensembl's variant effect predictor (VEP)[18] were conducted in UK Biobank European ancestry participants. Gene-based Burden and SKAT tests were performed using a mixed model approach in the GENESIS package[19]. Genes identified using these approaches were also flagged by the single variant analysis and hence, the results are not shown.

Mouse experiments

Females from 100 genetically distinct strains from the Hybrid Mouse Diversity Panel (HMDP)[20] were purchased from Jackson Laboratories (University of Tennessee Health Science Center). They arrived at UCLA at 5 to 8 weeks of age and were housed 1-4 weeks until wheel testing. All mice were ~3 months old at the start of the experimental protocol, and were randomized into two groups: 1) sedentary or no exercise; and 2) exercise trained. Strains used and sample size per group are shown in **Supp Table 23**. Trained animals were housed unaccompanied on a standard 12-hour light dark cycle (6AM to 6PM local time). They were fed on a standard laboratory chow diet (8604, Teklad) with ad libitum access to food and water for the entire duration of the experiment. Mice were given full-time access to a running wheel for ~30 days. Wheel revolutions were tallied every 15 sec using VitalView® Activity Software (Starr Life Sciences Corp, Oakmont, Pennsylvania, United States). Daily running distances were calculated by converting wheel revolutions into distance using wheel circumference (35.9 cm). Average running speed was calculated by averaging all non-zero-wheel revolutions and normalizing on a per sec basis. Percent time running was calculated by dividing all 15 sec bouts a wheel revolution was recorded to the total number of intervals. Additional information on GWAS performed using the Hybrid Mouse Diversity Panel (HMDP) is described elsewhere[9]. All studies were approved by the Institutional Animal Care and Use Committee (IACUC) and the Animal Research Committee (ARC # 1992-169-83e) at the University of California, Los Angeles (UCLA).

In a selection study for high voluntary wheel-running behavior[10], the mouse lncRNA 4930413E15Rik was considered to show a strong indication of consensus in a locus that was associated with daily distance run and average voluntary running speed in a GWAS in 100 mouse strains, as well as with LST in humans. The mouse gene 4930413E15Rik is located on chr 5, spanning the coordinates 118,952,339 - 118,961,261 (mm10 assembly). To investigate the corresponding region in the human genome, a lift-over to hg19 was performed in the UCSC genome browser, resulting in the coordinates chr 12: 116,087,265 – 116,097,521. The region on chr 12 contains no established gene models, but was further investigated in the GTEx IGV browser[11] to study expression that might be present at low levels in specific human tissues.

Molecular dynamics simulations for the ACTN3 p.Glu635Ala variant

Alpha-actinin is a structural member of vertebrate muscle Z-discs, and primarily functions to cross-link neighboring actin filaments of opposite polarity from adjacent sarcomeres. This binding can occur over a range of angles from 60 to 120°, creating a tetragonal lattice with a lattice spacing of 19 to 25 nm[21-23]. In addition to its interaction with actin, alpha-actinin binds and anchors titin to the Z-disc[24]. The alpha-actinin homodimer is formed from two antiparallel subunits composed of an N-terminal actin-binding domain and a C-terminal calmodulin homology domain (CAM), separated by four spectrin-like repeats. Each repeat consists of a triple α-helix coiled-coil (**Figure 6A**). Alpha-actinin 3 (*ACTN3*) at 901 amino acids in length is one of four isoforms of alpha -actinin and is exclusively found in human type-II (also known as fast-twitch) skeletal muscle fibers. The naturally occurring truncating mutation R557X in *ACTN3* has a potential impact on injury risk during exercise, increased muscle-damage following eccentric training and increased flexibility for 557X homozygotes, who are generally presumed to have ACTN2 incorporated in their type II muscle fibers to compensate for the functional loss of ACTN3[25,26].

Steered molecular dynamics and Umbrella sampling for p.Glu635Ala

We compared the properties of ACTN2 and of the ACTN3 p.635Ala and p.Glu635 variants when placed under the simulated compressive loads that are likely experienced *in vivo*. The final frame of the 1 ns molecular dynamics production run was used as the starting topology for steered molecular dynamics simulations using fully relaxed dimers. Steered molecular dynamics simulations were run for 2 ns with a pulling rate of 0.005 nm ps^{-1} and a harmonic potential of 50 kJ mol^{-1} nm^{-2}. Centre of mass pull groups were defined as the actin-binding domain of each respective monomer, with a weak position restraint placed on the Cα atom of threonine-52 (ACTN3) or threonine-45 (ACTN2) – a centrally located residue in the core of the actin-binding domain – on one actin-binding domain, enabling full rotational freedom of each actin-binding domain during the course of the steered molecular dynamics simulations. The pulling vector was oriented along the axis on which the spectrin repeats were initially aligned. Suitable frames from each steered molecular dynamics simulation were selected that differed by no more than 0.2 nm from 0 to -5.5 nm (a contraction of the dimer by 5.5 nm or ~18%) and were used as the starting topology for a series of 10 ns umbrella sampling simulations. Analysis of the umbrella sampling simulations was conducted using g_wham, to yield the potential of mean force versus reaction coordinate for each variant.

When a compressive force was applied between the center of mass of the two actin-binding domains, the force required to compress the two actin-binding domains by 0.6 nm was lower for both ACTN3 p.635Ala and ACTN2 compared with ACTN3 p.Glu635 (50, 45 and 74 kJ mol^{-1} nm^{-1}, respectively). Furthermore, the force-to-distance relationship to a compressive distance of -1.2 nm – where the two respective forces converge (67 kJ mol^{-1} nm^{-1}) – was notably more linear for both ACTN3 p.635Ala and ACTN2 than for ACTN3 p.Glu635 (**Supp Figure 6**). Greater variability is also seen for ACTN2 and ACTN3 p.635Ala in the force versus distance relationship among triplicate steered molecular dynamics simulations. To explore these features further, we used umbrella sampling to examine the change in potential of mean force (free

energy surface) over the reaction coordinate corresponding to the compression of the ACTN3 dimer.

Umbrella sampling of ACTN2 and the ACTN3 dimer variants showed that the initial compression of the two ACTN3 variants and ACTN2 from a relaxed state to a compression of 1.2 nm was similar, requiring energy input of approximately 4.6 kJ mol^{-1}. Beyond this distance of 1.2 nm, ACTN3 p.Glu635 diverged from ACTN3 p.635Ala and ACTN2 in its response to compression (**Figure 6E**). ACTN3 p.635Ala required 2.8 kJ mol^{-1} to compress the dimer from -1.2 to -2.3 nm, while ACTN3 p.Glu635 required ~6.5 kJ mol^{-1} from -1.2 to -2.3 nm, ACTN2 having reached its peak of 7.5 kJ mol^{-1} at a compression of -2.3 nm (**Figure 6E**). Interestingly, bootstrap estimation of the error of the potential of mean force showed greater variance for p.635Ala, in line with and strengthening the root mean square fluctuations and steered molecular dynamics simulations results. Taken together, these results indicate that the ACTN3 p.635Ala dimer - associated with higher MVPA – exhibits greater flexibility than the p.Glu635 dimer.

SUPPLEMENTARY FIGURES

Supplementary Figure 1 – Quantile-quantile plots for the primary GWAS of self-reported physical activity and sedentary traits (**page 15**)

Supplementary Figure 2 – Significant genetic correlations for accelerometer-assessed physical activity with 108 other traits and diseases in 91,105 UK Biobank participants (**page 16**)

Supplementary Figure 3 – Manhattan plot of PheWAS for polygenic score of MVPA shows association with morbid obesity in European ancestry individuals in the BioMe Biobank (**page 17**)

Supplementary Figure 4 – RNA-seq data from GTEx displaying expression levels in the region chr12: 116,087,265 - 116,097,521 across several human tissues (**page 18**)

Supplementary Figure 5 – QQ plots of 28,390 variants shows enrichment for association with MVPA and LST in 56 previously reported physical activity- or exercise-related genes (**page 19**)

Supplementary Figure 6 – Steered Molecular Dynamics (SMD) and hydrogen Bond Analysis (HBA) of ACTN3 p.Glu635, ACTN3 p.635Ala and ACTN2 from a homology structure shows a divergence in behavior under compressive force (**page 20**)

Supplementary Figure 7 – Single muscle fiber experiments show a higher maximal stable force and fiber power in p.Glu635 compared with p.635Ala (**page 21-22**).

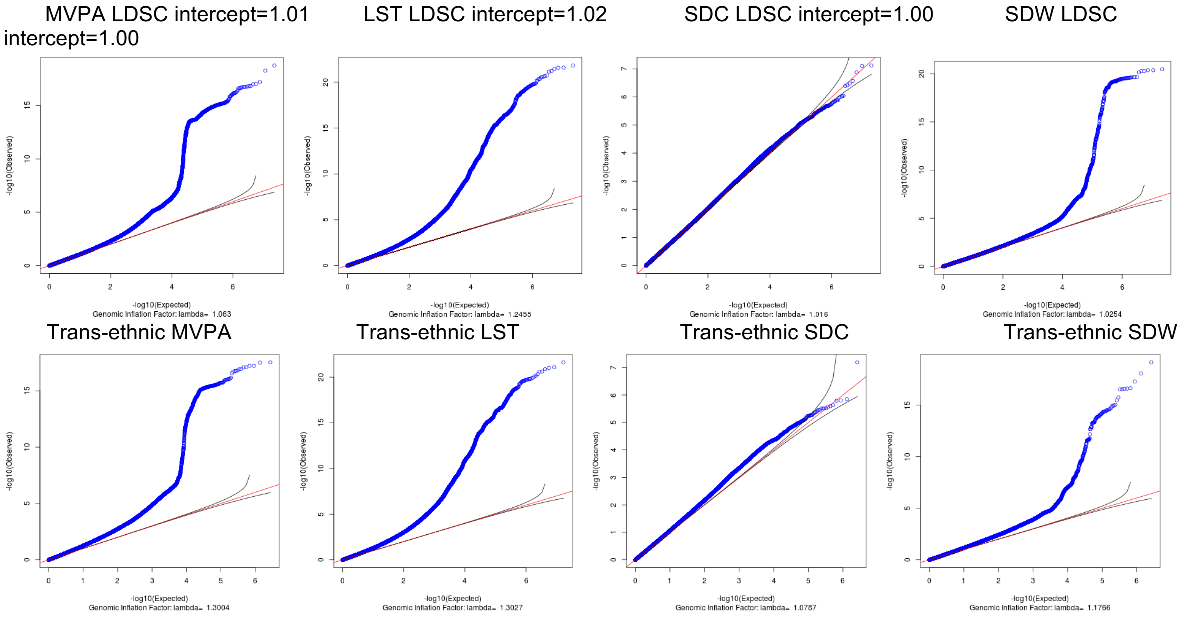

Supplementary Figure 1: **Quantile-quantile plots for the primary GWAS of self-reported physical activity and sedentary traits.** Moderate-to-vigorous intensity physical activity during leisure time (MVPA); leisure screen time (LST); sedentary behavior at work (SDW); and sedentary commuting behavior (SDC) in individuals of European ancestry only (top) as well as from the multi-ancestry meta-analysis (bottom). The estimated LD Score intercept for the primary GWAS is indicated for the former.

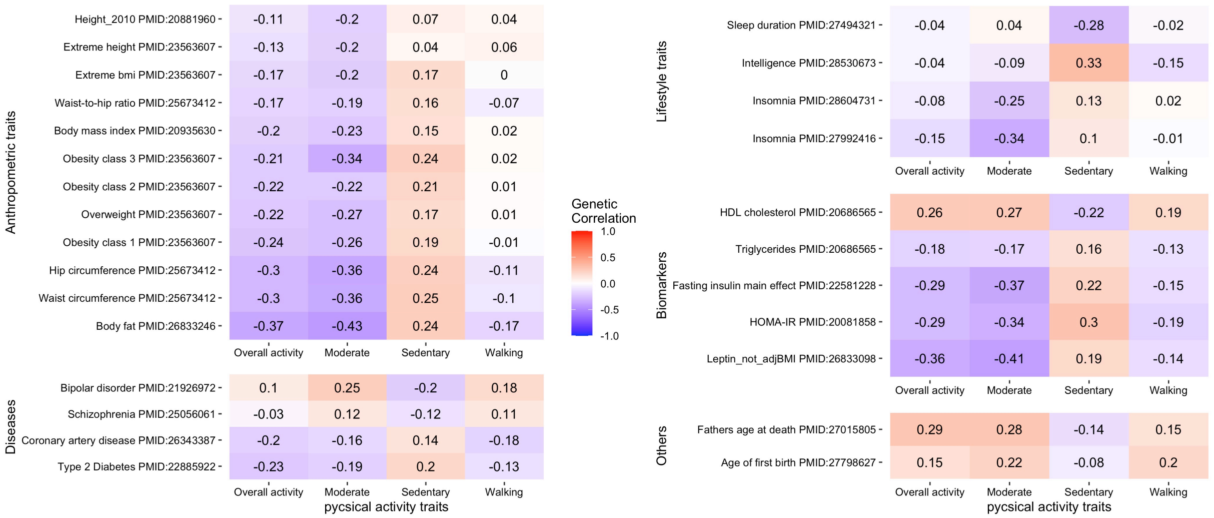

	Overall activity	Moderate	Sedentary	Walking
Height_2010 PMID:20881960	-0.11	-0.2	0.07	0.04
Extreme height PMID:23563607	-0.13	-0.2	0.04	0.06
Extreme bmi PMID:23563607	-0.17	-0.2	0.17	0
Waist-to-hip ratio PMID:25673412	-0.17	-0.19	0.16	-0.07
Body mass index PMID:20935630	-0.2	-0.23	0.15	0.02
Obesity class 3 PMID:23563607	-0.21	-0.34	0.24	0.02
Obesity class 2 PMID:23563607	-0.22	-0.22	0.21	0.01
Overweight PMID:23563607	-0.22	-0.27	0.17	0.01
Obesity class 1 PMID:23563607	-0.24	-0.26	0.19	-0.01
Hip circumference PMID:25673412	-0.3	-0.36	0.24	-0.11
Waist circumference PMID:25673412	-0.3	-0.36	0.25	-0.1
Body fat PMID:26833246	-0.37	-0.43	0.24	-0.17
Bipolar disorder PMID:21926972	0.1	0.25	-0.2	0.18
Schizophrenia PMID:25056061	-0.03	0.12	-0.12	0.11
Coronary artery disease PMID:26343387	-0.2	-0.16	0.14	-0.18
Type 2 Diabetes PMID:22885922	-0.23	-0.19	0.2	-0.13
Sleep duration PMID:27494321	-0.04	0.04	-0.28	-0.02
Intelligence PMID:28530673	-0.04	-0.09	0.33	-0.15
Insomnia PMID:28604731	-0.08	-0.25	0.13	0.02
Insomnia PMID:27992416	-0.15	-0.34	0.1	-0.01
HDL cholesterol PMID:20686565	0.26	0.27	-0.22	0.19
Triglycerides PMID:20686565	-0.18	-0.17	0.16	-0.13
Fasting insulin main effect PMID:22581228	-0.29	-0.37	0.22	-0.15
HOMA-IR PMID:20081858	-0.29	-0.34	0.3	-0.19
Leptin_not_adjBMI PMID:26833098	-0.36	-0.41	0.19	-0.14
Fathers age at death PMID:27015805	0.29	0.28	-0.14	0.15
Age of first birth PMID:27798627	0.15	0.22	-0.08	0.2

Supplementary Figure 2: **Genetic correlations of accelerometer-assessed physical activity with other traits and diseases in 91,105 UK Biobank participants**. We computed genetic correlations of four objectively assessed physical activity traits with 108 other traits and diseases using LD score regression, and show results for traits and diseases with at least one genetic correlation of P< $4.6{\times}10^{-4}$ with an objectively assessed physical activity trait.

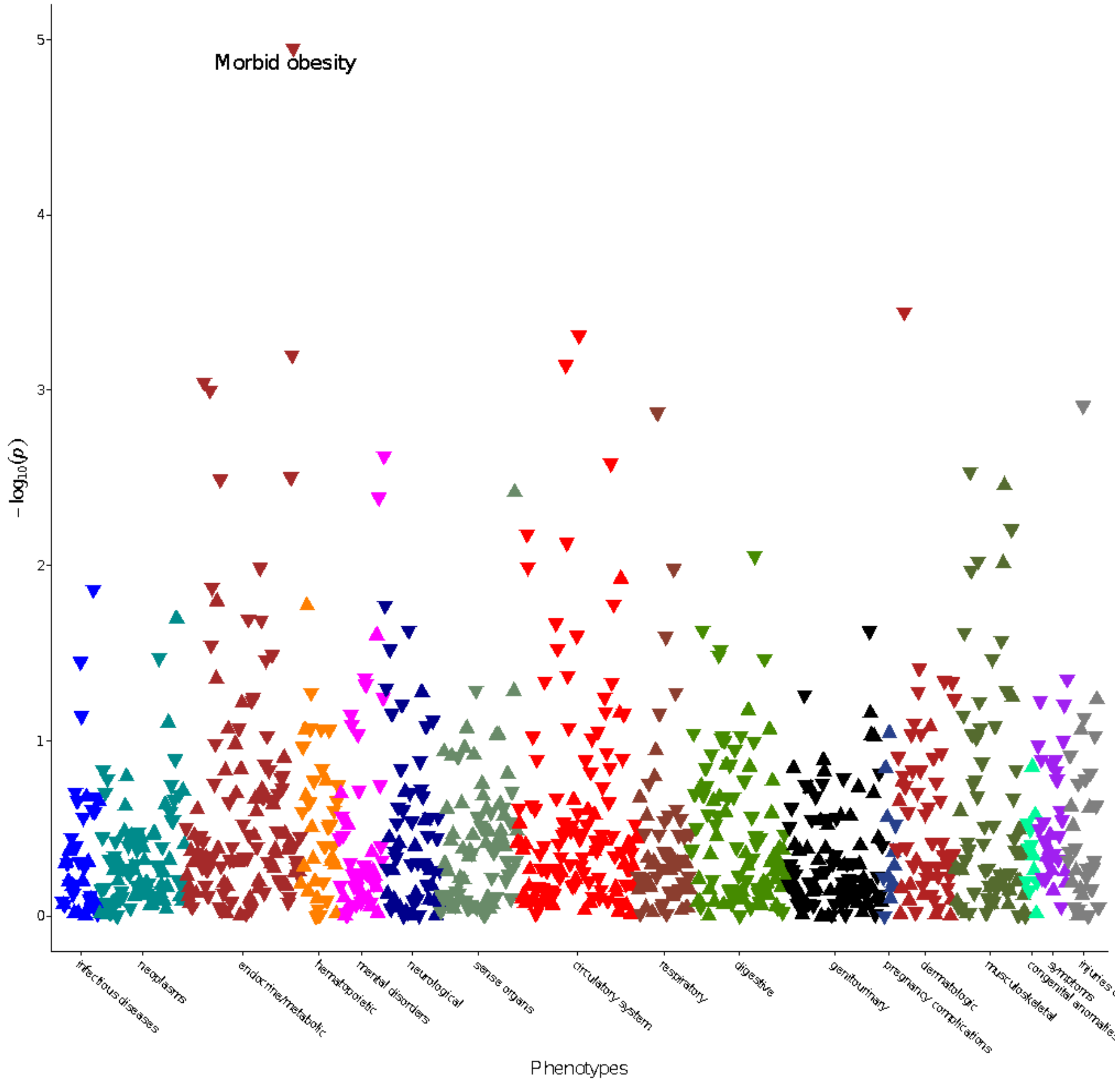

Supplementary Figure 3: Manhattan plot of PheWAS for polygenic score of MVPA shows association with morbid obesity in European ancestry individuals (N=8,959) in the BioMe Biobank. MVPA: moderate-to-vigorous intensity physical activity during leisure time, PheWAS: phenome-wide association study.

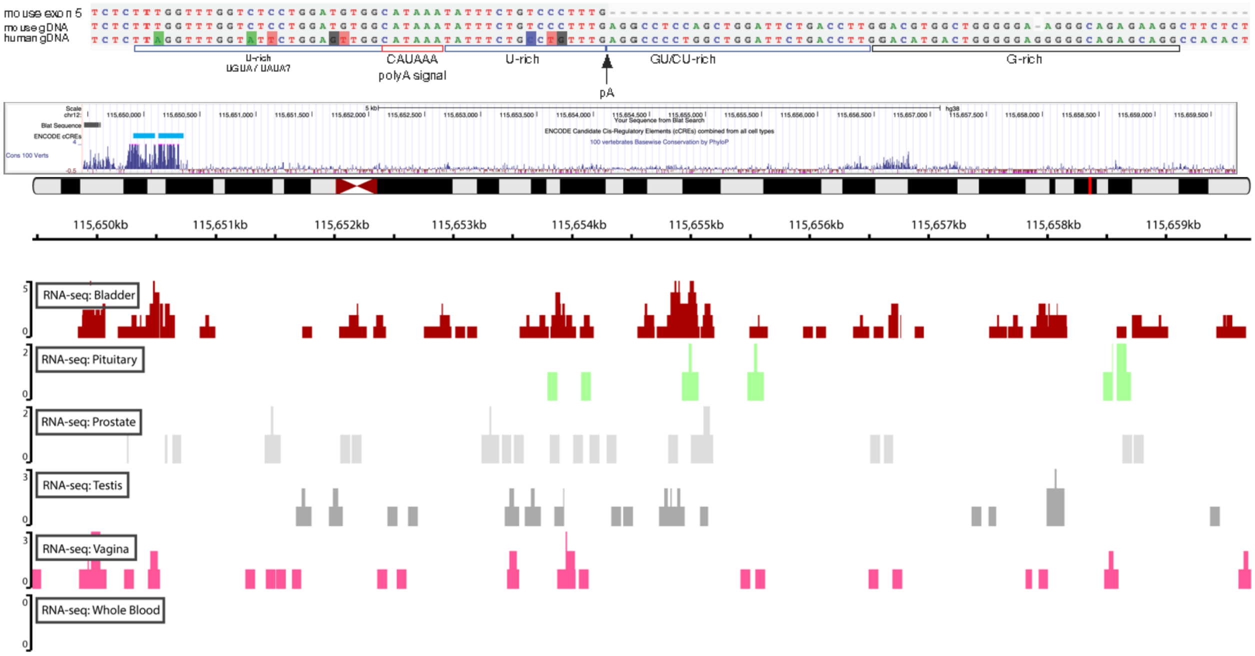

Supplementary Figure 4: RNA-seq data from GTEx displaying expression levels in the region chr12: 116,087,265 - 116,097,521 across several human tissues. Top: the region surrounding and including a poly-A signal is conserved across the mouse lncRNA 4930413E15Rik-encoding sequence and a locus on the human Chr 12 that is associated with leisure screen time; **Middle**: the human region on Chr 12 corresponding with an exonic sequence from the mouse 4930413E15Rik contains regulatory elements; **Bottom**: the human transcript is most highly expressed across the five highlighted tissues. In other tissues in the GTEx data collection, including whole blood (bottom), the region shows little or no evidence of expression. Chromosomal coordinates shown are from human genome built GRCh38.

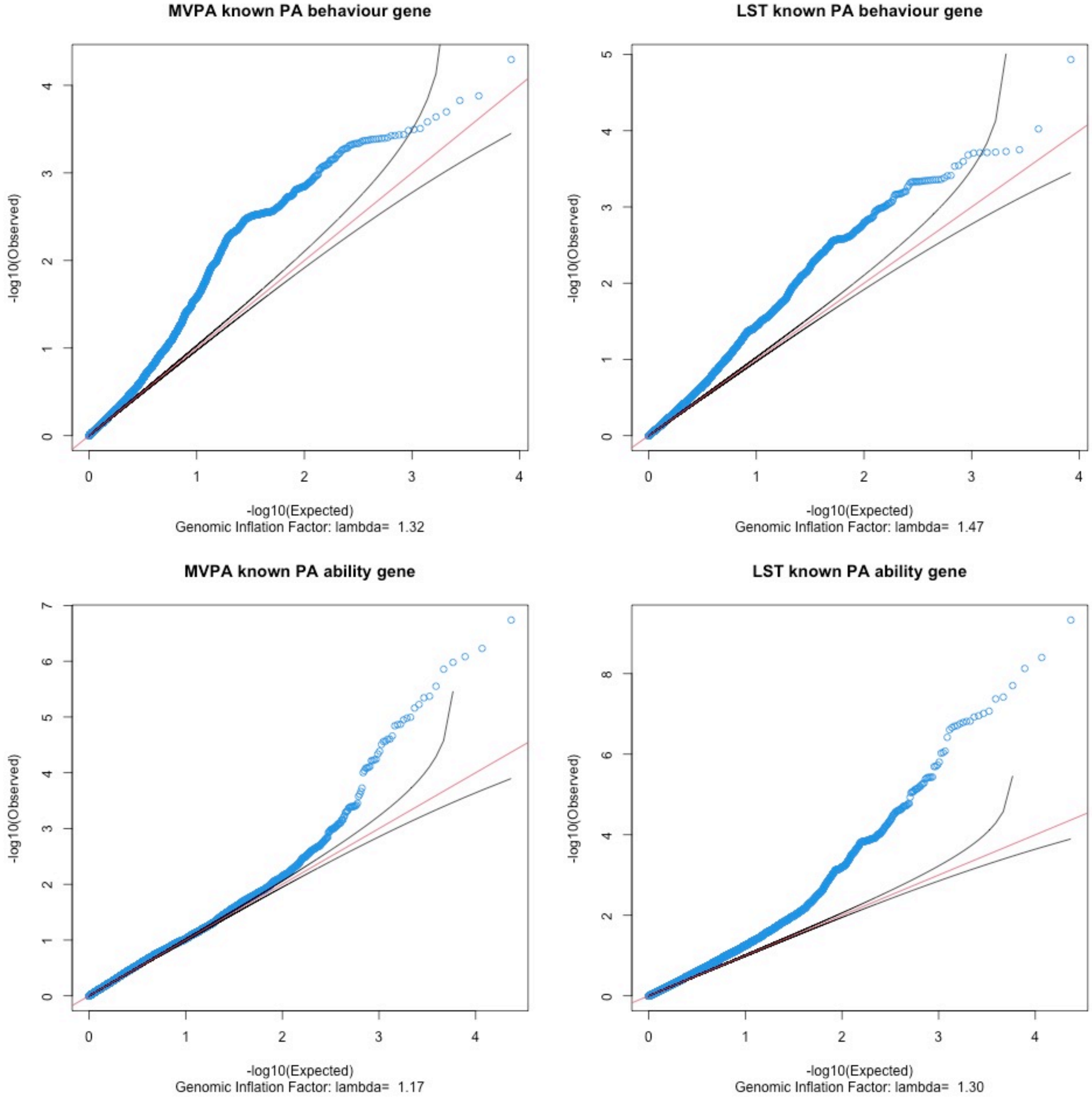

Supplementary Figure 5: QQ plots of 31,673 variants show enrichment for association with MVPA and LST in 58 previously reported exercise (i.e. physical activity (PA) behavior) and fitness (i.e. physical activity ability) genes. 8,345 variants within 13 physical activity behavior genes, 23,328 variants within 45 PA ability genes. MVPA: moderate-to-vigorous intensity physical activity during leisure time; LST: leisure screen time.

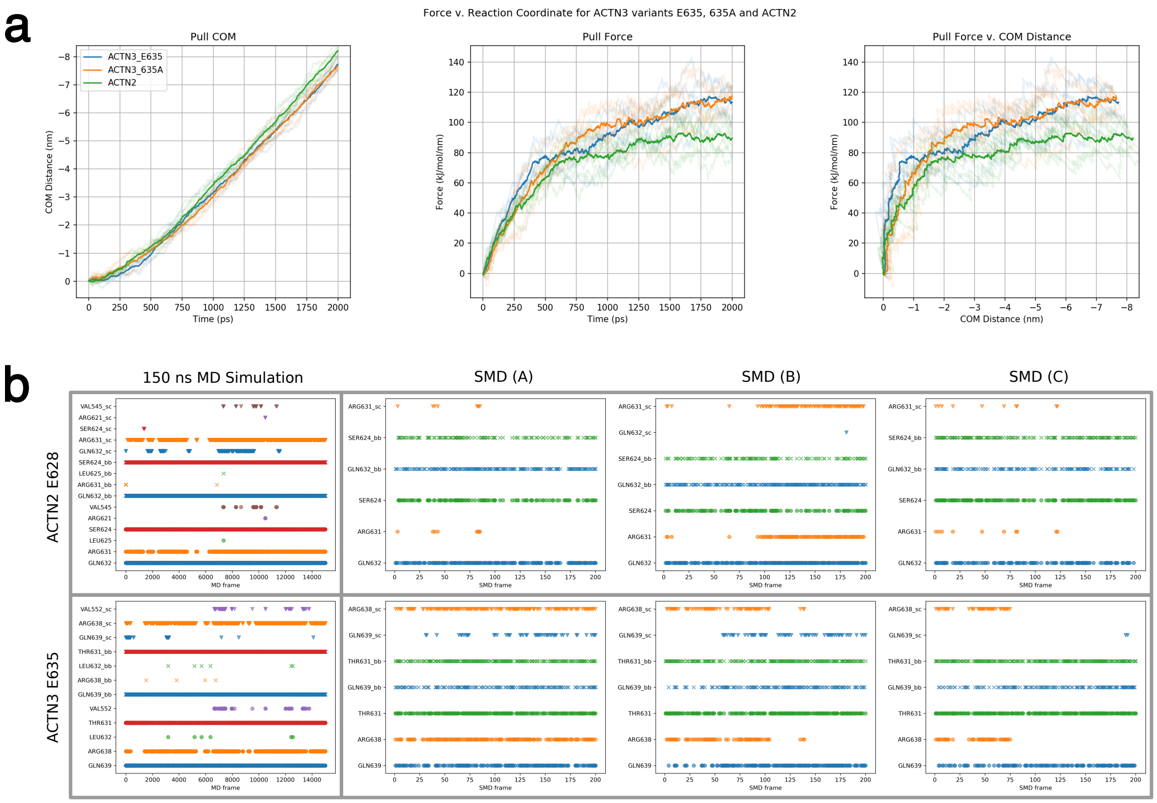

Supplementary Figure 6. Steered Molecular Dynamics (SMD) and Hydrogen Bond Analysis (HBA) of ACTN3 p.Glu635, ACTN3 p.635Ala and ACTN2 from a homology structure shows a divergence in behavior under compressive force. a) SMD (replicates shown in transparency) of ACTN3 p.Glu635 (blue, 3 replicates), ACTN3 p.635Ala (orange, 3 replicates) and ACTN2 (green, 6 replicates), showing, left to right: the pulling center-of-mass (COM) distance between actin binding domains versus time; the pull force versus time; and the pull force versus pulling COM. **b)** HBA of interactions between the glutamate of: ACTN2 residue p.Glu628 (top row); ACTN3 residue p.Glu635 (bottom row) and neighboring residues within the relaxed 150 ns MD simulation (left column) and three SMD replicates (rightmost three columns). Interacting residues are indicated on the y-axis with the suffix '_sc' denoting side chain interactions, '_bb' backbone interactions and without suffix are total interactions. ACTN2 p.Glu628's side chain interacts with p.Arg631 in the relaxed dimer (top left), but this interaction tends to break upon application of force in SMD simulations (top right). ACTN3 p.Glu635's side chain interacts with p.Arg638 in the relaxed dimer and to a lesser extent with p.Gln639 (bottom left), though as increased force is applied in SMD simulations, the interaction with p.Arg638 is generally sustained for a longer period than that seen in ACTN2, and the interaction with p.Gln639 tends to become more extensive under compression, which is not seen in ACTN2 (and p.Gln632).

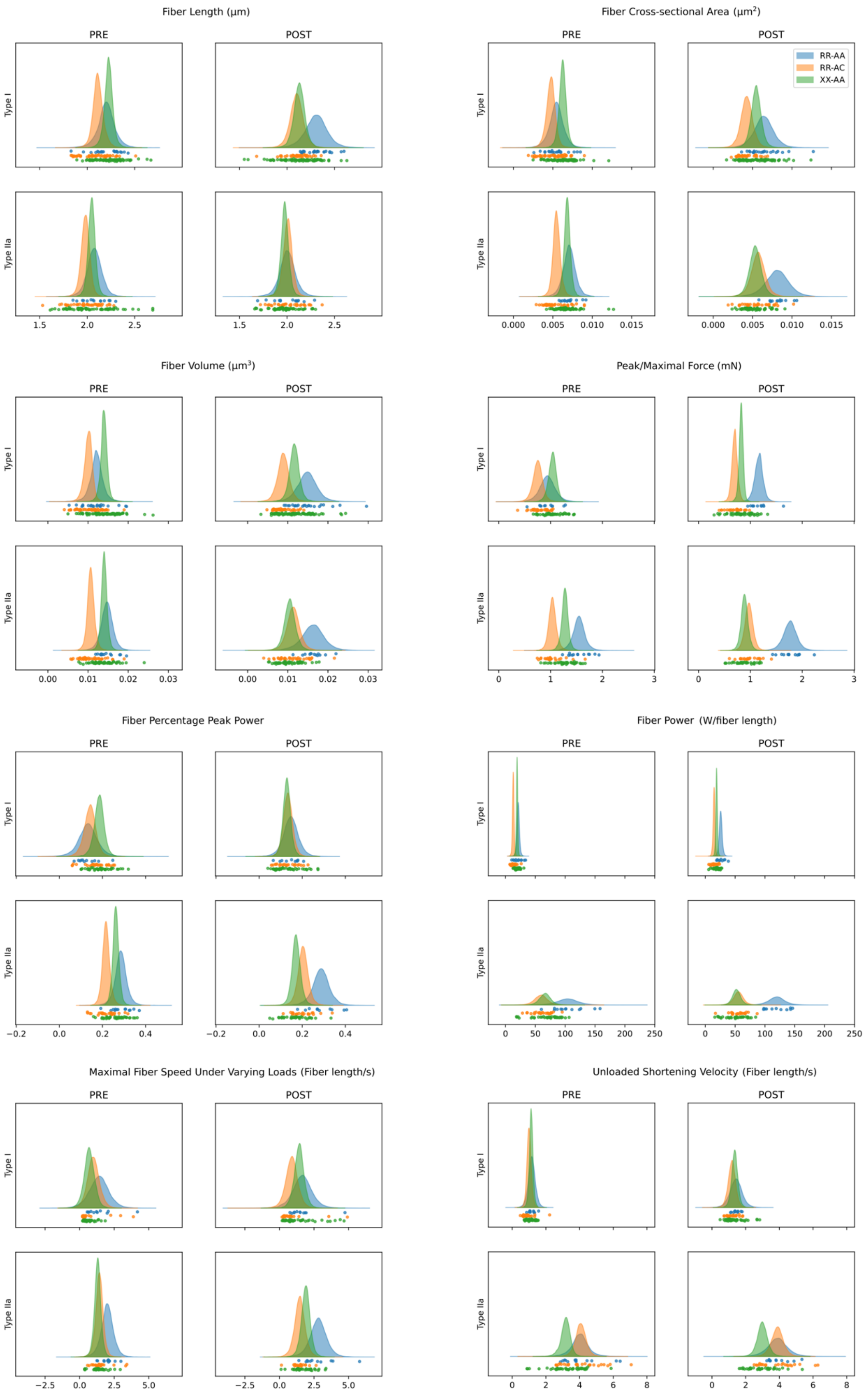
Fiber Length (μm)
Fiber Cross-sectional Area (μm²)
Fiber Volume (μm³)
Peak/Maximal Force (mN)
Fiber Percentage Peak Power
Fiber Power (W/fiber length)
Maximal Fiber Speed Under Varying Loads (Fiber length/s)
Unloaded Shortening Velocity (Fiber length/s)
PRE
POST
Type I
Type IIa
RR-AA
RR-AC
XX-AA

Supplementary Figure 7. Single muscle fiber experiments show a higher maximal stable force and fiber power in p.Glu635 compared with p.635Ala. Muscle biopsies from eight healthy young men (four Arg/Arg and four Ter/Ter at p.Arg577Ter) obtained before (pre) and after (post) an eccentric exercise bout were used to isolate single fibers, which were then functionally characterized. Of the four Arg/Arg carriers, one was heterozygous at p.Glu635Ala (46 fibers) and three were homozygous for the p.Glu635 variant (32±5 fibers). All four Ter/Ter carriers were homozygous for the p.Glu635 variant (39±6 fibers). Posterior distributions from 15,000 iteration Markov chain Monte Carlo models are shown separately for type I and type II_A fibers pre and post eccentric intervention.

SUPPLEMENTARY BOX 1

A Brief description of candidate genes prioritized by at least two approaches using information from Genecards, NCBI and Uniprot.

For MVPA and LST

CHST10 (chr 2, flagged by the intronic *LINC01104* variants rs4303732 (LST) and rs1160545 (MVPA)
Prioritized by: Activity by contact in adipose tissue, coronary artery, ovary; SMR brain (lenient)
Carbohydrate sulfotransferase 10 participates in the biosynthesis of human natural killer-1 (HNK-1) carbohydrate structure, which is involved in neurodevelopment and synaptic plasticity of the hippocampus.

MST1R (chr 3, flagged by the intronic *MST1R* variant rs7615206 for MVPA and LST)
Prioritized by: SMR skeletal muscle; activity by contact in skeletal muscle and fetal thymus; altered expression in skeletal muscle following resistance training (lenient)
Also: in a locus under selection in the past 50,000 years[2]
Macrophage Stimulating 1 Receptor is a cell surface receptor for macrophage-stimulating protein (MSP) that has tyrosine kinase activity. It is expressed at the protein level on the ciliated epithelia of the mucociliary transport apparatus of the lung[27], and together with MSP plays a role in host defense. MST1R regulates physiological processes that include cell survival, migration and differentiation. Ligand binding at the cell surface induces autophosphorylation of RON[28], which activates the wound healing response by promoting epithelial cell migration, proliferation and survival at the wound site[29,30]. Following activation, MST1R interacts with PIK3R1, PLCG1 or the adapter GAB1. Recruitment of these downstream effectors by RON leads to the activation of several signaling cascades including the RAS-ERK, PI3 kinase-AKT, or PLCgamma-PKC. MST1R also plays a role in the innate immune response by regulating the migration and phagocytic activity of macrophages.

For MVPA

LONRF2 (chr 2, flagged by the intronic *LINC01104* variants rs4303732 (LST) and rs1160545 (MVPA)
Prioritized by: Activity by contact in coronary artery and ovary; SMR blood (lenient) for MVPA. Only prioritized by Activity by contact in coronary artery and ovary for LST
LON peptidase N-terminal domain and ring finger 2 is predicted to enable metal ion binding activity.

AKAP10 (chr 17, flagged by rs385301 downstream of *AKAP10*
Prioritized by: Activity by contact in pancreas, adipose tissue, coronary artery, cardiac ventricle, skeletal muscle, ovary, derived neuronal progenitor cultured cells; SMR skeletal muscle (lenient); DEPICT gene prioritization (lenient).

Encoded by a gene in a locus previously identified for physical activity[31], alpha-kinase anchoring protein 10 is known to confine regulatory subunits of protein kinase A to discrete regions of mitochondria[32]. Animal studies have shown evidence for a role of the protein encoded by *AKAP10* in heart rhythm regulation[33], but skeletal muscle phenotypes were not reported previously in knockout models. However, A-kinase anchoring proteins (AKAPs) partially restrict cAMP-PKA signaling, especially in highly structured cell types like skeletal myofibers[34]. cAMP signaling participates in muscle development and regeneration mediated by muscle precursor cells[35].

SPECC1 (chr 17, flagged by rs385301 downstream of *AKAP10*
Prioritized by: Activity by contact in coronary artery and cardiac ventricle; DEPICT gene prioritization (lenient).
Sperm antigen with calponin homology and coiled-coil domains 1 belongs to the cytospin-A family and is highly expressed in testis.

For LST

KDM4A (chr 1, flagged by the intronic *KDM4A* variant rs71658797)
Prioritized by: Activity by contact in pancreas, skeletal muscle, adipose tissue, thymus, ovary, derived neuronal progenitor cultured cells, bipolar neuron from iPSC; and DEPICT gene prioritization (lenient)
Lysine Demethylase 4A is a nuclear protein that functions as a trimethylation-specific histone demethylase and as a transcriptional repressor. It is crucial for muscle differentiation and promotes transcriptional activation of MYOG[36], which in turn is essential for the development of functional embryonic skeletal muscle.

AK5 (chr 1, flagged by the intronic *AK5* variant rs3791033)
Prioritized by: DEPICT tissue and gene prioritization; SMR brain (lenient)
Adenylate Kinase 5 is a cytosolic protein that is exclusively expressed in the brain. It plays a role in regulating the adenine nucleotide composition in a cell by catalyzing the reversible transfer of the terminal phosphate group between nucleoside triphosphates and monophosphates[37].

DNM3 (chr 1, flagged by the intronic DNM3 variant rs6685030)
Prioritized by: Activity by contact in neuronal progenitors, adrenal gland and skeletal muscle; SMR blood (lenient)
Dynamins represent a subfamily of GTP-binding proteins, which are associated with microtubules and bind actin and other cytoskeletal proteins. DNM3 plays a role in the development of megakaryocytes and vesicle-mediated transport and endocytosis.

LRPPRC (chr 2, flagged by the indel rs145255225 (a.k.a. rs34908368))
Prioritized by: SMR brain (lenient); altered expression in skeletal muscle following resistance training (lenient).

This gene encodes a Leucine-rich pentatricopeptide repeat containing protein. Mutations in *LRPPRC* cause a monogenic mitochondrial disease (Leigh syndrome French Canadian Type) that involves severe muscle and movement problems[38]. In addition to the altered expression in skeletal muscle following resistance training we observed, LRPPRC is also up-regulated by exposure to environmental enrichment that is a complex combination of physical, cognitive, and social stimuli in striatum, which may improve locomotor performance[39].

SCN2A (chr 2, flagged by the *SCN2A* missense variant rs114590429)
Prioritized by: DEPICT gene prioritization; running speed, distance and time run in mice (lenient)
SCN2A encodes Sodium Voltage-Gated Channel Alpha Subunit 2, which mediates the voltage-dependent sodium ion permeability of excitable membranes. Mutations in this gene have been associated with seizure disorders, autism spectrum disorder and general cognitive ability[40]. *Scn2a* haploinsufficient mice display a spectrum of phenotypes affecting anxiety, sociability, memory flexibility that commonly observed in models of schizophrenia and autism spectrum disorder[41].

RNF123 (chr 3, flagged by the intronic *MST1R* variant rs7615206)
Prioritized by: SMR brain; DEPICT gene prioritization (lenient) for LST. Only prioritized by SMR brain (lenient) for MVPA
The *RNF123* gene encodes E3 ubiquitin-protein ligase, a motif present in a variety of functionally distinct proteins and known to be involved in protein-protein and protein-DNA interactions. It promotes the ubiquitination and proteasome-mediated degradation of CDKN1B, which is the cyclin-dependent kinase inhibitor at the G0-G1 transition of the cell cycle by the ubiquitin-proteasome pathway [42,43]. It also functions as a novel inhibitor of innate antiviral signaling, independently of its E3 ligase activity[44]. This gene is more highly expressed in skeletal muscle than in other tissues. Recent studies involving UK Biobank samples also associated the locus with musculoskeletal pain[45,46].

SEMA3F *(chr 3, flagged by* the intronic *MST1R* variant rs7615206; group 2 LST locus)
Prioritized by: SMR brain; Activity by contact in pancreas, adipose tissue, hepatocytes, bipolar neuron from iPSC. Not prioritized for MVPA
Semaforin 3F – encoded by *SEMA3F* - is involved in axon guidance during neuronal development. This gene is expressed in endothelial cells where it induces apoptosis, inhibits cell proliferation and survival, and acts as an anti-tumorigenic agent.

FOXP1 *(chr 3, flagged by the intronic SAMMSON variant rs76267866)*
Prioritized by: DEPICT gene prioritization; Activity by contact in adipose tissue, adrenal gland, skeletal muscle, myotube, astrocyte
Forkhead Box P1 acts as a tumor suppressor and is involved in regulation of cardiac muscle cell proliferation and columnar organization of spinal motor neurons. It also plays a role in B-cell development and promotes the formation of the lateral motor neuron column (LMC) and the preganglionic motor column (PGC) and is required for appropriate motor axon projections.

CADM2 (chr 3; flagged by the intronic CADM2 variants rs1691471 (MVPA) and rs1375561 (LST)
Prioritized by: DEPICT tissue enrichment; DEPICT gene prioritization (nominal); SMR skeletal muscle. Only prioritized by altered expression in skeletal muscle following resistance training (lenient) for LST.
CADM2 (also known as SynCAM2, Igsf4d, and Nectin-like molecule 3) encodes the synaptic cell adhesion molecule 2. SNPs in the locus have been associated with a series of psychological traits, such as educational attainment[47]; self-reported physical activity[31]; risk-taking behaviour[48]; alcohol consumption[49]; substance use and risk-taking[50]; and obesity[51]. In addition to lower adiposity, lower systemic glucose levels, and better insulin sensitivity, *Cadm2*-knockout mice exhibited more locomotor activity, higher energy expenditure, and higher core body temperature, suggesting *cadm2* is a potent regulator of systemic energy homeostasis[52]. While C*ADM2* is predominantly expressed in the brain, the top SMR SNP for MVPA in skeletal muscle (rs382210) is in LD with the lead MVPA GWAS SNP rs1691471 (r2=0.29, D'=0.95), it is independent of the previously identified BMI-associated SNP rs13078960 (r2=0.03, D'=0.48)[51]). This suggests that while *CADM2* likely influences other complex traits through the brain, it possibly influences PA locally through skeletal muscle.

HTR1F *(chr 3, flagged by the intronic HTR1F variant rs17025214)*
Prioritized by: DEPICT gene prioritization; Activity by contact in thyroid
5-Hydroxytryptamine receptor 1F is primarily located in the hippocampus, cortex and dorsal raphe nucleus and enables G protein-coupled serotonin receptor activity and serotonin binding activity. It also functions as a receptor for various alkaloids and psychoactive substances.

APC *(chr 5, flagged by the intronic APC variant rs396321)*
Prioritized by: DEPICT gene prioritization and tissue enrichment; Activity by contact in skeletal muscle
APC regulator of Wnt signaling pathway acts as an antagonist of the Wnt signaling pathway. It is also involved in cell migration and adhesion, transcriptional activation and apoptosis. It is a tumor suppressor.

REEP5 (chr 5, flagged by the intronic *APC* variant rs396321)
Prioritized by: DEPICT gene prioritization; SMR skeletal muscle (lenient)
Receptor Accessory Protein 5 (*REEP5*) expression is muscle-specific, with the highest protein expression in the mouse ventricles and skeletal muscle. *In vitro* and *in vivo* experiments have demonstrated that the protein encoded by *REEP5* plays a critical role in sarco/endoplasmic reticulum organization and function, as well as in normal heart function and development[53].

SIL1 (chr 5, flagged by the intronic *SIL1* indel rs752485316)
Prioritized by: SMR brain; Activity by contact in adrenal gland, cardiac ventricle and ovary; altered expression in skeletal muscle following resistance training (lenient)
SIL1 (SIL1 Nucleotide Exchange Factor) encodes a resident endoplasmic reticulum (ER), N-linked glycoprotein with an N-terminal ER targeting sequence, 2 putative N-glycosylation sites, and a C-terminal ER retention signal. Mutations in this gene have been associated with

Marinesco-Sjogren syndrome, which is clinically characterized by progressive myopathy and other tissue pathologies. Experimental characterization in mice reveals a disruption in ER homeostasis upon SIL1 loss, leading to loss of skeletal muscle mass, strength and function[54].

SOBP (chr 6, flagged by the intronic *PDSS2* variant rs78394231)
Prioritized by: DEPICT gene prioritization (lenient); Activity by contact in adrenal gland, bipolar neuron from iPSC, skeletal muscle, cardiac ventricle, ovary, thryoid
Sine oculis binding protein homolog is involved in development of the cochlea and has been linked to intellectual disability.

REPS1 (chr 6, flagged by the intronic *REPS1* deletion rs200307517)
Prioritized by: SMR brain (lenient); altered expression in skeletal muscle following resistance training (lenient)
REPS1 (RALBP1 Associated Eps Domain Containing 1) encodes a signaling adaptor protein with two EH domains that interacts with proteins that participate in signaling, endocytosis and cytoskeletal changes.

PDE10A (chr 6; flagged by the intronic *PDE10A* SNP rs58541850)
Prioritized by: altered expression in skeletal muscle following resistance training; DEPICT gene prioritization
Phosphodiesterase 10A plays a role in signal transduction by regulating the intracellular concentration of cyclic nucleotides. The protein can hydrolyze cAMP and cGMP, and may play a critical role in regulating cAMP and cGMP levels in the striatum[55], a region of the brain contributing to the control of movement and cognition. cAMP and cGMP both mediate the effects of dopamine D1 and D2 receptors on the activity of medium-sized spiny neurons[56]. Pharmacological inhibition of PDE10A increases cAMP and cGMP levels; and increases striatal output[57].

IMMP2L (chr 7; flagged by the intronic *IMMPL2* SNP rs2529484)
Prioritized by: altered expression in skeletal muscle following resistance training; SMR skeletal muscle (lenient); Activity by contact in coronary artery and liver;
Inner Mitochondrial Membrane Peptidase Subunit 2 resides in the mitochondria and is required for the catalytic activity of the mitochondrial inner membrane peptidase (IMP) complex. It catalyzes the removal of transit peptides required to transport proteins from the mitochondrial matrix, across the inner membrane, to the intermembrane space[58].

EXOC4 (chr 7; flagged by the intronic lead SNP rs13235840 in *EXOC4* and *LOC101928861*)
Prioritized by: altered expression in skeletal muscle following resistance training; DEPICT gene prioritization;
Exocyst Complex Component 4 is part of the highly conserved exocyst complex that is essential for targeting exocytic vesicles to specific docking sites on the plasma membrane. Exocyst Complex Component 4 participates in GLUT4 translocation and docking to the plasma membrane[59], and is essential for insulin-stimulated glucose uptake in skeletal muscle[59].

MKRN1 (chr 7; flagged by the intronic *MKRN1* lead SNP rs17621391)
Prioritized by: DEPICT gene prioritization (lenient); Activity by contact in pancreas, adrenal gland, bipolar neuron from iPSC, skeletal muscle, hepatocyte, ovary, thymus
Makorin ring finger protein 1 is thought to regulate RNA polymerase II-catalyzed transcription. It keeps cells alive by suppressing p53/TP53 under normal conditions, but stimulates apoptosis by repressing CDKN1A under stress.

BLK (chr 8, flagged by the intronic *XKR6* variant rs7821826)
Prioritized by: DEPICT tissue enrichment; SMR blood (lenient)
BLK Proto-Oncogene, Src Family Tyrosine Kinase is a nonreceptor tyrosine-kinase of the Src family of proto-oncogenes that are typically involved in cell proliferation and differentiation. Mutations at the *BLK* locus have been linked to Maturity-onset diabetes of the young (MODY) and β-cell dysfunction[60]. In pancreatic islets, it acts as a modulator of beta-cells function through the up-regulation of PDX1 and NKX6-1 and consequent stimulation of insulin secretion in response to glucose [60].

PACS1 (chr 11, flagged by the intronic *PACS1* variant rs4483592)
Prioritized by: SMR brain; DEPICT gene prioritization
Phosphofurin Acidic Cluster Sorting Protein 1 is involved in the trans-Golgi-membrane traffic[61]. A *de novo* mutation in *PACS1* was recently shown to cause defective migration of cranial-neural-crest cells and resulted in an intellectual disability syndrome and global developmental delay[62].

KLC2 (chr 11, flagged by the intronic *PACS1* variant rs4483592)
Prioritized by: SMR blood and skeletal muscle; DEPICT gene prioritization and tissue enrichment; Activity by contact in derived neural progenitors, adipose tissue, cardiac ventricle, hepatocytes and fetal thymus
KCL2 encodes Kinesin Light Chain 2, a light chain of kinesin and molecular motor responsible for moving vesicles and organelles along microtubules. Defects in this gene cause the rare, autosomal recessive mendelian disorder Spastic Paraplegia, Optic Atropy, and Neuropathy (SPOAN) Syndrome[63]. This syndrome is characterized by an early-onset, progressive weakness and spasticity of the legs. Zebrafish embryos with morpholino-mediated downregulation of *klc2* had a dose-dependent, shortened, twisted tail and were unable to swim. A similar motor phenotype was observed in zebrafish embryos upon upregulation of *klc2*[64].

RAB1B (chr11, flagged by the intronic *PACS1* variant rs4483592)
Prioritized by: DEPICT gene prioritization (lenient); Activity by contact in skeletal muscle, mytube, thymus
RAB1B member RAS oncogene Family functions in the early secretory pathway and is essential for vesicle transport between the endoplasmic reticulum and Golgi[65].

CNIH2 (chr11, flagged by the intronic *PACS1* variant rs4483592)
Prioritized by: DEPICT gene prioritization; Activity by contact in bipolar neuron from iPSC, thymus, hepatocyte

Cornichon family AMPA receptor auxiliary protein 2 mediates fast synaptic neurotransmission in the CNS and plays a role in assembly of hippocampal AMPA receptor complexes, thus modulating receptor gating and pharmacology.

TMEM151A (chr11, flagged by the intronic *PACS1* variant rs4483592)
Prioritized by: DEPICT gene prioritization; Activity by contact in bipolar neuron from iPSC, pancreas, hepatocyte
Transmembrane protein 151A is predicted to be an integral component of membranes.

MLF2 (chr12, flagged by rs3759344 upstream of *MLF2*)
Prioritized by: DEPICT gene prioritization (lenient); Activity by contact in derived neuronal progenitor cultured cells, pancreas, adipose tissue, adrenal gland, astrocyte, bipolar neuron from iPSC, cardiac muscle, coronary artery, skeletal muscle, cardiac ventricle, hepatocyte, myotube, ovary, thymus
Myeloid leukemia factor 2 is a membrane protein that is predicted to be involved in the regulation of transcription. Diseases associated with this gene include fatal infantile hypertonic myofibrillar myopathy

PTMS (chr12, flagged by rs3759344 upstream of *MLF2*)
Prioritized by: DEPICT tissue enrichment; Activity by contact in adrenal gland, astrocyte, pancreas, adipose tissue, cardiac muscle, skeletal muscle, hepatocyte, liver, myotube, thyroid
Parathymosin is predicted to be a nuclear protein that is involved in DNA replication and may mediate immune function.

COPS7A (chr12, flagged by rs3759344 upstream of *MLF2*)
Prioritized by: DEPICT gene prioritization (lenient); Activity by contact in cardiac muscle, liver
COP9 signalosome subunit 7A is a component of the COP9 signalosome complex that plays a role in various cellular and developmental processes.

MMAB (chr 12, flagged by the intronic *MYO1H* variant rs7969719)
Prioritized by: DEPICT tissue prioritization; SMR skeletal muscle (lenient)
MMAB (Metabolism Of Cobalamin Associated B) encodes a protein that catalyzes the final step in the conversion of vitamin B(12) into adenosylcobalamin (AdoCbl), a vitamin B12-containing coenzyme for methylmalonyl-CoA mutase. GWAS has reported variants in this gene to be associated with Apolipoprotein A1, HDL, and BMI, amongst others[66-68].

TESC (chr 12; flagged by the intronic *TESC* lead SNP rs2173650)
Prioritized by: SMR brain; association with daily running distance and average voluntary running speed in mice
Tescalcin, also known as Calcineurin B Homologous Protein 3 is highly expressed in the striatum[69], which harbours the central reward system and which represents a major site of physical activity regulation[70-72]. *TESC* encodes a Ca^{2+}- and Mg^{2+} -binding protein that is essential for extracellular signal-regulated kinase (ERK) cascade activation, which in turn is critical for normal cell differentiation[73], as well as for the motivating effects of reward-associated

stimuli along with other important roles related to learning, reinforcing and addiction in the striatum[74].

FBXO21 (chr 12, flagged by the intronic *TESC* lead SNP rs2173650)
Prioritized by: SMR brain (lenient); spontaneous running speed and distance in mice
FBXO21 encodes F-Box Protein 21, a member of the F-box protein family, fbxs, containing either different protein-protein interaction modules or no recognizable motifs. It is anticipated to play a role in the innate immune function.

ARL6IP4 (chr 12, flagged by the intronic *PITPNM2* indel rs541140319 (aka rs59131741))
Prioritized by: SMR blood; spontaneous running speed in mice (lenient)
ADP Ribosylation Factor Like GTPase 6 Interacting Protein 4 is involved in modulating alternative pre-mRNA splicing with either 5' distal site activation or preferential use of 3' proximal site.

OGFOD2 (chr 12, flagged by the intronic *PITPNM2* indel rs541140319 (aka rs59131741))
Prioritized by: SMR brain and blood; spontaneous running speed in mice (lenient)
Gene Ontology annotations related to *OGFOD2* (2-Oxoglutarate And Iron Dependent Oxygenase Domain Containing 2) include iron ion binding and oxidoreductase activity, acting on paired donors, with incorporation or reduction of molecular oxygen, using 2-oxoglutarate as a donor, and incorporation of one atom each of oxygen into both donors.

CCDC92 (chr12, flagged by the intronic *PITPNM2* indel rs541140319 (aka rs59131741))
Prioritized by: SMR brain (lenient); SMR skeletal muscle (lenient); spontaneous running speed in mice (lenient)
CCDC92 is a coiled coil domain protein which interacts with proteins in the centriole/ciliary interface[75]. The *CCDC92* locus has been associated with higher insulin, higher triglyceride, and lower HDL-cholesterol levels. Further experimental studies showed that knockout of *CCDC92* resulted in less lipid accumulation in a mouse model. These results suggested a role for *CCDC92* in adipocyte differentiation[76].

FARP1 (chr 13; flagged by the intronic *FARP1* lead SNP rs9513416)
Prioritized by: altered expression in skeletal muscle following resistance training; DEPICT gene prioritization
FARP1 encodes FERM, ARH/RhoGEF And Pleckstrin Domain Protein 1, which promotes dendritic growth in neurons.

HERC1 (chr 15, flagged by the intronic *HERC1* variant rs12324720)
Prioritized by: DEPICT gene prioritization; altered expression in skeletal muscle following resistance training (lenient)
HECT And RLD Domain Containing E3 Ubiquitin Protein Ligase Family Member 1 encodes a protein that stimulates guanine nucleotide exchange on ARF1 and Rab proteins and may be involved in membrane transport processes via some guanine nucleotide exchange factor (GEF) activity and its ability to bind clathrin. HERC1 is involved in the ubiquitin-proteasome system, for

which the role in cachexia and sarcopenia is well-described[77]. An Intronic *HERC1* variant was associated with heel bone mineral density in UK BioBank data[78].

CBX4 (chr 17, flagged by the *CBX8* 3' UTR variant rs73420302)
Prioritized by: DEPICT gene prioritization; Activity by contact in pancreas
Chromobox 4 is involved in the negative regulation of transcription by RNA polymerase II.

CELF4 (chr 18, flagged by the intronic SNP rs12962050)
Prioritized by: DEPICT tissue enrichment and gene prioritization; Activity by contact in skeletal muscle, pancreas, adrenal gland, bipolar neuron from iPSC, coronary artery, hepatocyte
CUGBP Elav-like family member 4 belongs to a protein family that regulates pre-mRNA alternative splicing. It specifically activates exon 5 inclusion of cardiac isoforms of troponin 2 (TNNT2) during heart remodeling and the juvenile to adult transition[79].

YWHAB (chr 20, flagged by the intronic *YWHAB* indel rs139900206 (a.k.a. rs3838037))
Prioritized by: Finemapp & CADD score; Activity by contact in white adipose tissue; SMR brain (lenient); altered expression in skeletal muscle following resistance training (lenient)
YWHAB (Tyrosine 3-Monooxygenase/Tryptophan 5-Monooxygenase Activation Protein Beta, also known as 14-3-3 protein) encodes an adapter protein that is implicated in the regulation of a large spectrum of both general and specialized signaling pathways and plays a role in the cell cycle[80]. Previous proteomic analyses showed expression of YWHAB is upregulated in rat dorsal hippocampus following consumption of a diet high in fat and refined sugar[81], as well as in plasma after exercise[82].

STK4 (chr 20, flagged by the intronic *YWHAB* indel rs139900206 (a.k.a. rs3838037))
Prioritized by: SMR brain (lenient); Activity by contact in white adipose tissue
Serine/Threonine kinase 4 is a cytoplasmic, stress-activated kinase that can phosphorylate myelin basic protein and undergoes autophosphorylation. Following caspase-cleavage it enters the nucleus and induces chromatin condensation, followed by internucleosomal DNA fragmentation. The phosphorylation that is catalyzed by this protein has been associated with apoptosis.

ZBTB46 (chr 20, flagged by the intronic *ZBTB46* variant rs6010651)
Prioritized by: altered expression in skeletal muscle following resistance training; Activity by contact in bipolar neuron from iPSC; SMR blood (lenient)
Gene Ontology (GO) annotations for *ZBTB46* (Zinc Finger And BTB Domain Containing 46) include nucleic acid binding. *ZBTB46* functions as a transcriptional repressor for PRDM1 that mediates a transcriptional program in various immune tissue-resident lymphocyte T cell types.

STUDY SPECIFIC ACKNOWLEDGEMENTS AND FUNDING

AGES

Age, Gene/Environment Susceptibility-Reykjavik Study (AGES) was funded by NIH contract N01-AG-1-2100 and HHSN271201200022C, the NIA Intramural Research Program, Hjartavernd (the Icelandic Heart Association), and the Althingi (the Icelandic Parliament)

ALSPAC

The UK Medical Research Council and Wellcome (Grant ref: 217065/Z/19/Z) and the University of Bristol provide core support for ALSPAC. This publication is the work of the authors and Marcel den Hoed and Zhe Wang will serve as guarantors for the contents of this paper.
We are extremely grateful to all the families who took part in this study, the midwives for their help in recruiting them, and the whole ALSPAC team, which includes interviewers, computer and laboratory technicians, clerical workers, research scientists, volunteers, managers, receptionists and nurses.

ARIC

The Atherosclerosis Risk in Communities study has been funded in whole or in part with Federal funds from the National Heart, Lung, and Blood Institute, National Institutes of Health, Department of Health and Human Services (contract numbers HHSN268201700001I, HHSN268201700002I, HHSN268201700003I, HHSN268201700004I and HHSN268201700005I), R01HL087641, R01HL059367 and R01HL086694; National Human Genome Research Institute contract U01HG004402; and National Institutes of Health contract HHSN268200625226C. The authors thank the staff and participants of the ARIC study for their important contributions. Infrastructure was partly supported by Grant Number UL1RR025005, a component of the National Institutes of Health and NIH Roadmap for Medical Research.

B58C

The management of the 1958 Birth Cohort is funded by the Economic and Social Research Council (grant number ES/M001660/1). Access to these resources was enabled via the 58READIE Project funded by Wellcome Trust and Medical Research Council (grant numbers WT095219MA and G1001799). DNA collection was funded by MRC grant G0000934 and cell-line creation by Wellcome Trust grant 068545/Z/02. This study makes use of data generated by the Wellcome Trust Case-Control Consortium. A full list of investigators who contributed to generation of the data is available from the Wellcome Trust Case-Control Consortium website. Funding for the project was provided by the Wellcome Trust under the award 076113. This research used resources provided by the Type 1 Diabetes Genetics Consortium, a collaborative clinical study sponsored by the National Institute of Diabetes and Digestive and Kidney Diseases (NIDDK), National Institute of Allergy and Infectious Diseases, National Human Genome Research Institute, National Institute of Child Health and Human Development, and Juvenile Diabetes Research Foundation International (JDRF) and supported by U01 DK062418.

BioMe
The Mount Sinai BioMe Biobank is supported by The Andrea and Charles Bronfman Philanthropies. We thank all participants and all our recruiters who have assisted and continue to assist in data collection and management. We are grateful for the computational resources and staff expertise provided by Scientific Computing at the Icahn School of Medicine at Mount Sinai.

BPROOF
We thank the participants of the B-PROOF study for their enthusiasm and cooperation. Furthermore, we thank the dedicated research team that conducted the study. This study is supported and funded by The Netherlands Organization for Health Research and Development (ZonMw, Grant 6130.0031), the Hague; unrestricted grant from NZO (Dutch Dairy Association), Zoetermeer; Orthica, Almere; NCHA (Netherlands Consortium Healthy Ageing) Leiden/Rotterdam; Ministry of Economic Affairs, Agriculture and Innovation (project KB-15-004-003), the Hague; Wageningen University, Wageningen; VUmc, Amsterdam; Erasmus Medical Center, Rotterdam; Unilever, Colworth, UK. All organisations, except Unilever, are based in the Netherlands. The sponsors have no role in the design or implementation of the study, data collection, data management, data analysis, data interpretation, or in the preparation, review, or approval of the manuscript.

Busselton
The Busselton Health Study acknowledges the generous support for the 1994/5 follow-up study from Healthway, Western Australia and the numerous Busselton community volunteers who assisted with data collection and the study participants from the Shire of Busselton. The Busselton Health Study is supported by The Great Wine Estates of the Margaret River region of Western Australia.

CARDIA
The Coronary Artery Risk Development in Young Adults Study (CARDIA) is conducted and supported by the National Heart, Lung, and Blood Institute (NHLBI) in collaboration with the University of Alabama at Birmingham (HHSN268201800005I & HHSN268201800007I), Northwestern University (HHSN268201800003I), University of Minnesota (HHSN268201800006I), and Kaiser Foundation Research Institute (HHSN268201800004I). CARDIA was also partially supported by the Intramural Research Program of the National Institute on Aging (NIA) and an intra-agency agreement between NIA and NHLBI (AG0005). Genotyping was funded as part of the NHLBI Candidate-gene Association Resource (N01-HC-65226) and the NHGRI Gene Environment Association Studies (GENEVA) (U01-HG004729, U01-HG04424, and U01-HG004446). This manuscript has been reviewed and approved by CARDIA for scientific content.

CHS
Cardiovascular Health Study: This CHS research was supported by NHLBI contracts HHSN268201200036C, HHSN268200800007C, HHSN268201800001C, N01HC55222, N01HC85079, N01HC85080, N01HC85081, N01HC85082, N01HC85083, N01HC85086,

75N92021D00006; and NHLBI grants U01HL080295, R01HL085251, R01HL087652, R01HL105756, R01HL103612, R01HL120393, and U01HL130114 with additional contribution from the National Institute of Neurological Disorders and Stroke (NINDS). Additional support was provided through R01AG023629 from the National Institute on Aging (NIA). A full list of principal CHS investigators and institutions can be found at CHS-NHLBI.org.
The content is solely the responsibility of the authors and does not necessarily represent the official views of the National Institutes of Health.
The provision of genotyping data was supported in part by the National Center for Advancing Translational Sciences, CTSI grant UL1TR001881, and the National Institute of Diabetes and Digestive and Kidney Disease Diabetes Research Center (DRC) grant DK063491 to the Southern California Diabetes Endocrinology Research Center.

CoLaus

The CoLaus study was and is supported by research grants from GlaxoSmithKline, the Faculty of Biology and Medicine of Lausanne, and the Swiss National Science Foundation (grants 33CSCO-122661, 33CS30-139468, 33CS30-148401 and 33CS30_177535/1).

DNBC

The Danish National Birth Cohort (DNBC) is a result of major grants from the Danish National Research Foundation, the Danish Pharmacists' Fund, the Egmont Foundation, the March of Dimes Birth Defects Foundation, the Augustinus Foundation, and the Health Fund of the Danish Health Insurance Societies. The DNBC biobank is a part of the Danish National Biobank resource, which is supported by the Novo Nordisk Foundation. The generation of GWAS genotype data for the DNBC samples was carried out within the Gene Environment Association Studies (GENEVA) consortium with funding provided through the National Institutes of Health's Genes, Environment, and Health Initiative (U01HG004423; U01HG004446; U01HG004438).
We are very grateful to all DNBC families who took part in the study. We would also like to thank everyone involved in data collection and biological material handling.

EGCUT

This research was supported by the European Union through the European Regional Development Fund (Project No. 2014-2020.4.01.15-0012), by the Estonian Research Council grant PUT (PRG687), by the Estonian Research Council grant PUT (PRG1291). We acknowledge the Estonian Biobank research team. Data analyses were carried out in part in the High-Performance Computing Center of University of Tartu.

EPIC-Norfolk

We are grateful to all the participants who have been part of the EPIC-Norfolk project and to the many members of the study teams at the University of Cambridge who have enabled this research. The study is funded by Medical Research Council (MR/N003284/1, MC-UU_12015/1, MC_PC_13048) Cancer Research UK (C864/A14136).

FamHS

The FamHS is funded by R01HL118305 and R01HL117078 NHLBI grants, and 5R01DK07568102 and 5R01DK089256 NIDDK grant.

FAMILY

We would like to thank all the participants of the FAMILY study. We acknowledge the internal support from the Population Health Research Institute for centralizing the data collect. The FAMILY genetic study has been funded by the Heart and Stroke Foundation of Ontario (grant # NA 7293 “Early genetic origins of cardiovascular risk factors”). The FAMILY study was funded by the Hamilton Health Science Foundation, the Canadian Institutes of Health Research and by Heart & Stroke Foundation of Ontario as well as additional grants from the Population Health Research Institute internal funds.

Fenland

The Fenland Study (10.22025/2017.10.101.00001) is funded by the Medical Research Council (MC_UU_12015/1). We are grateful to all the volunteers and to the General Practitioners and practice staff for assistance with recruitment. We thank the Fenland Study Investigators, Fenland Study Co-ordination team and the Epidemiology Field, Data and Laboratory teams. We further acknowledge support for genomics from the Medical Research Council (MC_PC_13046).

FHS

The Framingham Heart Study is funded by 75N92019D00031

FUSION

Support for FUSION was provided by NIH grants R01-DK062370 (to M.B.) and intramural project number 1Z01-HG000024 (to F.S.C.). Genome-wide genotyping was conducted by the Johns Hopkins University Genetic Resources Core Facility SNP Center at the Center for Inherited Disease Research (CIDR), with support from CIDR NIH contract no. N01-HG-65403.

GENOA

Genetic Epidemiology Network of Arteriosclerosis (GENOA) was supported by the National Institutes of Health grant numbers HL054457, HL054464, HL054481, HL087660, and HL119443 from the National Heart, Lung, and Blood Institute. Genotyping was performed at the Mayo Clinic by Stephan T. Turner, MD, Mariza de Andrade PhD, Julie Cunningham, PhD. We thank Eric Boerwinkle, PhD and Megan L. Grove from the Human Genetics Center and Institute of Molecular Medicine and Division of Epidemiology, University of Texas Health Science Center, Houston, Texas, USA for their help with genotyping. We would also like to thank the families that participated in the GENOA study.

GEOS/BWYSS

GEOS/BWYSS was supported by the Department of Veterans Affairs Biomedical Laboratory Research and Development Service, the Centers for Disease Control and Prevention, and the National Institutes of Health (Grants: R01 NS45012 and R01 NS105150).

GOOD
The GOOD study was funded by the Swedish Research Council, the Swedish state under the agreement between the Swedish government and the county councils, the ALF-agreement, the Lundberg Foundation, the Torsten Söderberg Foundation, the Novo Nordisk Foundation and the Knut and Alice Wallenberg Foundation.

GOYA
The GOYA study was conducted as part of the activities of the Danish Obesity Research Center(DanORC,www.danorc.dk) and the MRC centerfor Causal Analyses in Translational Epidemiology (MRC CAiTE).

GRAPHIC
The GRAPHIC Study was funded by the British Heart Foundation (RG/200004).

HANDLS
The Healthy Aging in Neighborhoods of Diversity across the Life Span study is supported by the National Institute on Aging Intramural Research Program, NIH Project number AG000513. We thank the HANDLS participants for agreeing to donate samples for the study. We also recognize the HANDLS medical staff for their careful evaluation of study participants.

HCHS/SOL
The authors thank the staff and participants of HCHS/SOL for their important contributions. Investigators website - http://www.cscc.unc.edu/hchs/ The Hispanic Community Health Study/Study of Latinos is a collaborative study supported by contracts from the National Heart, Lung, and Blood Institute (NHLBI) to the University of North Carolina (HHSN268201300001I / N01-HC-65233), University of Miami (HHSN268201300004I / N01-HC-65234), Albert Einstein College of Medicine (HHSN268201300002I / N01-HC-65235), University of Illinois at Chicago – HHSN268201300003I / N01-HC-65236 Northwestern Univ), and San Diego State University (HHSN268201300005I / N01-HC-65237).
The following Institutes/Centers/Offices have contributed to the HCHS/SOL through a transfer of funds to the NHLBI: National Institute on Minority Health and Health Disparities, National Institute on Deafness and Other Communication Disorders, National Institute of Dental and Craniofacial Research, National Institute of Diabetes and Digestive and Kidney Diseases, National Institute of Neurological Disorders and Stroke, NIH Institution-Office of Dietary Supplements. The Genetic Analysis Center at the University of Washington was supported by NHLBI and NIDCR contracts (HHSN268201300005C AM03 and MOD03)

Health 06
The Health2006 study was financially supported by grants from the Velux Foundation; the Danish Medical Research Council, Danish Agency for Science, Technology and Innovation; the Aase and Ejner Danielsens Foundation; ALK-Abello´ A/S (Hørsholm, Denmark), Timber Merchant Vilhelm Bangs Foundation, MEKOS Laboratories (Denmark) and Research Centre for Prevention and Health, the Capital Region of Denmark. This project was also supported by the Lundbeck Foundation and produced by The Lundbeck Foundation Centre for Applied Medical

Genomics in Personalized Disease Prediction, Prevention and Care (LuCamp, www.lucamp.org). The Novo Nordisk Foundation Center for Basic Metabolic Research is an independent Research Center at the University of Copenhagen partially funded by an unrestricted donation from the Novo Nordisk Foundation (NNF18CC0034900). Further funding came from the Danish Council for Independent Research (Medical Sciences).

HPFS (Health Professional Follow-up Study)

The HPFS and NHS are funded by the National Institute of Deafness and Other Communication Disorders (R03 DC013373 to M.C.C). The NHS (UM1 CA186107) and HPFS (U01 CA167552) are additionally supported by the National Cancer Institute. We thank all participants of the NHS and HPFS for their continued contributions to research.

HRS (Health and retirement study)

HRS is supported by the National Institute on Aging (NIA U01AG009740). The genotyping was funded separately by the National Institute on Aging (RC2 AG036495, RC4 AG039029). Our genotyping was conducted by the NIH Center for Inherited Disease Research (CIDR) at Johns Hopkins University. Genotyping quality control and final preparation of the data was performed by the Genetics Coordinating Center at the University of Washington.

HUFS

The views expressed in this manuscript are those of the authors and do not necessarily represent the views of the National Human Genome Research Institute; the National Institutes of Health; or the U.S. Department of Health and Human Services. This project was supported in part by the Intramural Research Program of the National Human Genome Research Institute of the National Institutes of Health through the Center for Research on Genomics and Global Health (CRGGH). The CRGGH is supported by the National Human Genome Research Institute, the National Institute of Diabetes and Digestive and Kidney Diseases, the Center for Information Technology, and the Office of the Director at the National Institutes of Health (Z01HG200362).

Inchianti / BLSA

The InCHIANTI study baseline (1998-2000) was supported as a "targeted project" (ICS110.1/RF97.71) by the Italian Ministry of Health and in part by the U.S. National Institute on Aging (Contracts: 263 MD 9164 and 263 MD 821336).
The study protocol for both studies were reviewed and approved by the Internal Review Board of the National Institute for Environmental Health Sciences (NIEHS) and all participants provided written informed consent.

Inter99

This project was funded by the Lundbeck Foundation and produced by The Lundbeck Foundation Centre for Applied Medical Genomics in Personalised Disease Prediction, Prevention and Care (LuCamp, www.lucamp.org). The Novo Nordisk Foundation Center for Basic Metabolic Research is an independent Research Center at the University of Copenhagen

partially funded by an unrestricted donation from the Novo Nordisk Foundation (NNF18CC0034900). Further funding came from the Danish Council for Independent Research (Medical Sciences). The Inter99 study was initiated by Torben Jørgensen (PI), Knut Borch-Johnsen (co-PI), Hans Ibsen, and Troels F. Thomsen. The steering committee comprises the former two and Charlotta Pisinger. The study was financially supported by research grants from the Danish Research Council, the Danish Centre for Health Technology Assessment, Novo Nordisk Inc., Research Foundation of Copenhagen County, Ministry of Internal Affairs and Health, the Danish Heart Foundation, the Danish Pharmaceutical Association, the Augustinus Foundation, the Ib Henriksen Foundation, the Becket Foundation, and the Danish Diabetes Association. We are indebted to the staff and participants of the Inter99, cohort for their important contributions. The authors wish to thank the staff at Novo Nordisk Foundation Center for Basic Metabolic Research, University of Copenhagen, Denmark: A. Forman, T. Lorentzen, B. Andreasen and G. J. Klavsen for technical assistance and A. L. Nielsen, P. Sandbeck and G. Lademann for management assistance.

INTERSTROKE

This study was supported by Canadian Stroke Network, Canadian Institutes of Health Research, and Heart & Stroke Foundation

KARE

This work was supported by the Bio-Synergy Research Project (2013M3A9C4078158) of the Ministry of Science, ICT and Future Planning through the National Research Foundation

KORA 3/4

The KORA study was initiated and financed by the Helmholtz Zentrum München – German Research Center for Environmental Health, which is funded by the German Federal Ministry of Education and Research (BMBF) and by the State of Bavaria.

Lifelines Cohort Study

The LifeLines Cohort Study, and generation and management of GWAS genotype data for the LifeLines Cohort Study is supported by the Netherlands Organization of Scientific Research NWO (grant 175.010.2007.006), the Economic Structure Enhancing Fund (FES) of the Dutch government, the Ministry of Economic Affairs, the Ministry of Education, Culture and Science, the Ministry for Health, Welfare and Sports, the Northern Netherlands Collaboration of Provinces (SNN), the Province of Groningen, University Medical Center Groningen, the University of Groningen, Dutch Kidney Foundation and Dutch Diabetes Research Foundation. The authors wish to acknowledge the services of the Lifelines Cohort Study, the contributing research centers delivering data to Lifelines, and all the study participants.

LifeLines Cohort Study authors at the *University of Groningen, University Medical Center Groningen, The Netherlands* are:
Behrooz Z Alizadeh, H Marike Boezen and Harold Snieder (*Department of Epidemiology*); Lude Franke, Morris Swertz and Cisca Wijmenga (*Department of Genetics*); Pim van der Harst (*Department of Cardiology*); Gerjan Navis (*Department of Internal Medicine, Division of*

Nephrology); Marianne Rots (*Department of Medical Biology*); and Bruce HR Wolffenbuttel (*Department of Endocrinology*)

Malmö DC (MDC)

This study was supported by the European Research Council (Consolidator grant nr 649021, Orho-Melander), the Swedish Research Council, the Swedish Heart and Lung Foundation, the Novo Nordic Foundation, the Swedish Diabetes Foundation, and the Påhlsson Foundation, and by equipment grants from the Knut and Alice Wallenberg Foundation, the Region Skåne, Skåne University Hospital, the Linneus Foundation for the Lund University Diabetes Center and Swedish Foundation for Strategic Research for IRC15-0067.

MEC

The Multiethnic Cohort Study was supported by the National Cancer Institute (U01CA164973)

MESA

MESA and the MESA SHARe project are conducted and supported by the National Heart, Lung, and Blood Institute (NHLBI) in collaboration with MESA investigators. Support for MESA is provided by contracts 75N92020D00001, HHSN268201500003I, N01-HC-95159, 75N92020D00005, N01-HC-95160, 75N92020D00002, N01-HC-95161, 75N92020D00003, N01-HC-95162, 75N92020D00006, N01-HC-95163, 75N92020D00004, N01-HC-95164, 75N92020D00007, N01-HC-95165, N01-HC-95166, N01-HC-95167, N01-HC-95168, N01-HC-95169, UL1-TR-000040, UL1-TR-001079, UL1-TR-001420, UL1-TR-001881, and DK063491. This publication was developed under Science to Achieve Results (STAR) research assistance agreements, No. RD831697 (MESA Air), and RD-83830001 (MESA Air Next Stage), awarded by the U.S Environmental protection Agency. It has not been formally reviewed by the EPA. The views expressed in this document are solely those of the authors and the EPA does not endorse any products or commercial services mentioned in this publication.

METSIM

The METSIM study was funded by the Academy of Finland (grants no. 77299 and 124243).

NBS1

The Nijmegen Biomedical Study was funded by the Radboud university medical center

NESDA

The infrastructure for the NESDA study (www.nesda.nl) is funded through the Geestkracht program of the Netherlands Organisation for Health Research and Development (ZonMw, grant number 10-000-1002) and financial contributions by participating universities and mental health care organizations (VU University Medical Center, GGZ inGeest, Leiden University Medical Center, Leiden University, GGZ Rivierduinen, University Medical Center Groningen, University of Groningen, Lentis, GGZ Friesland, GGZ Drenthe, Rob Giel Onderzoekscentrum).

NFBC66

We thank all cohort members and researchers who participated in the 31 yrs study. We also wish acknowledge the work of the NFBC project center. NFBC1966 received financial support

from University of Oulu Grant no. 65354, Oulu University Hospital Grant no. 2/97, 8/97, Ministry of Health and Social Affairs Grant no. 23/251/97, 160/97, 190/97, National Institute for Health and Welfare, Helsinki Grant no. 54121, Regional Institute of Occupational Health, Oulu, Finland Grant no. 50621, 54231.

NHS (Nurse Health Study)

The HPFS and NHS are funded by the National Institute of Deafness and Other Communication Disorders (R03 DC013373 to M.C.C). The NHS (UM1 CA186107) and HPFS (U01 CA167552) are additionally supported by the National Cancer Institute. We thank all participants of the NHS and HPFS for their continued contributions to research.

NSPHS

We acknowledge all the participants and staff involved in NSPHS for their valuable contribution. The NSPHS was funded by the Foundation for Strategic Research and the European Commission FP6. The computations and data handling were enabled by resources provided by the Swedish National Infrastructure for Computing (SNIC) at Uppsala Multidisciplinary Center for Advanced Computational Science (UPPMAX). This work was also funded by the Swedish Research Council 2019-01497, and the Swedish Heart Lung Foundation nr. 20200687.

NTR

Netherland Twin Register: Funding was obtained from the Netherlands Organization for Scientific Research (NWO) and The Netherlands Organisation for Health Research and Development (ZonMW) grants 904-61-090, 985-10-002, 912-10-020, 904-61-193,480-04-004, 463-06-001, 451-04-034, 400-05-717, Addiction-31160008, 016-115-035, 481-08-011, 400-07-080, 056-32-010, Middelgroot-911-09-032, OCW_NWO Gravity program –024.001.003, NWO-Groot 480-15-001/674, Center for Medical Systems Biology (CSMB, NWO Genomics), NBIC/BioAssist/RK(2008.024), Biobanking and Biomolecular Resources Research Infrastructure (BBMRI –NL, 184.021.007 and 184.033.111), X-Omics 184-034-019; Spinozapremie (NWO- 56-464-14192) and KNAW Academy Professor Award (PAH/6635); Amsterdam Public Health research institute (former EMGO+) , Neuroscience Amsterdam research institute (former NCA) ; the European Community's Fifth and Seventh Framework Program (FP5- LIFE QUALITY-CT-2002-2006, FP7- HEALTH-F4-2007-2013, grant 01254: GenomEUtwin, grant 01413: ENGAGE and grant 602768: ACTION); the European Research Council (ERC Starting 284167, ERC Consolidator 771057, ERC Advanced 230374), Rutgers University Cell and DNA Repository (NIMH U24 MH068457-06), the National Institutes of Health (NIH, R01D0042157-01A1, R01MH58799-03, MH081802, DA018673, R01 DK092127-04, Grand Opportunity grants 1RC2 MH089951, and 1RC2 MH089995); the Avera Institute for Human Genetics, Sioux Falls, South Dakota (USA). Part of the genotyping and analyses were funded by the Genetic Association Information Network (GAIN) of the Foundation for the National Institutes of Health.

ORCADES
The Orkney Complex Disease Study (ORCADES) was supported by the Chief Scientist Office of the Scottish Government (CZB/4/276, CZB/4/710), a Royal Society URF to J.F.W., the MRC Human Genetics Unit quinquennial programme “QTL in Health and Disease”, Arthritis Research UK and the European Union framework program 6 EUROSPAN project (contract no. LSHG-CT-2006-018947). DNA extractions were performed at the Wellcome Trust Clinical Research Facility in Edinburgh. We would like to acknowledge the invaluable contributions of the research nurses in Orkney, the administrative team in Edinburgh and the people of Orkney.

PELOTAS1982
The 1982 Pelotas Birth Cohort Study is conducted by the Postgraduate Program in Epidemiology at Universidade Federal de Pelotas with the collaboration of the Brazilian Public Health Association (ABRASCO). From 2004 to 2013, the Wellcome Trust supported the study. The International Development Research Center, World Health Organization, Overseas Development Administration, European Union, National Support Program for Centers of Excellence (PRONEX), the Brazilian National Research Council (CNPq), and the Brazilian Ministry of Health supported previous phases of the study.
Genotyping of 1982 Pelotas Birth Cohort Study participants was supported by the Department of Science and Technology (DECIT, Ministry of Health) and National Fund for Scientific and Technological Development (FNDCT, Ministry of Science and Technology), Funding of Studies and Projects (FINEP, Ministry of Science and Technology, Brazil), Coordination of Improvement of Higher Education Personnel (CAPES, Ministry of Education, Brazil).

QFS
The Quebec Family Study (QFS) was funded by multiple grants from the Medical Research Council of Canada and the Canadian Institutes for Health Research. This work was supported by a team grant from the Canadian Institutes for Health Research (FRCN-CCT-83028)

QIMR
The QIMR cohort consists of twins recruited to two studies conducted at QIMR Berghofer Medical Research Institute: the Over 50’s (Aged) and Alcohol Cohort 1 (AL1) Studies. Twins from the Aged Study were drawn from the Australian National Health and Medical Research Council (NHMRC) Twin Registry. This work was partly supported by a donation from Mr George Landers, and benefited from funding from NHMRC to Ian B. Hickie (931215-Project Grant, and 953208-Program Grant) and Nicholas G. Martin (941177). We thank Fran Boyle and Len Roberts for their work in constructing the questionnaire, Olivia Zheng for administering the mail-out, John Pearson for data management and Nirmala Pandeya for data cleaning. The AL1 study was carried out in co-operation with the Australian Twin Registry, and was supported in part by grants from NIAA (USA) AA07535, AA013320, AA013326, and NHMRC 941177, 951023, 950998, 981339, 241916 and 941944. We are extremely grateful to all the twins who took part in these studies.

SHIP & SHIP-TREND
SHIP is part of the Community Medicine Research net of the University of Greifswald, Germany, which is funded by the Federal Ministry of Education and Research (grants no. 01ZZ9603, 01ZZ0103, and 01ZZ0403), the Ministry of Cultural Affairs as well as the Social Ministry of the Federal State of Mecklenburg-West Pomerania, and the network 'Greifswald Approach to Individualized Medicine (GANI_MED)' funded by the Federal Ministry of Education and Research (grant 03IS2061A). Genome-wide data have been supported by the Federal Ministry of Education and Research (grant no. 03ZIK012) and a joint grant from Siemens Healthineers, Erlangen, Germany and the Federal State of Mecklenburg- West Pomerania. The University of Greifswald is a member of the Caché Campus program of the InterSystems GmbH.

Singapore Chinese Health Study (SCHS)
The Singapore Chinese Health Study is supported by the National Institutes of Health, USA (RO1 CA144034 and UM1 CA182876), the nested case-control study of myocardial infarction by the Singapore National Medical Research Council (NMRC 1270/2010) and genotyping by the HUJ-CREATE Programme of the National Research Foundation, Singapore (Project Number 370062002).

SP2
The Singapore Prospective Study Program (SP2) was funded through grants from the Biomedical Research Council of Singapore (BMRC) and the National Medical Research Council of Singapore (NMRC). Genome Institute of Singapore provided services for genotyping.

STR / Twingene
The Swedish Twin Registry is managed by Karolinska Institutet and receives funding through the Swedish Research Council under the grant no 2017-00641.

TRAILS
TRAILS (TRacking Adolescents' Individual Lives Survey) is a collaborative project involving various departments of the University Medical Center and University of Groningen, the University of Utrecht, the Radboud Medical Center Nijmegen, and the Parnassia Group, all in the Netherlands. TRAILS has been financially supported by various grants from the Netherlands Organization for Scientific Research NWO (Medical Research Council program grant GB-MW 940-38-011; ZonMW Brainpower grant 100-001-004; ZonMw Risk Behavior and Dependence grants 60-60600-97-118; ZonMw Culture and Health grant 261-98-710; Social Sciences Council medium-sized investment grants GB-MaGW 480-01-006 and GB-MaGW 480-07-001; Social Sciences Council project grants GB-MaGW 452-04-314 and GB-MaGW 452-06-004; NWO large-sized investment grant 175.010.2003.005; NWO Longitudinal Survey and Panel Funding 481-08-013 and 481-11-001; NWO Vici 016.130.002 and 453-16-007/2735; NWO Gravitation 024.001.003), the Dutch Ministry of Justice (WODC), the European Science Foundation (EuroSTRESS project FP-006), the European Research Council (ERC-2017-STG-757364 en ERC-CoG-2015-681466), Biobanking and Biomolecular Resources Research Infrastructure BBMRI-NL (CP 32), the Gratama foundation, the Jan Dekker foundation, the participating universities, and Accare Centre for Child and Adolescent Psychiatry. Statistical analyses were

carried out on the Genetic Cluster Computer (http://www.geneticcluster.org), which is financially supported by the Netherlands Scientific Organization (NWO 480-05-003) along with a supplement from the Dutch Brain Foundation. We are grateful to everyone who participated in this research or worked on this project to make it possible.

TwinsUK

TwinsUK is funded by the Wellcome Trust, Medical Research Council, European Union, the National Institute for Health Research (NIHR)-funded BioResource, Clinical Research Facility and Biomedical Research Centre based at Guy's and St Thomas' NHS Foundation Trust in partnership with King's College London.

VIS

We would like to acknowledge the staff of several institutions in Croatia that supported the field work, including but not limited to The University of Split and Zagreb Medical Schools, the Institute for Antroplogical Research in Zagreb and the Croatian Institute for Public Health. We would like to acknowledge the invaluable contributions of the recruitment team in Korcula, the administrative teams in Croatia and Edinburgh and the participants. The SNP genotyping was performed in the core genotyping laboratory of the Wellcome Trust Clinical Research Facility at the Western General Hospital, Edinburgh, Scotland. The study was funded by the Medical Research Council UK, the European Union framework program 6 EUROSPAN project (contract no. LSHG-CT-2006-018947), the Croatian National Centre of Research Excellence in Personalized Healthcare grant (number KK.01.1.1.01.0010), and the Centre of Competence in Molecular Diagnostics (KK.01.2.2.03.0006).

WGHS

The WGHS is funded by grants from the NHLBI (HL043851 and HL080467) and NCI (CA047988 and UM1CA182913), with funding for genotyping provided by Amgen.

WHI

WHI: The WHI program is funded by the National Heart, Lung, and Blood Institute, National Institutes of Health, U.S. Department of Health and Human Services through contracts 75N92021D00001, 75N92021D00002, 75N92021D00003, 75N92021D00004, 75N92021D00005. A listing of WHI investigators can be found at: https://www.whi.org/researchers/Documents%20%20Write%20a%20Paper/WHI%20Investigator%20Long%20List.pdf.

YFS

The Young Finns Study has been financially supported by the Academy of Finland: grants 322098, 286284, 134309 (Eye), 126925, 121584, 124282, 129378 (Salve), 117787 (Gendi), and 41071 (Skidi); the Social Insurance Institution of Finland; Competitive State Research Financing of the Expert Responsibility area of Kuopio, Tampere and Turku University Hospitals (grant X51001); Juho Vainio Foundation; Paavo Nurmi Foundation; Finnish Foundation for Cardiovascular Research ; Finnish Cultural Foundation; The Sigrid Juselius Foundation; Tampere Tuberculosis Foundation; Emil Aaltonen Foundation; Yrjö Jahnsson Foundation; Signe and Ane Gyllenberg Foundation; Diabetes Research Foundation of Finnish Diabetes

Association; EU Horizon 2020 (grant 755320 for TAXINOMISIS; grant 848146 for To_Aition); European Research Council (grant 742927 for MULTIEPIGEN project); and Tampere University Hospital Supporting Foundation.
We thank the teams that collected data at all measurement time points; the persons who participated as both children and adults in these longitudinal studies; and biostatisticians Irina Lisinen, Johanna Ikonen, Noora Kartiosuo, Ville Aalto, and Jarno Kankaanranta for data management and statistical advice.

SUPPLEMENTARY NOTE REFERENCES

1. Pontzer, H., Wood, B.M. & Raichlen, D.A. Hunter-gatherers as models in public health. *Obes Rev* **19 Suppl 1**, 24-35 (2018).
2. Grossman, S.R. *et al.* Identifying recent adaptations in large-scale genomic data. *Cell* **152**, 703-13 (2013).
3. Mathieson, I. *et al.* Genome-wide patterns of selection in 230 ancient Eurasians. *Nature* **528**, 499-503 (2015).
4. Field, Y. *et al.* Detection of human adaptation during the past 2000 years. *Science* **354**, 760-764 (2016).
5. Krueger, D.D., Osterweil, E.K. & Bear, M.F. Activation of mGluR5 induces rapid and long-lasting protein kinase D phosphorylation in hippocampal neurons. *J Mol Neurosci* **42**, 1-8 (2010).
6. Rhee, S.G. Regulation of phosphoinositide-specific phospholipase C. *Annu Rev Biochem* **70**, 281-312 (2001).
7. Zhu, X. *et al.* Phospholipase Cε deficiency delays the early stage of cutaneous wound healing and attenuates scar formation in mice. *Biochem Biophys Res Commun* **484**, 144-151 (2017).
8. Marck, A. *et al.* Age-Related Changes in Locomotor Performance Reveal a Similar Pattern for Caenorhabditis elegans, Mus domesticus, Canis familiaris, Equus caballus, and Homo sapiens. *J Gerontol A Biol Sci Med Sci* **72**, 455-463 (2017).
9. Norheim, F. *et al.* Gene-by-Sex Interactions in Mitochondrial Functions and Cardio-Metabolic Traits. *Cell metabolism* **29**, 932-949.e4 (2019).
10. Hillis, D.A. *et al.* Genetic Basis of Aerobically Supported Voluntary Exercise: Results from a Selection Experiment with House Mice. *Genetics* **216**, 781-804 (2020).
11. Battle, A., Brown, C.D., Engelhardt, B.E. & Montgomery, S.B. Genetic effects on gene expression across human tissues. *Nature* **550**, 204-213 (2017).
12. Lightfoot, J.T. Sex hormones' regulation of rodent physical activity: a review. *International journal of biological sciences* **4**, 126-132 (2008).
13. Nguyen, Q.A.T. *et al.* Coadaptation of the chemosensory system with voluntary exercise behavior in mice. *PLoS One* **15**, e0241758 (2020).
14. Panter, J. *et al.* Using alternatives to the car and risk of all-cause, cardiovascular and cancer mortality. *Heart* **104**, 1749-1755 (2018).
15. Kikuchi, H. *et al.* Occupational sitting time and risk of all-cause mortality among Japanese workers. *Scand J Work Environ Health* **41**, 519-28 (2015).
16. Vink, J.M. *et al.* Variance components models for physical activity with age as modifier: a comparative twin study in seven countries. *Twin Research and Human Genetics* **14**, 25-34 (2011).
17. Day, F.R., Loh, P.R., Scott, R.A., Ong, K.K. & Perry, J.R. A Robust Example of Collider Bias in a Genetic Association Study. *Am J Hum Genet* **98**, 392-3 (2016).
18. McLaren, W. *et al.* The Ensembl Variant Effect Predictor. *Genome Biology* **17**, 122 (2016).
19. Gogarten, S.M. *et al.* Genetic association testing using the GENESIS R/Bioconductor package. *Bioinformatics* (2019).

20. Lusis, A.J. *et al.* The Hybrid Mouse Diversity Panel: a resource for systems genetics analyses of metabolic and cardiovascular traits. *J Lipid Res* **57**, 925-42 (2016).
21. Gautel, M. & Djinović-Carugo, K. The sarcomeric cytoskeleton: from molecules to motion. *J Exp Biol* **219**, 135-45 (2016).
22. Luther, P.K. The vertebrate muscle Z-disc: sarcomere anchor for structure and signalling. *J Muscle Res Cell Motil* **30**, 171-85 (2009).
23. Luther, P.K. Three-dimensional reconstruction of a simple Z-band in fish muscle. *J Cell Biol* **113**, 1043-55 (1991).
24. Grison, M., Merkel, U., Kostan, J., Djinović-Carugo, K. & Rief, M. α-Actinin/titin interaction: A dynamic and mechanically stable cluster of bonds in the muscle Z-disk. *Proc Natl Acad Sci U S A* **114**, 1015-1020 (2017).
25. Pickering, C. & Kiely, J. ACTN3: More than Just a Gene for Speed. *Frontiers in Physiology* **8**(2017).
26. Norman, B. *et al.* Strength, power, fiber types, and mRNA expression in trained men and women with different ACTN3 R577X genotypes. *J Appl Physiol (1985)* **106**, 959-65 (2009).
27. Mallakin, A. *et al.* Gene expression profiles of Mst1r-deficient mice during nickel-induced acute lung injury. *Am J Respir Cell Mol Biol* **34**, 15-27 (2006).
28. Wang, M.H. *et al.* Identification of the ron gene product as the receptor for the human macrophage stimulating protein. *Science* **266**, 117-9 (1994).
29. Nanney, L.B. *et al.* Proteolytic cleavage and activation of pro-macrophage-stimulating protein and upregulation of its receptor in tissue injury. *J Invest Dermatol* **111**, 573-81 (1998).
30. Feres, K.J., Ischenko, I. & Hayman, M.J. The RON receptor tyrosine kinase promotes MSP-independent cell spreading and survival in breast epithelial cells. *Oncogene* **28**, 279-88 (2009).
31. Klimentidis, Y.C. *et al.* Genome-wide association study of habitual physical activity in over 377,000 UK Biobank participants identifies multiple variants including CADM2 and APOE. *International Journal of Obesity* **42**, 1161-1176 (2018).
32. Scott, J.D., Dessauer, C.W. & Taskén, K. Creating order from chaos: cellular regulation by kinase anchoring. *Annu Rev Pharmacol Toxicol* **53**, 187-210 (2013).
33. Tingley, W.G. *et al.* Gene-trapped mouse embryonic stem cell-derived cardiac myocytes and human genetics implicate AKAP10 in heart rhythm regulation. *Proc Natl Acad Sci U S A* **104**, 8461-6 (2007).
34. Dessauer, C.W. Adenylyl cyclase--A-kinase anchoring protein complexes: the next dimension in cAMP signaling. *Mol Pharmacol* **76**, 935-41 (2009).
35. Berdeaux, R. & Stewart, R. cAMP signaling in skeletal muscle adaptation: hypertrophy, metabolism, and regeneration. *American journal of physiology. Endocrinology and metabolism* **303**, E1-E17 (2012).
36. Zhu, Q. *et al.* KDM4A regulates myogenesis by demethylating H3K9me3 of myogenic regulatory factors. *Cell Death Dis* **12**, 514 (2021).
37. Van Rompay, A.R., Johansson, M. & Karlsson, A. Identification of a novel human adenylate kinase. cDNA cloning, expression analysis, chromosome localization and characterization of the recombinant protein. *Eur J Biochem* **261**, 509-17 (1999).

38. Debray, F.G. *et al.* LRPPRC mutations cause a phenotypically distinct form of Leigh syndrome with cytochrome c oxidase deficiency. *J Med Genet* **48**, 183-9 (2011).
39. Song, S.-Y. *et al.* Environmental Enrichment Upregulates Striatal Synaptic Vesicle-Associated Proteins and Improves Motor Function. *Frontiers in neurology* **9**, 465-465 (2018).
40. Dickinson, D. *et al.* Differential Effects of Common Variants in SCN2A on General Cognitive Ability, Brain Physiology, and messenger RNA Expression in Schizophrenia Cases and Control Individuals. *JAMA Psychiatry* **71**, 647-656 (2014).
41. Tatsukawa, T. *et al.* Scn2a haploinsufficient mice display a spectrum of phenotypes affecting anxiety, sociability, memory flexibility and ampakine CX516 rescues their hyperactivity. *Molecular Autism* **10**, 15 (2019).
42. Kamura, T. *et al.* Cytoplasmic ubiquitin ligase KPC regulates proteolysis of p27(Kip1) at G1 phase. *Nat Cell Biol* **6**, 1229-35 (2004).
43. Hara, T. *et al.* Role of the UBL-UBA protein KPC2 in degradation of p27 at G1 phase of the cell cycle. *Mol Cell Biol* **25**, 9292-303 (2005).
44. Wang, S. *et al.* RNF123 has an E3 ligase-independent function in RIG-I-like receptor-mediated antiviral signaling. *EMBO Rep* **17**, 1155-68 (2016).
45. Johnston, K.J.A. *et al.* Genome-wide association study of multisite chronic pain in UK Biobank. *PLOS Genetics* **15**, e1008164 (2019).
46. Rahman, M.S. *et al.* Genome-wide association study identifies RNF123 locus as associated with chronic widespread musculoskeletal pain. *Ann Rheum Dis* (2021).
47. Okbay, A. *et al.* Genome-wide association study identifies 74 loci associated with educational attainment. *Nature* **533**, 539-542 (2016).
48. Strawbridge, R.J. *et al.* Genome-wide analysis of self-reported risk-taking behaviour and cross-disorder genetic correlations in the UK Biobank cohort. *Transl Psychiatry* **8**, 39 (2018).
49. Clarke, T.K. *et al.* Genome-wide association study of alcohol consumption and genetic overlap with other health-related traits in UK Biobank (N=112 117). *Mol Psychiatry* **22**, 1376-1384 (2017).
50. Pasman, J.A. *et al.* GWAS of lifetime cannabis use reveals new risk loci, genetic overlap with psychiatric traits, and a causal influence of schizophrenia. *Nat Neurosci* **21**, 1161-1170 (2018).
51. Locke, A.E. *et al.* Genetic studies of body mass index yield new insights for obesity biology. *Nature* **518**, 197-206 (2015).
52. Yan, X. *et al.* Cadm2 regulates body weight and energy homeostasis in mice. *Molecular metabolism* **8**, 180-188 (2018).
53. Lee, S.-H. *et al.* REEP5 depletion causes sarco-endoplasmic reticulum vacuolization and cardiac functional defects. *Nature Communications* **11**, 965 (2020).
54. Ichhaporia, V.P. *et al.* SIL1, the endoplasmic-reticulum-localized BiP co-chaperone, plays a crucial role in maintaining skeletal muscle proteostasis and physiology. *Disease Models & Mechanisms* **11**(2018).
55. Chen, J., Zook, D., Crickard, L. & Tabatabaei, A. Effect of phosphodiesterase (1B, 2A, 9A and 10A) inhibitors on central nervous system cyclic nucleotide levels in rats and mice. *Neurochem Int* **129**, 104471 (2019).

56. Greengard, P. The neurobiology of slow synaptic transmission. *Science* **294**, 1024-30 (2001).
57. Threlfell, S., Sammut, S., Menniti, F.S., Schmidt, C.J. & West, A.R. Inhibition of Phosphodiesterase 10A Increases the Responsiveness of Striatal Projection Neurons to Cortical Stimulation. *J Pharmacol Exp Ther* **328**, 785-95 (2009).
58. Burri, L. *et al.* Mature DIABLO/Smac is produced by the IMP protease complex on the mitochondrial inner membrane. *Mol Biol Cell* **16**, 2926-33 (2005).
59. Inoue, M., Chang, L., Hwang, J., Chiang, S.H. & Saltiel, A.R. The exocyst complex is required for targeting of Glut4 to the plasma membrane by insulin. *Nature* **422**, 629-33 (2003).
60. Borowiec, M. *et al.* Mutations at the BLK locus linked to maturity onset diabetes of the young and β-cell dysfunction. *Proceedings of the National Academy of Sciences* **106**, 14460-14465 (2009).
61. Wan, L. *et al.* PACS-1 defines a novel gene family of cytosolic sorting proteins required for trans-Golgi network localization. *Cell* **94**, 205-216 (1998).
62. Schuurs-Hoeijmakers, J.H.M. *et al.* Recurrent de novo mutations in PACS1 cause defective cranial-neural-crest migration and define a recognizable intellectual-disability syndrome. *American journal of human genetics* **91**, 1122-1127 (2012).
63. Macedo-Souza, L.I. *et al.* Spastic paraplegia, optic atrophy, and neuropathy is linked to chromosome 11q13. *Ann Neurol* **57**, 730-7 (2005).
64. Melo, U.S. *et al.* Overexpression of KLC2 due to a homozygous deletion in the non-coding region causes SPOAN syndrome. *Hum Mol Genet* **24**, 6877-85 (2015).
65. Alvarez, C., Garcia-Mata, R., Brandon, E. & Sztul, E. COPI recruitment is modulated by a Rab1b-dependent mechanism. *Mol Biol Cell* **14**, 2116-27 (2003).
66. Richardson, T.G. *et al.* Evaluating the relationship between circulating lipoprotein lipids and apolipoproteins with risk of coronary heart disease: A multivariable Mendelian randomisation analysis. *PLoS Med* **17**, e1003062 (2020).
67. Nielsen, J.B. *et al.* Loss-of-function genomic variants highlight potential therapeutic targets for cardiovascular disease. *Nature Communications* **11**, 6417 (2020).
68. Pulit, S.L. *et al.* Meta-analysis of genome-wide association studies for body fat distribution in 694 649 individuals of European ancestry. *Hum Mol Genet* **28**, 166-174 (2019).
69. Girard, F., Venail, J., Schwaller, B. & Celio, M.R. The EF-hand Ca2+-binding protein super-family: A genome-wide analysis of gene expression patterns in the adult mouse brain. *Neuroscience* **294**, 116-155 (2015).
70. Lightfoot, J.T. *et al.* Biological/Genetic Regulation of Physical Activity Level: Consensus from GenBioPAC. *Medicine and science in sports and exercise* **50**, 863-873 (2018).
71. Rhodes, J.S., Gammie, S.C. & Garland, T., Jr. Neurobiology of Mice Selected for High Voluntary Wheel-running Activity1. *Integrative and Comparative Biology* **45**, 438-455 (2005).
72. Roberts, M.D. *et al.* Nucleus accumbens neuronal maturation differences in young rats bred for low versus high voluntary running behaviour. *The Journal of physiology* **592**, 2119-2135 (2014).

73. Levay, K. & Slepak, V.Z. Tescalcin is an essential factor in megakaryocytic differentiation associated with Ets family gene expression. *J Clin Invest* **117**, 2672-83 (2007).
74. Shiflett, M.W. & Balleine, B.W. Contributions of ERK signaling in the striatum to instrumental learning and performance. *Behavioural brain research* **218**, 240-247 (2011).
75. Gupta, G.D. *et al.* A Dynamic Protein Interaction Landscape of the Human Centrosome-Cilium Interface. *Cell* **163**, 1484-99 (2015).
76. Lotta, L.A. *et al.* Integrative genomic analysis implicates limited peripheral adipose storage capacity in the pathogenesis of human insulin resistance. *Nature genetics* **49**, 17-26 (2017).
77. Bowen, T.S., Schuler, G. & Adams, V. Skeletal muscle wasting in cachexia and sarcopenia: molecular pathophysiology and impact of exercise training. *Journal of cachexia, sarcopenia and muscle* **6**, 197-207 (2015).
78. Kim, S.K. Identification of 613 new loci associated with heel bone mineral density and a polygenic risk score for bone mineral density, osteoporosis and fracture. *PLOS ONE* **13**, e0200785 (2018).
79. Wang, X. *et al.* CELF4 Variant and Anthracycline-Related Cardiomyopathy: A Children's Oncology Group Genome-Wide Association Study. *J Clin Oncol* **34**, 863-70 (2016).
80. Wilker, E. & Yaffe, M.B. 14-3-3 Proteins--a focus on cancer and human disease. *J Mol Cell Cardiol* **37**, 633-42 (2004).
81. Francis, H.M., Mirzaei, M., Pardey, M.C., Haynes, P.A. & Cornish, J.L. Proteomic analysis of the dorsal and ventral hippocampus of rats maintained on a high fat and refined sugar diet. *PROTEOMICS* **13**, 3076-3091 (2013).
82. Wei, Y. *et al.* Long-term moderate exercise enhances specific proteins that constitute neurotrophin signaling pathway: A TMT-based quantitative proteomic analysis of rat plasma. *Journal of Proteomics* **185**, 39-50 (2018).
