# Supplementary material for: Genome-wide association analyses of physical activity and sedentary behavior provide insights into underlying mechanisms and roles in disease prevention
Source: Nat Genet. 2022 Sep 7;54(9):1332–44. doi: 10.1038/s41588-022-01165-1 (PMC9470530; doi:10.1038/s41588-022-01165-1)
Supplement: Supplementary file 2 — Reporting Summary [file 41588_2022_1165_MOESM2_ESM.pdf]

nature portfolio

Corresponding author(s): Marcel den Hoed, Wang Zhe

Last updated by author(s): June 8, 2022

Reporting Summary

Nature Portfolio wishes to improve the reproducibility of the work that we publish. This form provides structure for consistency and transparency in reporting. For further information on Nature Portfolio policies, see our Editorial Policies and the Editorial Policy Checklist.

Statistics

For all statistical analyses, confirm that the following items are present in the figure legend, table legend, main text, or Methods section.

n/a	Confirmed	
☐	☒	The exact sample size (*n*) for each experimental group/condition, given as a discrete number and unit of measurement
☐	☒	A statement on whether measurements were taken from distinct samples or whether the same sample was measured repeatedly
☐	☒	The statistical test(s) used AND whether they are one- or two-sided<br>*Only common tests should be described solely by name; describe more complex techniques in the Methods section.*
☐	☒	A description of all covariates tested
☐	☒	A description of any assumptions or corrections, such as tests of normality and adjustment for multiple comparisons
☐	☒	A full description of the statistical parameters including central tendency (e.g. means) or other basic estimates (e.g. regression coefficient) AND variation (e.g. standard deviation) or associated estimates of uncertainty (e.g. confidence intervals)
☐	☒	For null hypothesis testing, the test statistic (e.g. *F*, *t*, *r*) with confidence intervals, effect sizes, degrees of freedom and *P* value noted<br>*Give P values as exact values whenever suitable.*
☐	☒	For Bayesian analysis, information on the choice of priors and Markov chain Monte Carlo settings
☒	☐	For hierarchical and complex designs, identification of the appropriate level for tests and full reporting of outcomes
☐	☒	Estimates of effect sizes (e.g. Cohen's *d*, Pearson's *r*), indicating how they were calculated

Our web collection on statistics for biologists contains articles on many of the points above.

Software and code

Policy information about availability of computer code

Data collection	Genotype calling and imputation were performed by 51 contributing studies using software specified for each study in Supp Table 2.<br>Genetic association analyses were performed by 51 contributing studies using software specified for each study in Supp Table 2.
Data analysis	GWAS in UK Biobank were conducted using BOLT-LMM v2.3.2.<br>GWAS meta-analyses were performed using METAL, version 2017-12-21.<br>Variants' effects on protein function were predicted using Ensembl's Variant Effect Predictor (VEP)<br>Gene-based burden and SKAT tests were performed using the GENESIS package version 3.15.<br>Previously reported summary statistics were extracted from the GWAS catalog, PhenoScanner V2, MRC IEU OpenGWAS.<br>Chip-based heritability was quantified using the BOLT-LMM v2.3.3 software.<br>Joint and conditional SNP association analyses were performed using GCTA version 1.92.<br>Polygenic score analyses were performed using PRSice software v2.<br>Genome-wide genetic correlations were examined using LS score regression implemented in the LD-hub web resource.<br>Mendelian randomisation analyses were performed using R packages TwoSampleMR, MVMR, MR-PRESSO, CAUSE V1.2.0.<br>Candidate genes and enriched tissues were identified using DEPICT version 1 release 194.<br>Enriched cell types were identified using CELLECT.<br>Variants with a high posterior probability of being causal were identified using FINEMAP v 1.4.1.<br>Relevant HiC data in the brain were extracted using FUMA v1.3.5.<br>VitalView Activity Software v 1.5 to calculate wheel running revolutions in mice.<br>The UCSC genome-browser and GTEx IGV browser were used to explore tissue-specific expression of the human orthologue of the mouse gene 4930413E15Rik.<br>A homology model of the E635 variant monomeric filament was generated using Phyre2 v 2.0.<br>Molecular dynamics (MD) system preparation and simulation was conducted with GROMACS 2020 and mdanalysis v 2.0.<br>The MD topology was created with GROMACS pdb2gmx using the ACTN3 dimer model and parameterized with the CHARMM36 all-atom force

field.

For manuscripts utilizing custom algorithms or software that are central to the research but not yet described in published literature, software must be made available to editors and reviewers. We strongly encourage code deposition in a community repository (e.g. GitHub). See the Nature Portfolio guidelines for submitting code & software for further information.

Data

Policy information about availability of data

All manuscripts must include a data availability statement. This statement should provide the following information, where applicable:

- Accession codes, unique identifiers, or web links for publicly available datasets
- A description of any restrictions on data availability
- For clinical datasets or third party data, please ensure that the statement adheres to our policy

European and multi-ancestry meta-analyses summary statistics for the genome-wide association study are available through the NHGRI-EBI GWAS Catalog (https://www.ebi.ac.uk/gwas/downloads/summary-statistics).
UK Biobank individual-level data can be obtained through a data access application available at https://www.ukbiobank.ac.uk/.
In this study we made use of data made available by: MetaMex: https://www.metamex.eu/; Tabula muris: https://www.czbiohub.org/tabula-muris/; Open GWAS: https://gwas.mrcieu.ac.uk/; MR Base: https://www.mrbase.org/; GTEx Consortium: https://gtexportal.org/home/; eQTLGen Consortium: https://www.eqtlgen.org/; CommonMind Consortium: https://www.synapse.org/#!Synapse:syn2759792/wiki/69613; Brain zQTLServe: http://mostafavilab.stat.ubc.ca/xqtl/; MetaBrain: https://www.metabrain.nl/

Field-specific reporting

Please select the one below that is the best fit for your research. If you are not sure, read the appropriate sections before making your selection.

☒ Life sciences ☐ Behavioural & social sciences ☐ Ecological, evolutionary & environmental sciences

For a reference copy of the document with all sections, see nature.com/documents/nr-reporting-summary-flat.pdf

Life sciences study design

All studies must disclose on these points even when the disclosure is negative.

Sample size

To ensure the highest possible statistical power to identify robust genetic associations with physical activity and sedentary traits, we aimed to include data from any and all studies with relevant data that were willing and able to participate. This resulted in a sample size of up to 674,980 individuals from 51 studies. For external validation using genetic predisposition scores, we used physical activity data from 8,195 participants of the BioMe Biobank that were available to us. While this sample size is not sufficient to validate associations for individual variants, it is adequate to examine associations of complex traits with genetic predisposition scores. Molecular dynamics simulations were run in triplicate to ensure results are robust. Single fiber experiments were performed in 298 single muscle fibers from four R/R and four X/X carriers at the ACTN3 R577X variant, of which one R/R carrier is an E/E carrier and three are E/A carriers at the ACTN3 E635A variant of interest.

Data exclusions

Standard quality control based on genotype and sample quality was performed at the study level, as well as at the meta-analysis level (as described in Winkler et al., 2014).

Replication

The robustness of findings from the meta-analysis of GWAS for physical activity and sedentary traits was examined in two ways. Firstly, by examining the associations of individual variants identified as being associated with self-reported physical activity and sedentary traits in our study with objectively assessed physical activity traits in UK Biobank data (which makes up a large part of the data in the meta-analysis). Roughly half of the identified loci showed such associations at $P<0.05$. Secondly, we used data from participants of the BioMe Biobank to examine associations with moderate-to-vigorous intensity leisure time activity for genetic predisposition scores consisting of loci associated with moderate-to-vigorous intensity leisure time activity and leisure screen time identified in our meta-analysis. Both predisposition scores showed significant associations with moderate-to-vigorous intensity leisure time activity in the independent replication data of BioMe.

Randomization

With the exception of the single fiber experiments, there were no experimental groups in this study. In the single fiber experiments, eight healthy young men that volunteered to donate a muscle biopsy were selected from a group of 266 participants based on characteristics including height, weight, physical activity level and maximal knee extension torque at 45 degrees flexion. Participant were selected in such a way that four R/R carriers at the ACTN3 R577X variant were matched to four X/X carriers, as described in more detail in Broos et al., 2016 (PMID 26930663). The physician obtaining the muscle biopsy was unaware of the R577X genotype, the researchers performing the single fiber expriments were aware of the R577X genotype of the donot, but the variant of interest in the current study (the ACTN3 E635A-encoding variant) was not at the time anticipated to be relevant and was only genotyped in 2021 for the current study. Hence, the researchers that performed the single fiber experiments were blinded to the main exposure of interest in the present study.

Blinding

GWAS is a hypothesis-free approach, so in each study contributing to the meta-analysis, researchers assessing physical activity and sedentary traits were blinded to the genotypes that we now know are associated with those outcomes.

Similarly, in the GWAS performed in 100 mouse strains, associations analyses were performed hypothesis-free, and researchers were not aware of the loci identified as being associated with physical activity traits in humans.

In the single muscle fiber experiments, the researchers were aware of the genotype at the ACTN3 R577X variant at the time of the study, but they were blinded to the participants' genotype at the variant of interest in the present study, i.e. the ACTN3 E635A-encoding variant, which was de novo genotyped for this study.

Reporting for specific materials, systems and methods

We require information from authors about some types of materials, experimental systems and methods used in many studies. Here, indicate whether each material, system or method listed is relevant to your study. If you are not sure if a list item applies to your research, read the appropriate section before selecting a response.

Materials & experimental systems

n/a	Involved in the study
☒	☐ Antibodies
☒	☐ Eukaryotic cell lines
☒	☐ Palaeontology and archaeology
☐	☒ Animals and other organisms
☐	☒ Human research participants
☒	☐ Clinical data
☒	☐ Dual use research of concern

Methods

n/a	Involved in the study
☒	☐ ChIP-seq
☒	☐ Flow cytometry
☒	☐ MRI-based neuroimaging

Animals and other organisms

Policy information about studies involving animals; ARRIVE guidelines recommended for reporting animal research

Laboratory animals: Mice arrived at UCLA at 5 to 8 weeks of age and were housed 1-4 weeks until wheel testing. All mice were ~3 months old at the start of the experimental protocol, and were randomized into two groups: 1) sedentary or no exercise; and 2) exercise trained. Strains used and sample size per group are shown in Supp Table 23. Trained animals were housed unaccompanied on a standard 12-hour light dark cycle (6AM to 6PM local time). They were fed on a standard laboratory chow diet (8604, Teklad) with ad libitum access to food and water for the entire duration of the experiment. Mice were given full-time access to a running wheel for ~30 days.

Wild animals: The study did not involve wild animals.

Field-collected samples: The study did not involve samples collected from the field.

Ethics oversight: For the GWAS in 100 mouse strains, all studies were approved by the Institutional Animal Care and Use Committee (IACUC) and the Animal Research Committee (ARC # 1992-169-83e) at the University of California, Los Angeles (UCLA).

Note that full information on the approval of the study protocol must also be provided in the manuscript.

Human research participants

Policy information about studies involving human research participants

Population characteristics: Study level genome-wide association studies were run in a sex and ancestry specific manner, and were adjusted for age, age-squared, principal components reflecting population structure and additional study-specific covariates where appropriate. Since we used data from men and women of 51 studies, we kindly refer to supplementary table 1 for descriptive information on age and sex in each study.

Recruitment: Participants were required in the 51 studies contributing to the meta-analysis as described in protocol papers for those studies. To identify studies with data on both genome-wide genotypes and physical activity and sedentary behavior, we contacted all cohort studies we were aware of at the time, benefiting from earlier collaborations and meta-analyses of GWAS performed and published by others.

Ethics oversight: No ethical approval was required for the study since it was a meta-analysis of summary statistics obtained in studies that each had ethical approval provided by local ethics boards.

Note that full information on the approval of the study protocol must also be provided in the manuscript.
